# Peptide Mass Fingerprinting of South American Xenarthrans: A New Resource for Zooarcheology and Palaeontology

**DOI:** 10.1021/acs.jproteome.5c00636

**Published:** 2025-11-03

**Authors:** Mariya Antonosyan, Roshan Paladugu, Michael Ziegler, Gabriela Prestes Carneiro, Eliane Chim, Andre Menezes Strauss, Diego Mendes, Rafael Lemos, Jorge Domingo Carrillo-Briceño, Laura Pereira Furquim, Stefanie Schirmer, Jana Ilgner, Daniela Volke, Patrick Roberts

**Affiliations:** † Department of Coevolution of Land Use and Urbanisation, Max Planck Institute of Geoanthropology, 07745 Jena, Germany; ‡ Department of Evolutionary Genetics & aDNA Sequencing Core Unit, 28305 Max Planck Institute for Evolutionary Anthropology, Deutscher Platz 6, 04103 Leipzig, Germany; § National Museum of Natural History, 75005 Paris, France; ∥ Museum of Archeology and Ethnology, University of São Paulo, 05508-070 São Paulo, Brazil; ⊥ Anthropological Museum of the Federal University of Goiás, 74605-010 Goiânia, Brazil; # Paleontological Institute, 27217 University of Zurich, 8006 Zurich, Switzerland; ∇ Laboratory Central Unit, Max Planck Institute of Geoanthropology, 07745 Jena, Germany; ○ Center for Biotechnology and Biomedicine (BBZ), 9180 University of Leipzig, 04103 Leipzig, Germany

**Keywords:** palaeoproteomics, collagen fingerprinting, neotropics

## Abstract

Xenarthrans—armadillos, anteaters, and sloths—are endemic to the Americas, primarily inhabiting the Neotropics, where they represent a key component of faunal diversity. They have essential functions for ecosystem maintenance, such as insect control and nutrient cycling, playing key roles as ecosystem engineers. Despite their frequent occurrence in archeological and paleontological contexts, their identification is often hindered by the highly fragmented and morphologically indistinct nature of bone remains. This limits our ability to track their biogeographic histories, population dynamics, and interactions with past human populations. To address this, we present a novel set of Zooarcheology by Mass Spectrometry (ZooMS) peptide markers for ten extant and extinct Xenarthran species, enabling taxonomic identification of fragmented and morphologically indistinct bone assemblages. By enhancing the taxonomic resolution of fragmented faunal material, this work advances the reconstruction of past species distributions, long-term biodiversity trends, and human–animal interactions. Furthermore, it provides a foundation for an improved understanding of Xenarthran extinction and adaptation dynamics and can support conservation and ecosystem restoration efforts by informing models of historical biogeography and species abundance.

## Graphical abstract

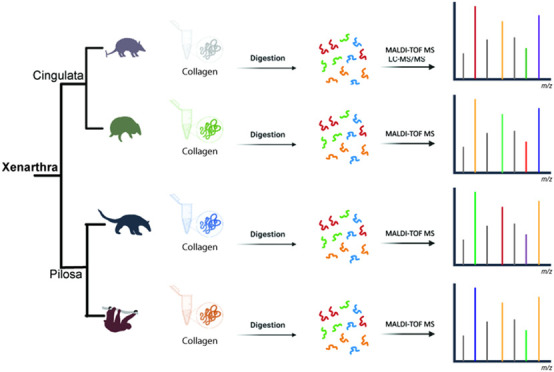

## Introduction

The mammalian order Xenarthra represents one of the four major clades of placental mammals. The order is composed of morphologically and ecologically distinct suborders: Cingulata, represented by armadillos (Dasypodidae), and Pilosa, represented by anteaters (Vermilingua) and sloths (Folivora).^1,2^ They are unique among mammals in exhibiting xenarthrous vertebrae, dermal ossification, absence of enamel on adult teeth, and low metabolic rates.^3^ The Neotropics are home to a rich diversity of Xenarthran species, including 21 armadillos, 10 anteaters, and 6 sloths, each adapted to a range of ecological niches.^4^ While many species are specialized for either forested or open habitats, some taxa exhibit a continental range and can be found in diverse habitats.^5^ Armadillos, sloths, and anteaters play vital roles in maintaining healthy ecosystems across the Neotropics, acting as ecosystem engineers. These include bioturbation by armadillos, which aerates and mixes soils; pest control by insectivorous anteaters; contributions to nutrient cycling and forest dynamics by sloths.^6,7^ The living species of the order Xenarthra are relics of South America’s Tertiary radiation, which gave rise to giant extinct forms, including more than 180 now-extinct genera such as glyptodonts and ground-dwelling sloths.^8^ These megafaunal Xenarthrans underwent extinction during the terminal Pleistocene, coinciding with major climatic shifts and increasing evidence of human presence.^9–11^ Despite extensive research into the extinction dynamics of Pleistocene Xenarthran megafauna (e.g.,^12–14^ ), the precise timing and causes of these extinctions, whether driven by human activities or environmental change, remain unresolved.

Evident through a plethora of cultural motifs and reported in historical and ethnographic studies, Xenarthran species play a prominent role in the indigenous cultures of South America.^15–20^ Armadillos have been subjects of subsistence hunting, a centuries-old practice^21–24^ that continues to this day.^25,26^ The osteological elements of armadillos have been used to make musical instruments, smoking pipes, baskets, and ceremonial objects.^27,28^ In the case of sloths, some communities, such as the Acawai, Witotoans, and Sioní, regularly hunt and consume sloths.^29–33^ In contrast, others, including the Piro, Arawakan, and Yukuí, largely abhor sloth hunting, often citing the poor taste of the meat or cultural revulsion.^33–35^ Despite limited dietary use in some regions, sloth hides and body parts are used for adornment, ritual objects, and medicinal applications, including contemporary interest in algae and compounds found on their skin.^35–40^ Similar to armadillos, anteaters have been the subjects of capture and hunting for illegal trafficking and meat.^41^ Widespread cultural taboos in indigenous communities, a unique factor negatively affecting anteaters, especially *Myrmecophaga tridactyla*, are a major documented cause of anteater mortalities.^17,42^

Archaeological and palaeontological records from South America have recovered a wide array of Xenarthran remains dating from the Late Pleistocene to historical times.^13,14,43–49^ Research on these specimens can offer valuable insight into early human activities including hunting practices, subsistence strategies, and cultural uses of these animals. Moreover, the study of Xenarthran remains can contribute to reconstructing past environmental conditions, shifts in biodiversity over time, and changes in their ecological adaptations and help assess the impact of past climate change and human activities. However, a major obstacle to this process is the high degree of bone fragmentation and poor preservation commonly observed in archeological assemblages. These conditions often pose a significant challenge to taxonomic identification in zooarcheological assemblages, limiting resolution to broad categories, such as family or body size. As a result, substantial biological information embedded in nondiagnostic bone fragments is routinely lost. Advances in biomolecular methods, particularly palaeoproteomics, now offer powerful tools to recover these data, enhancing both the precision and quantity of taxonomic identifications and thereby refining reconstructions of past biodiversity.^50–52^

One such palaeoproteomic technique is collagen peptide mass fingerprinting, also known as zooarcheology by mass spectrometry (ZooMS), which allows taxonomic affiliation of archeological bone fragments based on enzymatically digested Collagen type I (COL1) peptide masses.^53^ Collagen type I is a quaternary structure composed of two COL1α1 chains and one COL1α2 chain in mammals, birds, reptiles, and amphibians. Taxon-specific nucleotide variations in COL1A1 and COL1A2 genes lead to slightly different amino acid sequences of collagen type I in different taxa. The primary principle of ZooMS is to generate a peptide mass fingerprint from tryptic digests of bone or other collagen-containing tissues using a matrix-assisted laser desorption/ionization time-of-flight (MALDI-ToF) mass spectrometer.^53^ A critical prerequisite for successful ZooMS-based taxonomic identification is the availability of a comprehensive reference database of collagen peptide markers. To date, such libraries have been largely developed for Eurasian mammals,^53,54^ with more recent efforts extending to African^55^ and Australian^56^ taxa. Progress has also been made in generating markers for nonmammalian groups, including birds,^57^ reptiles,^58^ amphibians,^59^ and fishes.^60^ Meanwhile, prospects for the application of peptide mass fingerprinting on the South American continent are relatively underexplored and are only represented by a few taxa.^61,62^

Here, we address this limitation by characterizing collagen peptide markers for eight extant and two extinct Xenarthran species, namely, *Dasypus novemcinctus*, *Zaedyus pichiy*, *Tolypeutes matacus*, *Priodontes maximus*, *Bradypus variegatus*, *Choloepus didactylus*, *Myrmecophaga tridactyla*, *Tamandua tetradactyla*, *Mylodon* sp.^†^, and *Glyptodon* sp.^†^. Candidate marker sequences were first identified through in silico digestion of previously published COL1 sequences and subsequently validated through a bottom-up proteomics approach, where tryptic digests were analyzed using mass spectrometry (LC-MS/MS). We report a unique set of collagen peptide markers that allow differentiation of Xenarthra from other related groups and within the Xenarthra order. This significantly amplifies the potential of ZooMS in the South American context in reconstructing past species abundance and distribution through time (Figure 1).

**Figure 1:**
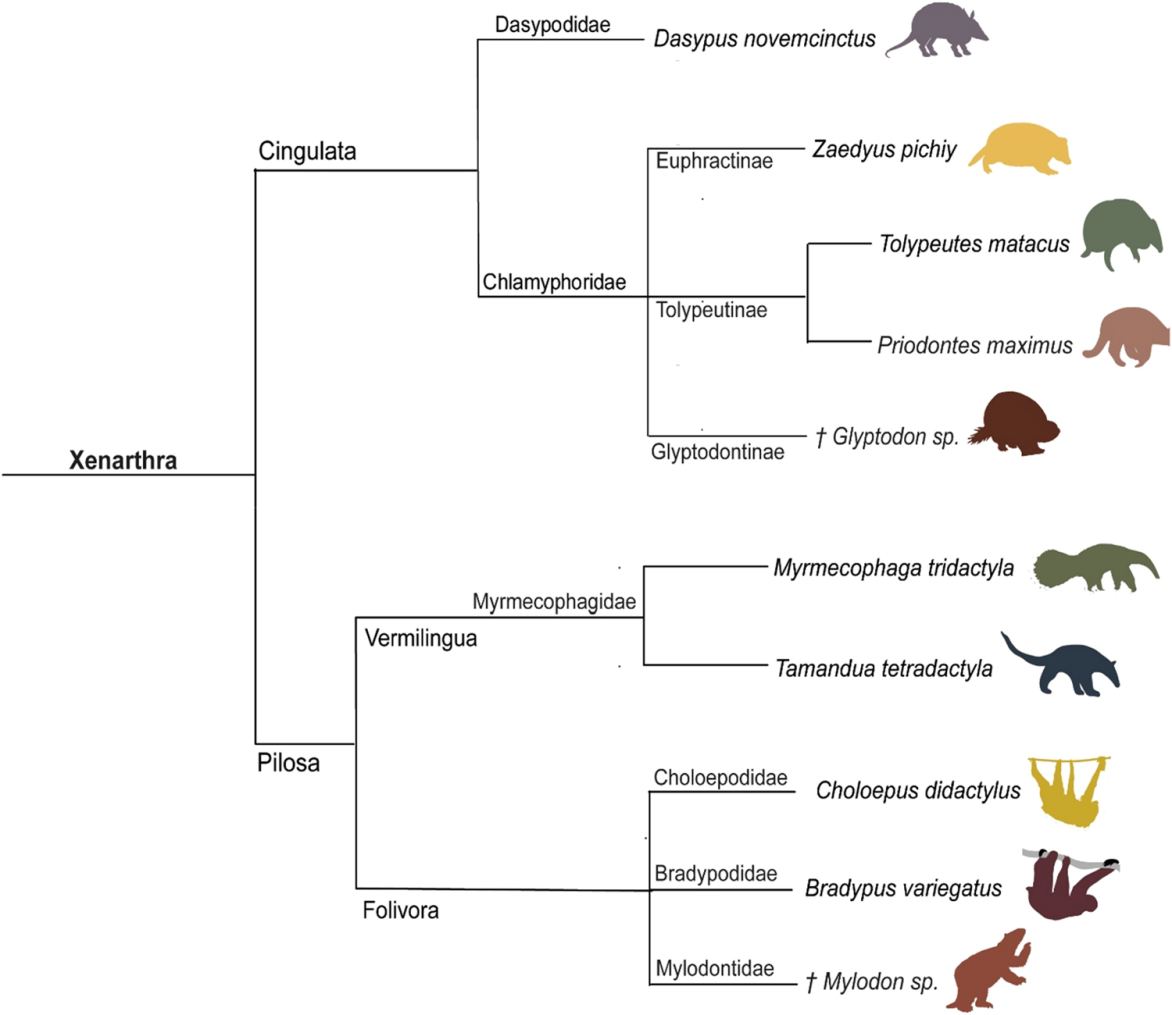
Phylogeny of extant and extinct South American Xenarthra and the species targeted for ZooMS marker development († Extinct taxa).

## Experimental Section

### Materials

Reference bone samples of extant sloths and armadillos were collected in duplicate from the Recent mammal collection at the Museum für Naturkunde, Leibniz Institute for Evolution and Biodiversity Science (MfN). Giant animal remains were collected from the Anthropological Museum of the Federal University of Goiás (FUG). Southern tamandua remains were collected from the Laboratory of Archeology, Curt Nimuendaju (ACN). Extinct *Mylodon* and *Glyptodon* remains were collected from the Zoological Museum of the University of Zurich (UZ). Fossil remains from the UZ collections of extinct Giant ground sloth taxa were labeled as *Mylodon listaei* and treated as *Mylodon* sp. in this study as the most secure taxonomic classification. Similarly, a recent study of extinct Glyptodonts in the UZ collections reassigns the PIMUZ A/V 438 specimen as *Neosclerocalyptus paskoensis*,^63^ a member of the Glyptodontinae^†^ subfamily. In particular, 25–40 mg of bone chips was collected from nondiagnostic sections of specimens. See the sample descriptions in Table 1.

**Table 1:** List of Taxonomic Reference Samples. *Samples Run on LC-MS/MS

lab ID	museum	museum ID	taxon	common name	weight
SAM-64*	MfN	ZMB_Mam_85937	*D. novemcinctus*	Nine-banded armadillo	31 mg
SAM-65	MfN	ZMB_Mam_36416	*D. novemcinctus*	Nine-banded armadillo	25 mg
SAM-70	MfN	ZMB_Mam_36080	*T. matacus*	Three-banded armadillo	28 mg
SAM-71*	MfN	ZMB_Mam_85923	*T. matacus*	Three-banded armadillo	26 mg
SAM-72	MfN	ZMB_Mam_3016	*P. maximus*	Giant armadillo	33 mg
SAM-73*	MfN	ZMB_Mam_37971	*P. maximus*	Giant armadillo	36 mg
SAM-80*	MfN	ZMB_Mam_38732	*Z. pichiy*	Pichi	27 mg
SAM-81	MfN	ZMB_Mam_45800	*Z. pichiy*	Pichi	36 mg
SAM-82*	MfN	ZMB_Mam_91347	*B. variegatus*	Brown-throated sloth	38 mg
SAM-83	MfN	ZMB_Mam_35824	*B. variegatus*	Brown-throated sloth	34 mg
SAM-84	MfN	ZMB_Mam_35825	*C. didactylus*	Linnaeus’s two-toed sloth	31 mg
SAM-85*	MfN	ZMB_Mam_102636	*C. didactylus*	Linnaeus’s two-toed sloth	29 mg
SAM-21*	FUG	NA	*M. tridactyla*	Giant anteater	38 mg
SAM-22	FUG	NA	*M. tridactyla*	Giant anteater	28 mg
SAM-23*	ACN	CN-70	*T. tetradactyla*	Southern tamandua	33 mg
SAM-24	ACN	NA	*T. tetradactyla*	Southern tamandua	34 mg
SAM-05*	UZ	PIMUZ A/V 438	*Glyptodon* sp.	Glyptodont	28 mg
SAM-06	UZ	PIMUZ A/V 438–124	*Glyptodon* sp.	Glyptodont	41 mg
SAM-01*	UZ	PIMUZ A/V 4635	*Mylodon* sp.	Giant Ground Sloth	39 mg
SAM-02	UZ	PIMUZ A/V 4297	*Mylodon* sp.	Giant Ground Sloth	35 mg

### Collagen Extraction

Collagen was extracted at the Max Planck Institute of Geoanthropology following previously published acid-insoluble^53,64^ and acid-soluble^53,65^ protocols. All samples were first extracted using an acid-insoluble method. If this method failed, then the acid-soluble method was used.

The bone fragments were demineralized in 500 μL of 0.6 M hydrochloric acid (HCl) for 64 h, after the acid supernatant was collected and stored at −20 °C for the acid-soluble method. The samples were rinsed 3 times with 200 μL of 50 mM AmBic buffer (ammonium bicarbonate NH_4_HCO_3_; pH 8), followed by an incubation for 1 h in 100 μL of AmBic at 65 °C. After gelatinization, the samples were digested with 1 μL of trypsin solution (0.4 μL/μg Thermo Scientific) for 20 h at 37 °C. After trypsin digestion, the reaction was terminated with 1 μL of TFA (trifluoroacetic acid, 5%). The generated peptides were purified using C18 ZipTips (Thermo Scientific). For the acid-soluble method, 250 μL of acid supernatant was transferred into 30 kDa ultrafilters and centrifuged at 3700 rpm until the acid had been pushed through the filter. The samples were washed twice with 500 μL of 50 mM AmBic. After 100 μL of 50 mM AmBic was added to the filter, the collagen was resuspended through pipetting. The AmBic was transferred from the filter into a clean centrifuge tube for tryptic digestion.

### MALDI-ToF (Matrix-Assisted Laser Desorption/Ionization–Tandem Time-of-Flight) Mass Spectrometry

All digests were mixed with matrix solution (10 mg of α-cyano-4-hydroxycinnamic acid in 7 mL of 85% acetonitrile/0.1% TFA) and spotted in triplicate on a 384-well Bruker MALDI ground steel target plate. The samples were run on a Bruker Autoflex Speed MALDI-ToF mass spectrometer (Bruker Daltonics) to produce spectra and fingerprints for taxonomic identification. A SNAP averaging algorithm was used to obtain monoisotopic masses (C: 4.9384, N: 1.3577, O: 1.4773, S: 0.0417, H: 7.7583). The screening was performed in the proteomics facility of the Max Planck Institute of Geoanthropology.

### LC-MS/MS (Liquid Chromatography with Tandem Mass Spectrometry)

After analysis of the MALDI data, one sample (20 μL) from each species was analyzed using LC-MS/MS to confirm the peptide marker sequences. Most of the samples were analyzed using LC-MS/MS at the Functional Genomics Center Zurich (FGCZ). *M. tridactyla* and *T. tetradactyla* were run at the Center for Biotechnology and Biomedicine (BBZ), University of Leipzig (see the list of samples in Table 1).

At FGCZ, mass spectrometry analysis was performed on an Orbitrap Exploris 480 mass spectrometer (Thermo Fisher Scientific) equipped with a Nanospray Flex Ion Source (Thermo Fisher Scientific) and coupled to an M-Class UPLC instrument (Waters). Solvent composition at the two channels was 0.1% formic acid for channel A and 0.1% formic acid, 99.9% acetonitrile for channel B. Column temperature was 50 °C. For each sample, 5 μL of peptides was loaded on a commercial nanoEase MZ Symmetry C18 Trap Column (100 Å, 5 μm, 180 μm × 20 mm, Waters) followed by a nanoEase MZ C18 HSS T3 Column (100 Å, 1.8 μm, 75 μm × 250 mm, Waters). The peptides were eluted at a flow rate of 300 nL/min. After a 3 min initial hold at 5% B, a gradient from 5% to 22% B in 40 min and 22% to 32% B in an additional 5 min was applied. The column was cleaned after the run by increasing to 95% B and holding 95% B for 10 min before re-establishing the loading condition for another 10 min. The mass spectrometer was operated in data-dependent mode (DDA) with a maximum cycle time of 3 s, funnel RF level at 40%, heated capillary temperature at 275 °C, and Advanced Peak Determination (APD) on. Full-scan MS spectra (350–1200 *m*/*z*) were acquired at a resolution of 120′000 at 200 *m*/*z* after accumulation to a target value of 300% or for a maximum injection time of 50 ms. Precursors with an intensity above 5′000 were selected for MS/MS. Ions were isolated using a quadrupole mass filter with a 1.2 *m*/*z* isolation window and fragmented by higher-energy collisional dissociation (HCD) using a normalized collision energy of 30%. HCD spectra were acquired at a resolution of 30,000, with a maximum injection time of 119 ms. The automatic gain control (AGC) was set to 100%. Charge state screening was enabled such that singly, unassigned, and charge states higher than seven were rejected. Precursor masses previously selected for MS/MS measurement were excluded from further selection for 20 s, and the exclusion window was set at 10 ppm. The samples were acquired using internal lock mass calibration on *m*/*z* 371.1012 and 445.1200.

At BBZ, the Zip-Tip cleaned sample peptide solution was diluted 1:20 with 3% (v/v) acetonitrile in water containing 0.1% (v/v) formic acid. Ten microliters of the diluted sample was injected onto an M-class UPLC system connected with a Synapt G2-Si ESI-IMS-QTOF mass spectrometer (Waters GmbH, Eschborn). Peptides were trapped with a Symmetry C18 column (5 μm, 180 μm × 20 mm, Waters GmbH, Eschborn) for 3 min with a flow rate of 10 μL/min at 1% eluent B. Eluent B was acetonitrile and eluent A was water, both containing 0.1% (v/v) formic acid. Trapped peptides were separated on a BEH130 C18 column (1.7 μm, 75 μm × 100 mm, Waters GmbH Eschborn) using a gradient from 3% to 60% B in 57 min and from 60% to 85% B in 3.5 min before re-equilibration of the column to 3% B. The mass spectrometer was operated with a NanoLockSpray dual ion source holding a fused silica emitter (20 μm ID × 6.3 cm L × 365 μm OD, 10 μm Polished end, MS Wil BV, Aarle-Rixtel) at 3 kV, source temperature of 100 °C, sampling cone of 30 V, source offset of 80 V, cone gas flow of 20 L/h, nanoflow gas pressure of 0.2 bar, and purge gas of 600 mL/h. For lockmass correction, a 100 fmol/μL GluFib solution in 50% B was infused at 0.5 μL/min to a taper tip emitter (186003932, Waters GmbH, Eschborn) at 3 kV. For recording, a HD-DDA method was created with MassLynx 4.2 SCN 983 including the following parameters: Acquisition was done in positive resolution mode; for MS survey, mass range was from 50 to 5000 Da and scan rate was 0.3 s if intensity of the individual ion rose above 1000 acquisition was switched to MS/MS to record the top six ions at a scan rate of 0.3 s until a TIC threshold of 1,00,000 was reached or 0.5 s has elapsed. Only doubly, triply, or quadruply charged ions were considered for MS/MS; masses between 50 and 350 *m*/*z* were excluded, and real-time exclusion of 10 s was used. MS/MS fragmentation was done in the trap cell using a collision energy ramp with LM 12–18 V and HM 50–70 V. Wideband Enhancement was used for mobility separation of the fragment ions before detection. The Wideband *.lue* file was created with Drift Scope 2.9 (Waters GmbH, Eschborn).

### Marker Identification and Confirmation

For all extant taxa with publicly accessible annotated genomes, collagen type I protein sequences were obtained from the corresponding protein translations.^66–79^ In cases where genome assemblies were of low quality, either the COL1A1 or COL1A2 gene contained less than 50% of its coding sequence. Under these circumstances, only COL1 chains with at least 50% of the coding sequence and no gene-inactivating mutations were retained for theoretical digestion. For sequences that did not meet these criteria, in silico digestion was not performed, and peptide identification relied exclusively on the LC-MS/MS data generated in this study. The COL1 sequences of the extinct species *Mylodon sp.* (C0HJP3, C0HJP4), generated through proteomic shotgun sequencing, were obtained from UniProt.^80^ Collagen type I protein sequences of *D. novemcinctus* (XP_058139988.1, XP_004470764.1) and *C. didactylus* (XP_037664782.1, XP_037692619.1), available in the NCBI RefSeq database, were used as references to assess the sequence validity of other species through orthologous alignment.^70,81–83^ The aligned protein sequences were trimmed to remove signal peptide regions, ensuring that only the mature collagen sequences were analyzed. These mature sequences were then subjected to in silico tryptic digestion using the ExPASy PeptideMass tool.^84,85^ Digestion was performed with trypsin, allowing up to three missed cleavages. Peptides were restricted to a mass range of 800–4000 Da, with a charge state of [M + H]^+^ and monoisotopic mass specified (Figure 2).

**Figure 2:**
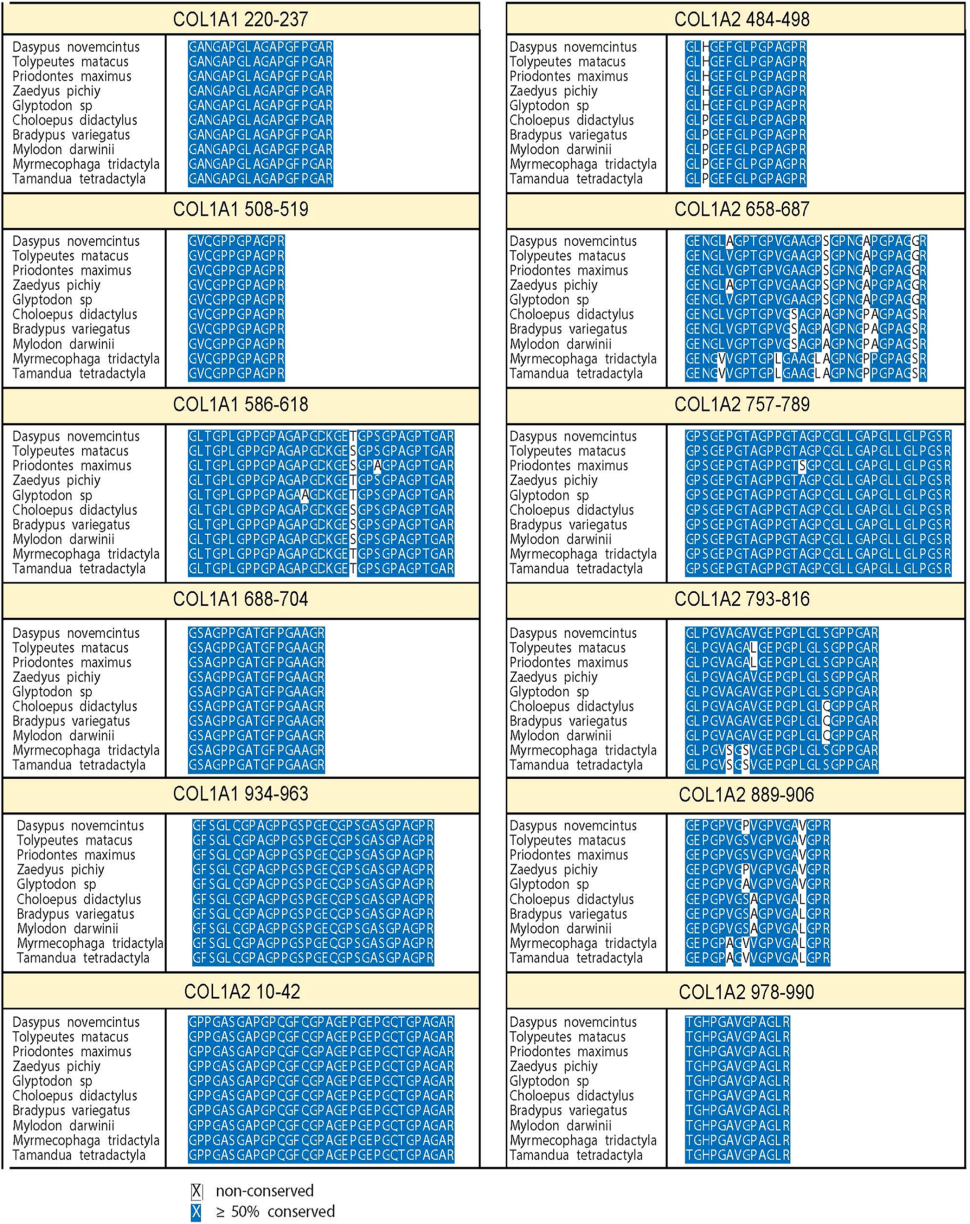
Standard peptide marker sequences named according to the convention proposed by Brown et al., 2021.^86^ Conserved regions are shown as white text on a blue background, while sequence variations are indicated by black text on a white background.

MALDI spectra acquired in Bruker Flex format were converted to *mzML* file format using the MALDIQuant and MALDIQuantForeign packages in R.^87,88^ The resulting *mzML* files were analyzed with the mMass software.^89^ Before peak identification, spectra were preprocessed in mMass by applying baseline correction with a precision of 15 and a relative offset of 25, followed by smoothing using the Savitzky–Golay method with a window size of 0.3 *m*/*z* and two smoothing cycles. Monoisotopic peak lists were generated by peak picking, employing a signal-to-noise threshold of 3.5, and the spectra were subsequently deisotoped. Each spectrum was then evaluated visually for the presence of 12 standard marker peaks, and a presence/absence matrix was generated. The registered collagen fingerprints for each specimen are listed in Table 2.

**Table 2:** Identified ZooMS Markers. * Not Visible on MALDI, Recorded through LC-MS/MS; **Visible on MALDI, Not Via LC-MS/MS

	Cingulata					Pilosa				
	*D. novemcinctus*	*Z. pichiy*	*T. matacus*	*P. maximus*	*Glyptodon*	*B. variegatus*	*C. didactylus*	*Mylodon*	*M. tridactyla*	*T. tetradactyla*
COL1α1 508–519	1105.6	1105.6	1105.6	1105.6	1105.6	1105.6	1105.6	1105.6	1105.6	1105.6
COL1α1 688–704	1459.7	1459.7	1459.7	1459.7	1459.7	1459.7	1459.7	1459.7	1459.7	1459.7
COL1α1 220–237	1585.7	1585.7	1585.7	1585.7	1585.7	1585.7	1585.7	1585.7	1585.7	1585.7
COL1α1 934–963	2662.2/2647.2	2662.2/2647.3	2662.2/2647.4	2662.2/2647.5	2662.2/2647.6	2662.2/2647.7	2662.2/2647.8	2662.2/2647.9	2662.2/2647.10	2662.2/2647.11
COL1α1 586–618	2850.6	2850.6	2850.6	2853.4	-	2868.4	2868.4	2868.4	2883.4	2883.4
COL1α1 586–618	2866.6	2866.6	2866.6	2869.4	2914.5	2884.4	2884.4	0	2899.4	2899.4
COL1α2 978–990	1189.6	1189.6	1189.6	1189.6	1189.6	1189.6	1189.6	1189.6	1189.6*	1189.6*
COL1α2 978–990	1205.6	1205.6	1205.6	1205.6	1205.6	1205.6	1205.6	1205.6	1205.6	1205.6
COL1α2 484–498	1477.7	1477.7	1477.7	1477.7	1477.7	1453.7	1453.7	1453.7	1453.7	1453.7
COL1α2 793–816	2131.1	2131.1	2129.1	2131.1	2131.1	2172.2	2172.2	2172.2	2147.2	2147.2
COL1α2 658–687	2496.2	2496.2	2541.2	2524.2	2542.2	2554.2	2554.2	2554.2	2597.2	2597.2**
COL1α2 889–906	1598.7	1598.9	1588.7	1588.8*	1604.8	1602.9*	1574.6	-	1586.7	1586.7
COL1α2 757–789	2940.5	2940.5	2940.5*	2940.5	2940.5	2940.5*	2940.5*	-	2940.5	2940.5*
COL1α2 757–789	2956.5	2956.5	2956.5*	2956.5	2956.5	2956.5	2956.5	2956.5	2956.5	2956.5
COL1α2 10–42	2975.3	2975.3	2975.3	2975.3	2975.3	2975.3	2975.3	2975.3	2975.3	2975.3*

The raw LC-MS/MS files were converted to *mgf* (Mascot Generic Format) using the MSConvert utility (Proteowizard) and subsequently analyzed with Mascot (v2.7.0) to confirm the sequences of collagen peptide biomarkers.^90,91^ For each of the three taxonomic groups studied, armadillos, sloths, and anteaters, a representative model organism was selected: *D. novemcinctus*, *C. didactylus*, and *T. tetradactyla*, respectively. A FASTA database comprising mammalian COL1 orthologs was generated using the NCBI Data sets command-line tool and supplemented with the proteome of each group’s model organism, sourced from UniProtKB, to create a custom database per taxon (see Supporting File). Mascot searches were conducted with trypsin specified as the proteolytic enzyme, permitting up to three missed cleavages. Peak labeling was set to monoisotopic, and peptide charges were set to 2+ and 3+. No fixed modifications were specified, while oxidation of proline (P) was included as the only variable modification. Error-tolerant search mode was enabled. Instrument-specific search parameters were used: for the data from the Orbitrap Exploris 480 mass spectrometer, the peptide mass tolerance was set to 10 ppm and the MS/MS tolerance to ± 0.02 Da; for the BBZ data set, tolerances were 20 ppm and ± 0.06 Da, respectively. Mascot search results were exported as *CSV* files and processed using a custom in-house R script to match experimentally detected shotgun peptides with those predicted from in silico tryptic digestion, enabling confirmation of peptide presence across species. Identified peptide markers and corresponding sequences are listed in Supporting Table S1.

## Results

All of the specimens analyzed yielded good-quality MALDI-ToF and MS/MS spectra that were suitable for the identification of collagen type I markers. Collagen (I) was the main protein identified in all of the samples. The presence of 12 standard collagen markers was confirmed in all specimens, except for *T. tetradactyla* (BBZ), whose COL1A2 658–687 sequence could not be confirmed through MS/MS, and the COL1A2 10–42 marker was not observed in the MALDI-ToF spectra. The presence–absence matrix of the 12 standard collagen markers observed in MALDI-ToF spectra is provided in Table 2. Examples of peptide mass fingerprints for Pilosa are displayed in Figure 3 and those for Cingulata in Figure 4.

**Figure 3:**
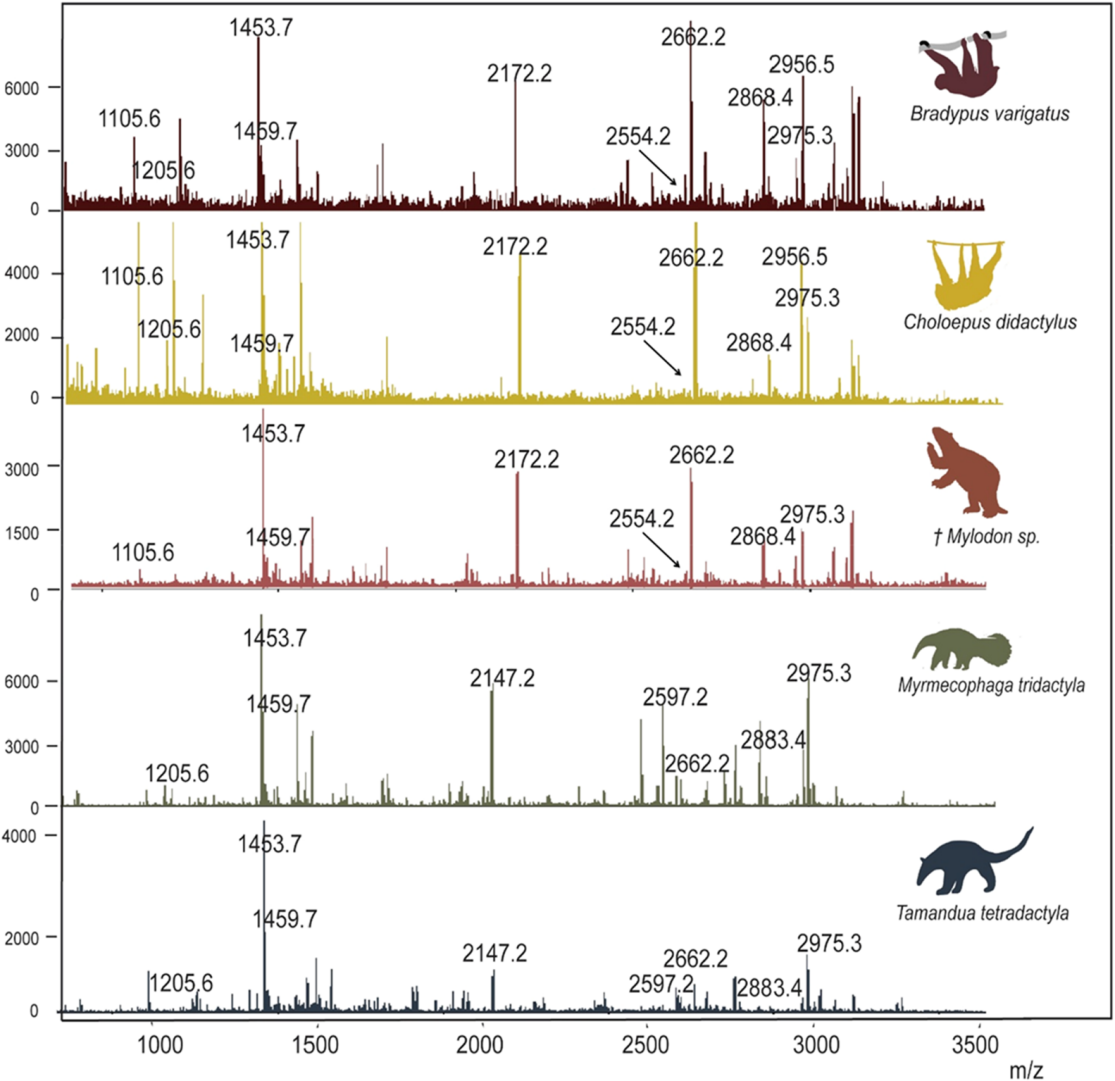
Example MALDI-ToF MS spectra of the analyzed Pilosa.

**Figure 4:**
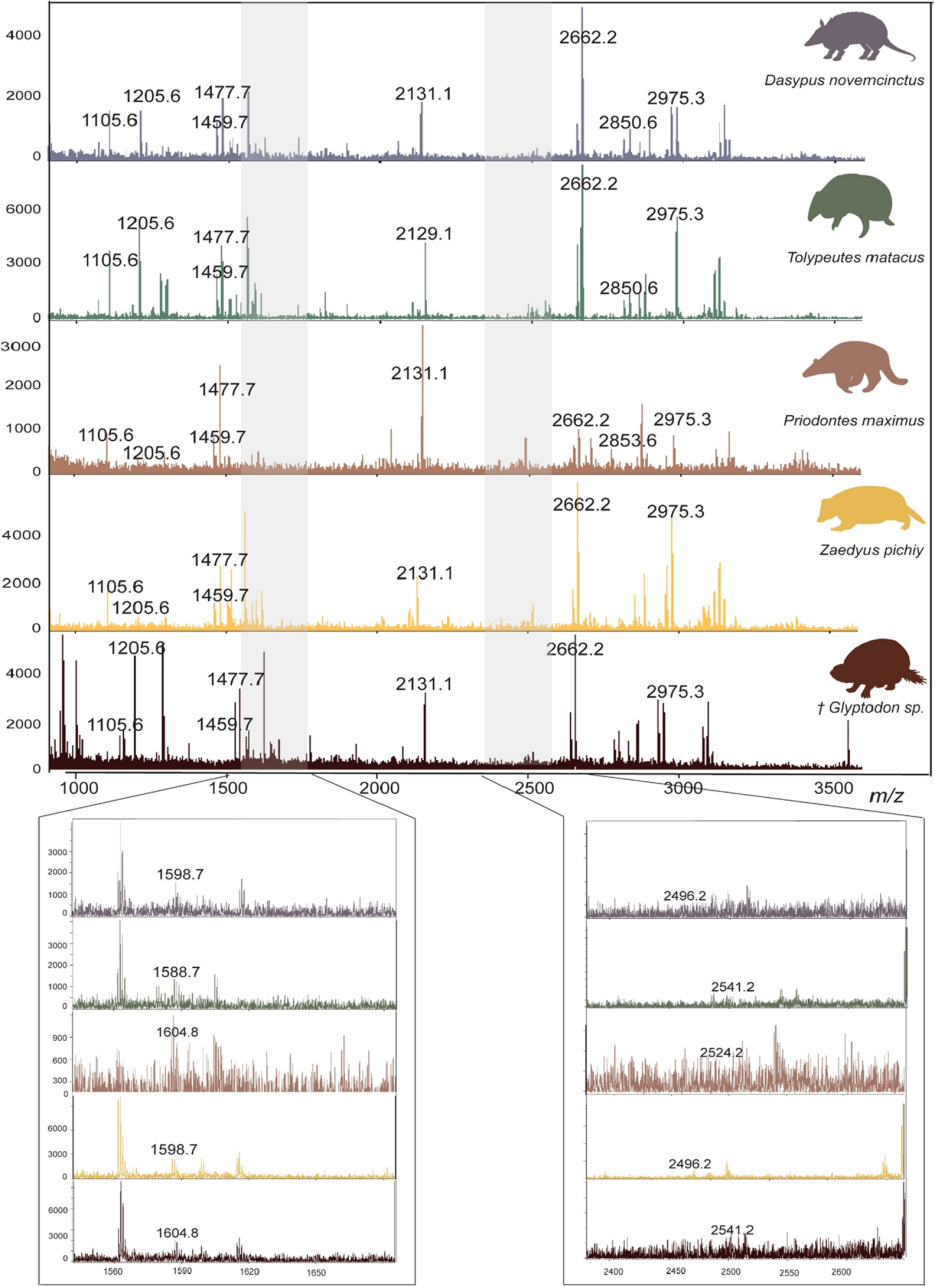
Example MALDI-ToF MS spectra of the analyzed Cingulata.

Our data show that collagen type I (COL1) peptide markers generally allow for the differentiation between the Xenarthran superorders Cingulata and Pilosa. Although some peptide markers are shared within Pilosa, the two suborders Vermilingua (anteaters) and Folivora (sloths) possess characteristic collagen peptide variants. Across the three groups, most peptide markers of the COL1A1 chain are conserved, while COL1A2 peptide markers exhibit more MALDI-accessible sequence variation. The highly conserved COL1A1 508–519 (P1) peptide marker is observed in all species at *m*/*z* 1105.6, consistent with other reported terrestrial mammals.^59^ Additional conserved markers include COL1A1 688–704 (*m*/*z* 1459.7), COL1A1 220–237 (*m*/*z* 1585.7), COL1A1 934–963 (*m*/*z* 2647.2), and its oxidized forms (*m*/*z* 2662.3), COL1A2 978–990 (A) at *m*/*z* 1189.6 and *m*/*z* 1205.6 (oxidized) and COL1A2 10–42 at *m*/*z* 2975.3 (Table 2).

The COL1A2 484–498 marker is the first low-mass peptide to exhibit sequence variation that distinguishes Cingulata and Pilosa. In Cingulata, this peptide is observed at *m*/*z* 1477.7 and contains a histidine residue at the third position from the N-terminus. In contrast, the Pilosa variant shows a proline at the same position (Figure 2), with the peptide peak detected at *m*/*z* 1453.7. The observed 24 Da mass difference, rather than the expected ∼40 Da from a histidine-to-proline substitution, is explained by differences in post-translational modifications: in the Cingulata variant, the proline at position nine undergoes oxidation, while in the Pilosa variant, both the third- and ninth-position prolines are oxidized. The COL1A2 889–906 marker further distinguishes among armadillos, sloths, and anteaters. In *D. novemcinctus* and *Z. pichiy*, the sequence is identical and observed at *m*/*z* 1614.9 (single oxidation). In *Tolypeutinae armadillos* (*T. matacus* and *P. maximus*), a serine (instead of proline) at position 8 (Figure 2) results in a marker at *m*/*z* 1604.8 (single oxidation). *Glyptodon* sp. is distinct with an alanine at position 8, producing peaks at *m*/*z* 1588.8 (single proline oxidation) and 1604.8 (two proline oxidations). Due to an isobaric overlap at *m*/*z* 1604.8 between the double-oxidized *Glyptodon* variant and the single-oxidized Tolypeutinae variant, attribution of this peak to *Glyptodon* should only be made if the nonoxidized peak at *m*/*z* 1572.8 or the single-oxidized form is also present. All studied sloth species share the same COL1A2 889–906 variant, with alanine at position 9 and leucine at position 15 instead of valine, appearing at *m*/*z* 1590.9 (single oxidation) and occasionally at *m*/*z* 1574.9 (nonoxidized). The anteater variant (Figure 2) has alanine at position 6 (replacing valine), valine at position 8 (replacing proline), and leucine at position 15 (replacing valine), producing peaks at *m*/*z* 1586.9 and *m*/*z* 1602.9 (single oxidation).

The peptide marker COL1A2 793–816 effectively distinguishes the three major xenarthran groups and also differentiates the Tolypeutinae subfamily within armadillos. In most armadillo species, this marker appears at *m*/*z* = 2131.1, corresponding to a variant with three proline oxidations. In contrast, members of Tolypeutinae exhibit a leucine substitution (replacing valine) at position 9 (Figure 2), producing peaks at *m*/*z* 2129.1 (two oxidations) and *m*/*z* 2145.1 (three oxidations), with the latter being more prominent in the MALDI and MS/MS spectra. Sloths show a glutamine substitution (in place of serine) at position 18 (Figure 2), resulting in a peak at *m*/*z* 2172.1 (three oxidations and no deamidation). However, MS/MS data confirm partial deamidation of the substituted glutamine in some spectra, warranting the inclusion of both *m*/*z* 2172.1 and *m*/*z* 2173.1 in the characterization of this marker. Anteaters possess serine substitutions at positions 6 and 8 (replacing alanines), and the marker is observed at *m*/*z* 2147.1 (two oxidations). The COL1A2 658–687 marker is observed at *m*/*z* 2497.2 in *D. novemcinctus* and *Z. pichiy*, with an oxidized variant detected at *m*/*z* 2513.2. In Tolypeutinae and *Glyptodon* sp., it appears at *m*/*z* 2542.2 (singly oxidized and deamidated), with a valine at position 6 instead of the alanine (Figure 2) found in *D. novemcinctus* and *Z. pichiy*. All sloth species display identical multiple amino acid substitutions in this marker, relative to the *D. novemcinctus* variant (Figure 2). Sloths exhibit multiple substitutions: a valine at position 6 (shared with Tolypeutinae), serine at position 14 (replacing alanine), alanine at position 18 (replacing serine), an inversion of residues at positions 23–24 (AP → PA), and serine at position 29 (replacing glycine). This sloth-specific variant appears in its nonoxidized and nondeamidated form at *m*/*z* 2555.3.

The COL1A1 586–618 marker distinguishes three main taxonomic groups based on residue 21: species with a serine at this position, species with a threonine, and species exhibiting either serine or threonine along with additional amino acid substitutions. All the studied sloths and *T. matacus* have an identical COL1A1 586–618 marker at *m*/*z* 2869.4 (two proline oxidations) and *m*/*z* 2885.4 (three proline oxidations) with a serine at position 21, while the anteaters, *D. novemcinctus*, and *Z. pichiy*, have a threonine at position 21 (Figure 2), and the marker is observed at *m*/*z* 2867.4 (two proline oxidations) and *m*/*z* 2883.4 (three proline oxidations). The COL1A1 586–618 marker of armadillos has a threonine at position 21 and is observed at *m*/*z* 2883.4 (two proline oxidations) and *m*/*z* 2899.4 (three proline oxidations) in the MALDI spectra. *P. maximus* has a serine at position 21 and an alanine instead of a serine at position 24, with peaks at *m*/*z* 2853.4 (two proline oxidations) and *m*/*z* 2869.4 (three proline oxidations), while *Glyptodon* sp. features a threonine at position 21 and an alanine at position 15 (replacing proline), with only the double-oxidized peak at *m*/*z* 2914.5 observable in the MALDI spectra.

## Discussion

The COL1 peptide markers analyzed in this study confirm the current phylogenetic framework of Xenarthra, distinguishing armadillos, sloths, and anteaters. The consistent sequence differences in COL1A2 484–498 marker (Figure 2) reflect the deep divergence of the basal split between Cingulata and Pilosa. Within Cingulata, several COL1 markers illuminate the subgroup of armadillos. The COL1A2 889–906, COL1A2 793–816, and COL1A1 586–618 markers are especially effective in separating Dasypodinae (all species within the *Dasypus* genus) and Tolypeutinae. Despite being a member of the subfamily Euphractinae, *Z. pichiy* has an identical panel of COL1 marker sequences compared to those of *D. novemcinctus*, reflecting their close phylogenetic relation^92–94^ and the inherent limitations of COL1 sequences for reconstruction of evolutionary lineages, in line with current consensus.^95–97^ *Glyptodon* sp. is readily distinguished from extant armadillos with several unique COL1 marker sequence variations. However, within Cingulata, often *Glyptodon* sp. marker sequences are identical with the species of Tolypeutinae subfamily, indicative of a closer evolutionary relation compared to *D. novemcinctus* and *Z. pichiy* (Figure 2). This fits with genetic studies, which have placed glyptodonts as a monophyletic clade with the Chlamyphorinae, Tolypeutinae, and Euphractinae subfamilies in Chlamyphoridae.^93,94,98,99^

Among sloths, shared peptide profiles between *Choloepus* (a mylodontoid) and *Bradypus* (a megatherioid) confirm their common membership in Folivora, while also illustrating a high degree of peptide conservation in COL1.^80^ The lack of peptide divergence between these two genera likely reflects the relatively slow evolutionary rate of collagen but does not contradict their placement in distinct higher-order sloth clades. The two anteater species, *M. tridactyla* and *T. tetradactyla*, belong to the same family of Myrmecophagidae, and their close monophyly is represented in their identical COL1 peptide marker profile, which is, however, distinctive from sloths and armadillos (Figure 2). As a result, overall, apart from the major divisions of Cingulata and Pilosa, the COL1 marker sequences also reflect the finer-scale relations within these groups, consistent with current genomic and morphological interpretations.

The development of a ZooMS peptide marker reference library for South American Xenarthrans marks a significant advancement for both zooarcheological and paleontological research on the continent. This enhanced resolution holds considerable potential for advancing our understanding of human–animal interactions and biodiversity change through time. It is well documented that giant anteaters and giant armadillos are considered high-ranking prey in the optimal foraging theory in eastern South America.^100–104^ Additionally, sloths and armadillos are frequently recovered in paleontological and archeological sites all across the South American Lowlands in Bolivia, Brazil, Colombia, Argentina, etc.^13,43–47^ However, because the vast majority of Xenarthran remains recovered from archeological sites are osteoderms, precise taxonomic identification at the genus or species level is often difficult. In this context, ZooMS offers a valuable solution by enabling the molecular identification of these morphologically ambiguous elements, thereby unlocking previously inaccessible data on extinction patterns and past species distributions. Furthermore, the vast majority of South American Holocene sites lack dedicated zooarcheological studies, limiting our ability to assess the nature of human–Xenarthran interactions.^105^ As a result, systematic evaluations of their roles in Holocene societies remain rare,^106^ despite their widespread occurrence in archeological contexts^14,46,48,49^ and possible representations in ancient rock art.^107,108^ Application of ZooMS to Holocene contexts can offer a new avenue for investigating the roles Xenarthran mammals played in the daily lives of human societies and trace whether the exploitation of Xenarthrans varied regionally or temporally in response to environmental or cultural changes.

Today, many extant Xenarthran species face precarious population declines and are increasingly recognized as priority taxa for conservation research due to their threatened status.^3,7,21,109^ According to the latest global assessment by the IUCN Red List of Threatened Species, 12 of the 38 recognized species are listed as Least Concern, the remaining are Near Threatened (four), Vulnerable (four), Critically Endangered (one), and Data Deficient (five), or their extinction risk has not yet been (re)­evaluated in light of recent taxonomic changes (12). Xenarthrans face multiple threats across their range, with habitat loss from expanding agriculture, logging, and urbanization being among the most significant. Invasive species, such as domestic dogs, also pose substantial risks through competition and predation. Additionally, many Xenarthrans are being heavily hunted for food, kept as pets, or exploited for medicinal and religious purposes.^3,21^ While modern threats such as habitat destruction, invasive species, and overexploitation continue to drive population declines, efforts to protect and restore these taxa are hindered by major gaps in long-term demographic and distributional data. For most Xenarthran species, natural history data remain scarce and restricted to specific biomes, offering an incomplete picture of how these species respond to varying environmental conditions.^3,110–112^ In this context, detailed zooarcheological records can offer unparalleled potential for reconstructing their past distribution and examining long-term biodiversity trends, thus providing insights for ecological restoration and forming present-day conservation strategies.

While this research represents the first attempt to develop comprehensive ZooMS reference data for a broad range of South American endemic species, significant gaps remain. Many native taxa, such as rodents, opossums, and other marsupials, still lack peptide marker references, limiting our ability to identify their remains in archeological contexts. Expanding ZooMS reference libraries to include these under-represented groups is essential for improving taxonomic resolution in zooarcheological studies. Such efforts will not only enhance our understanding of past faunal diversity and human–animal interactions but also support more informed conservation strategies by providing long-term ecological baselines across the continent. Precise taxonomic identification of faunal remains in archeological contexts is crucial for reconstructing past biodiversity and assessing its responses to climatic and cultural shifts over time. The reference peptide markers developed in this study enable species-level identification of South American Xenarthrans through ZooMS, marking a critical advancement in applying paleoproteomic methods to regional zooarcheological and palaeontological research. This expanded taxonomic resolution enhances the capacity to investigate the abundance, distribution, and population dynamics of Xenarthrans and offers important perspectives on the long-term factors contributing to their present-day conservation status.

## Supplementary Material





## Data Availability

All ZooMS spectra for identified samples and fasta files with translated collagen sequences are available on Mendeley Data open access repository doi: 10.17632/wvyw2yn4 ms.1. LC-MS/MS data files are available at ProteomExchange record PXD065697 and were uploaded through MassIVE (MSV000098409). The files can be accessed with an FTP client using the download link: ftp://MSV000098409@massive-ftp.ucsd.edu

## Notes

Identified peptide markers and corresponding sequences (XLSX)
MASCOT Error-tolerant Marker sequences by species (PDF)
