# Supplementary material for: Peptide Mass Fingerprinting of South American Xenarthrans: A New Resource for Zooarcheology and Palaeontology
Source: J Proteome Res. 2025 Nov 3;24(12):6101–14. doi: 10.1021/acs.jproteome.5c00636 (PMC12687363; doi:10.1021/acs.jproteome.5c00636)

Peptide Mass Fingerprinting of South American Xenarthrans: A New Resource for Zooarcheology and Palaeontology

Mariya Antonosyan[1]*, Roshan Paladugu[2]*, Michael Ziegler[1], Gabriela Prestes Carneiro[3], Eliane Chim[4], Andre Menezes Strauss[4], Diego Mendes[5], Rafael Lemos[6], Jorge Domingo Carrillo-Briceño[6], Stefanie Schirmer[8], Jana Ilgner[7], Daniela Volke[8], Patrick Roberts[1]

1. *Department of Coevolution of Land Use and Urbanisation, Max Planck Institute of Geoanthropology, 07745, Jena, Germany*
2. *Department of Evolutionary Genetics, Max Planck Institute for Evolutionary Anthropology, 04103, Leipzig, Germany*
3. *National Museum of Natural History, 75005, Paris, France*
4. *Museum of Archeology and Ethnology, University of São Paulo, 05508-070, São Paulo, Brazil*
5. *Anthropological Museum of the Federal University of Goiás, 74605-010, Goiânia, Brazil*
6. *Paleontological Institute, University of Zurich, 8006, Zurich, Switzerland*
7. *Laboratory Central Unit, Max Planck Institute of Geoanthropology, 07745, Jena, Germany*
8. *Center for Biotechnology and Biomedicine (BBZ), University of Leipzig, 04103, Leipzig, Germany*

Supporting information

MASCOT Error-tolerant Marker Sequences by Species

Contents

Bradypus variegatus 2
Choloepus didactylus 6
Dasypus novemcinctus 11
Glyptodon sp. 15
Mylodon sp. 20
Myrmecophaga tridactyla 24
Priodontes maximus 28
Tamandua tetradactyla 33
Tolypeutes matacus 37
Zaedyus pichiy 41

Bradypus variegatus

COL1A1 508 - 519

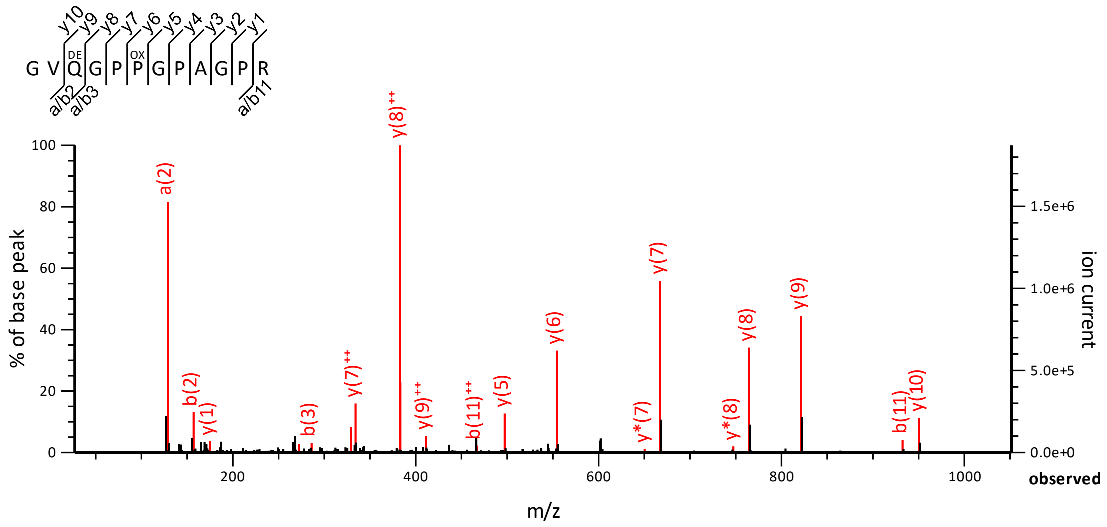

COL1A1 688 - 704

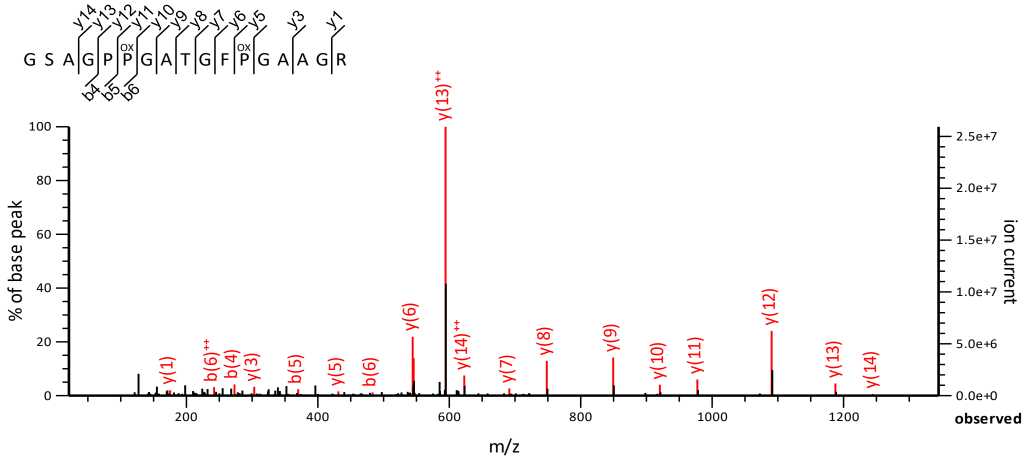

COL1A1 220 - 237

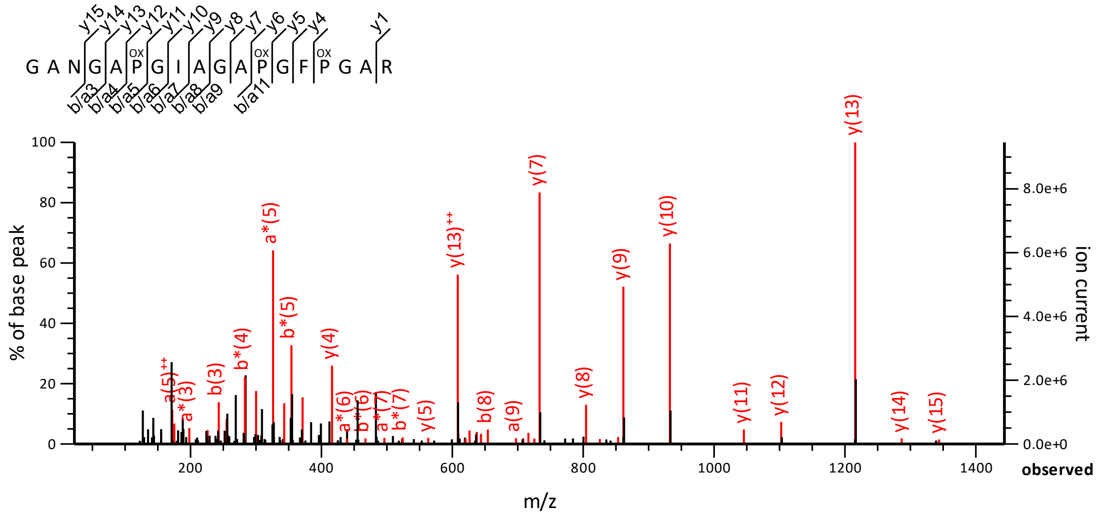

COL1A1 934 - 963

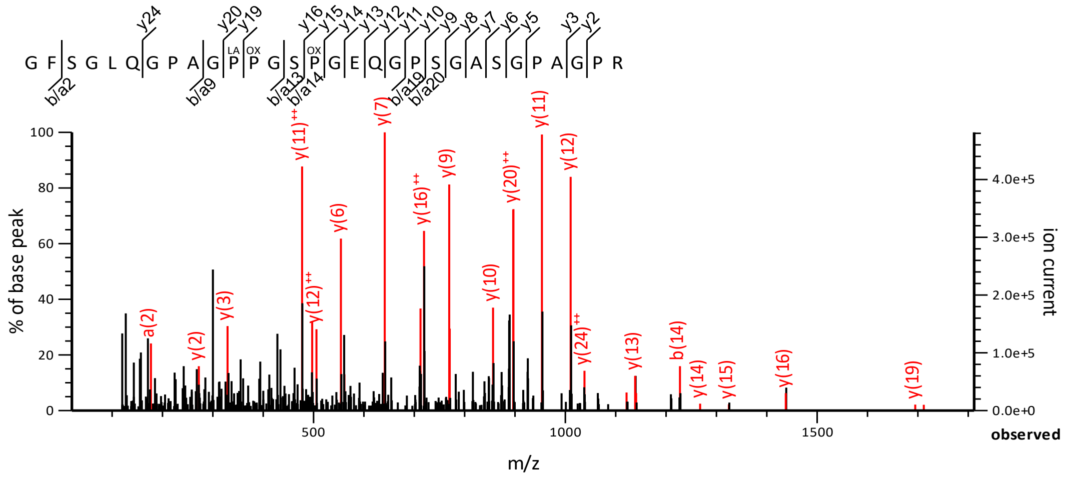

COL1A1 586 - 618

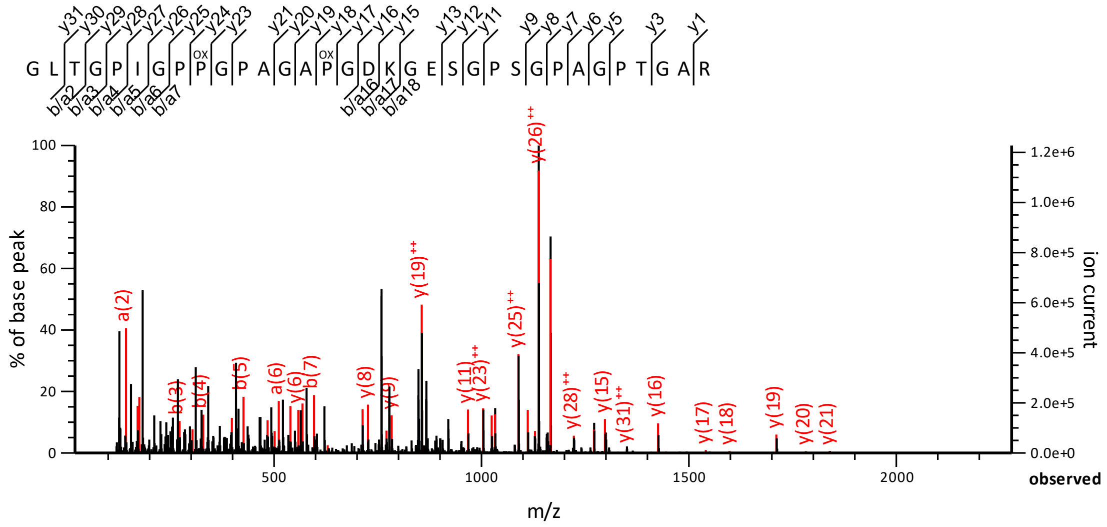

COL1A2 978 - 990

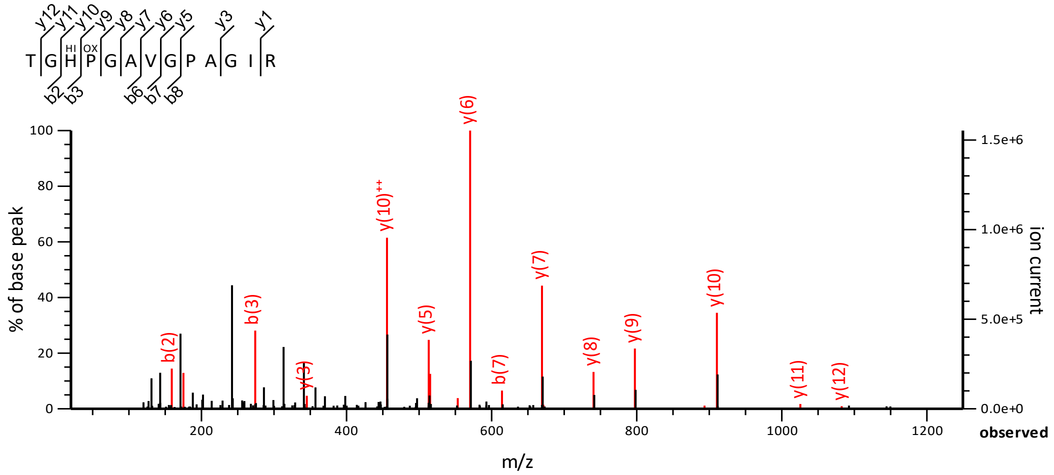

COL1A2 484 - 498

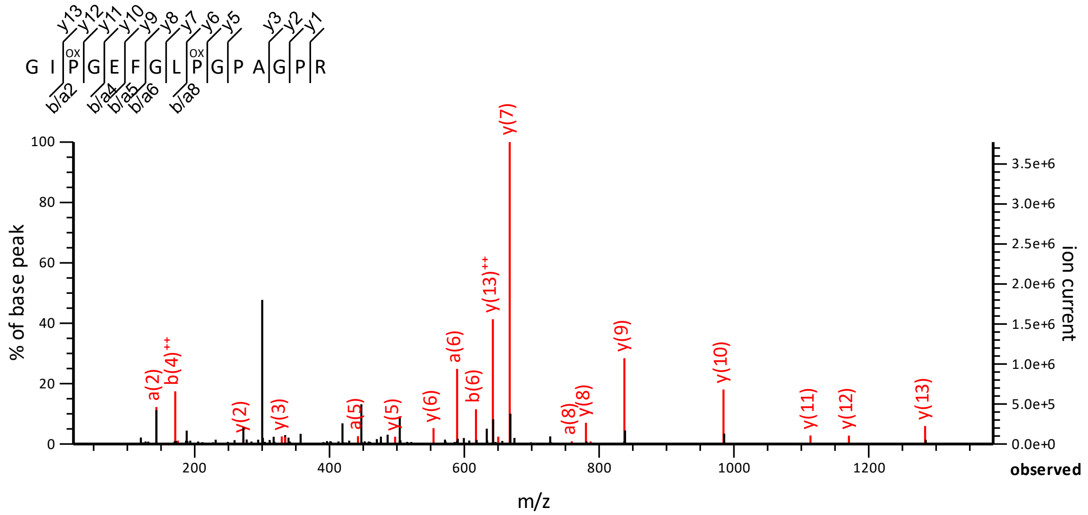

COL1A2 889 - 906

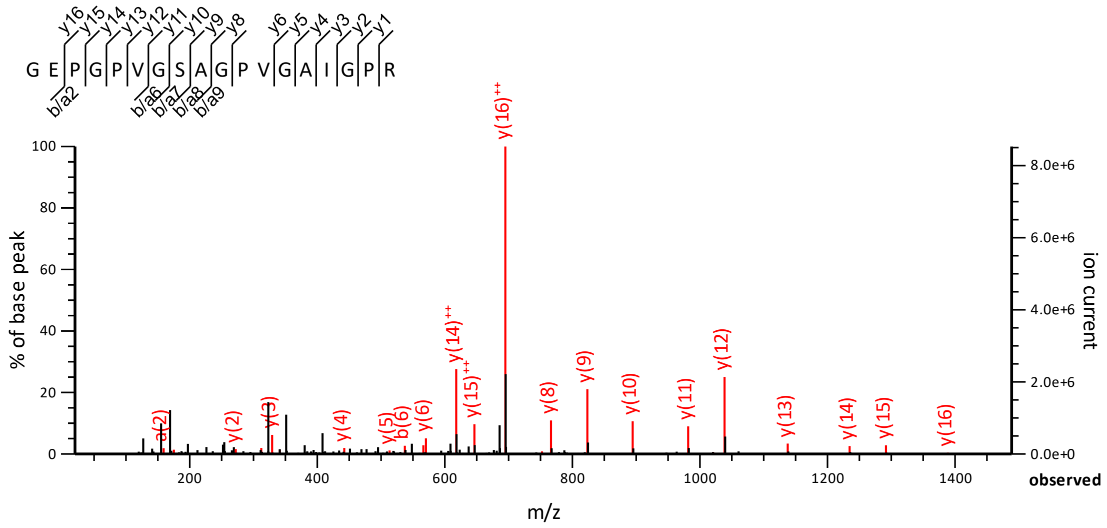

COL1A2 793 - 816

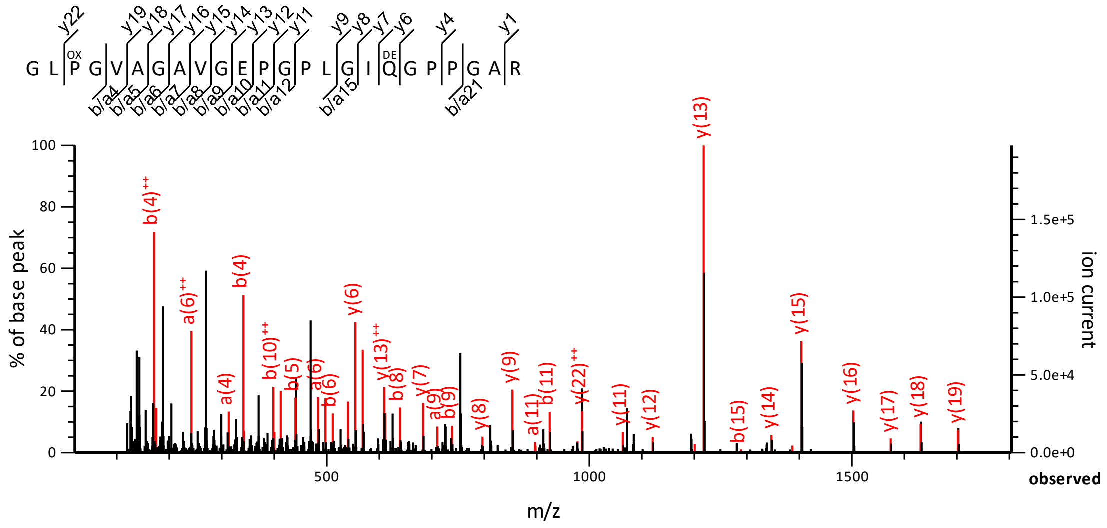

COL1A2 658 - 687

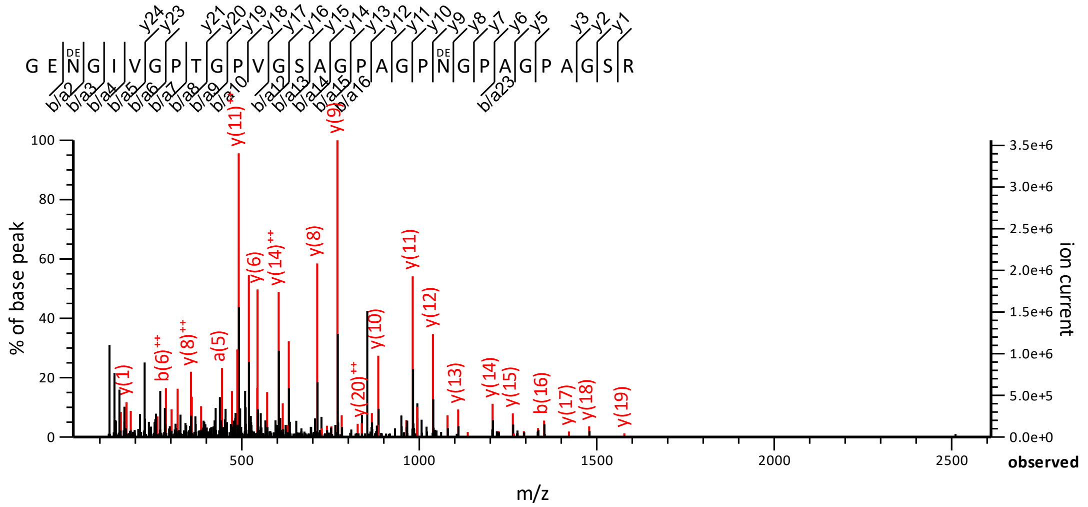

COL1A2 757 - 789

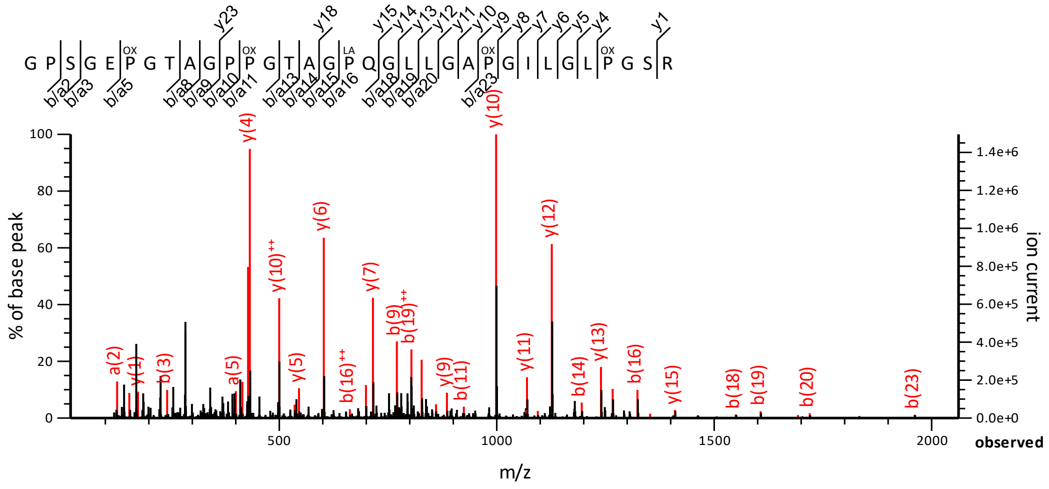

COL1A2 10-42

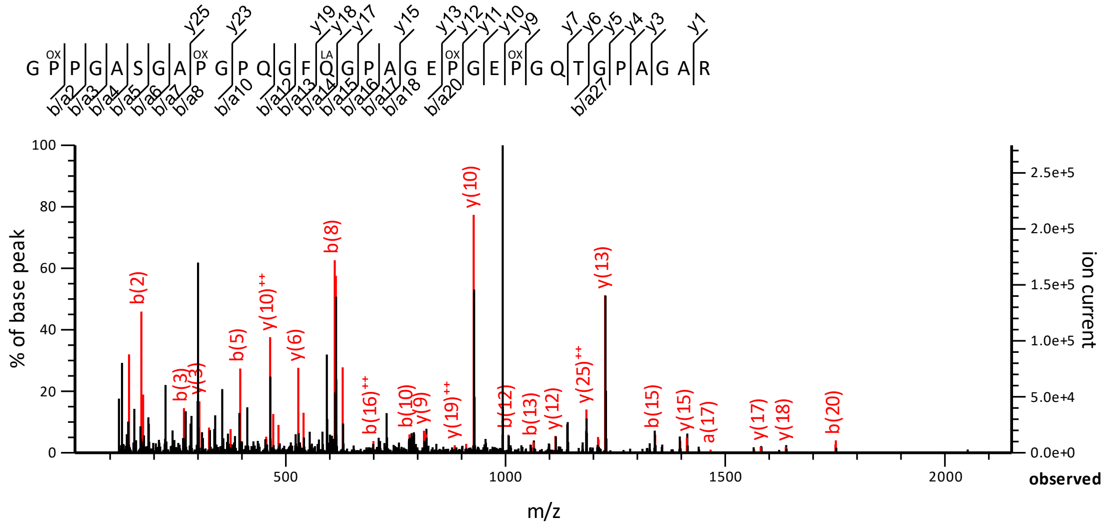

Choloepus didactylus

COL1A1 508 - 519

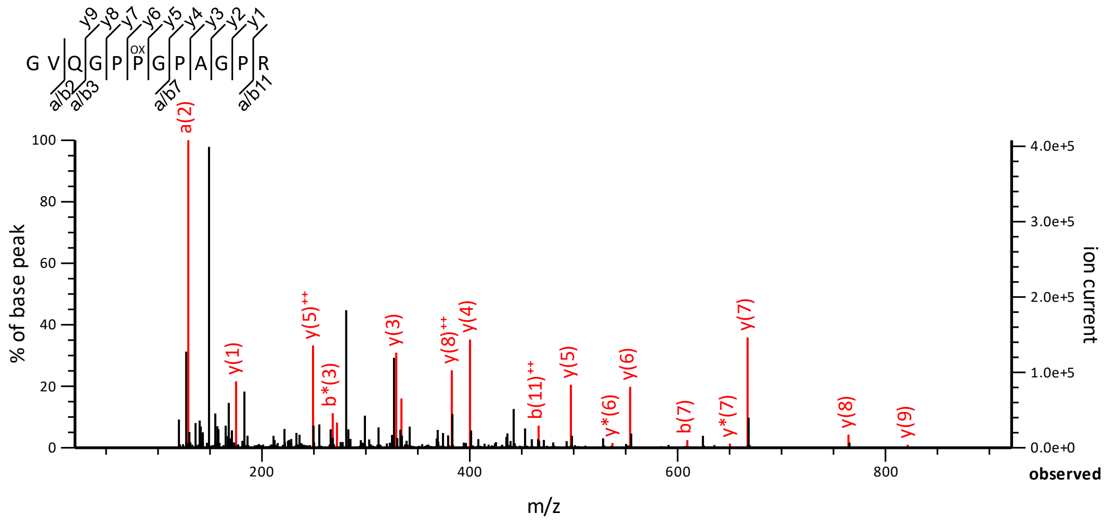

COL1A1 688 - 704

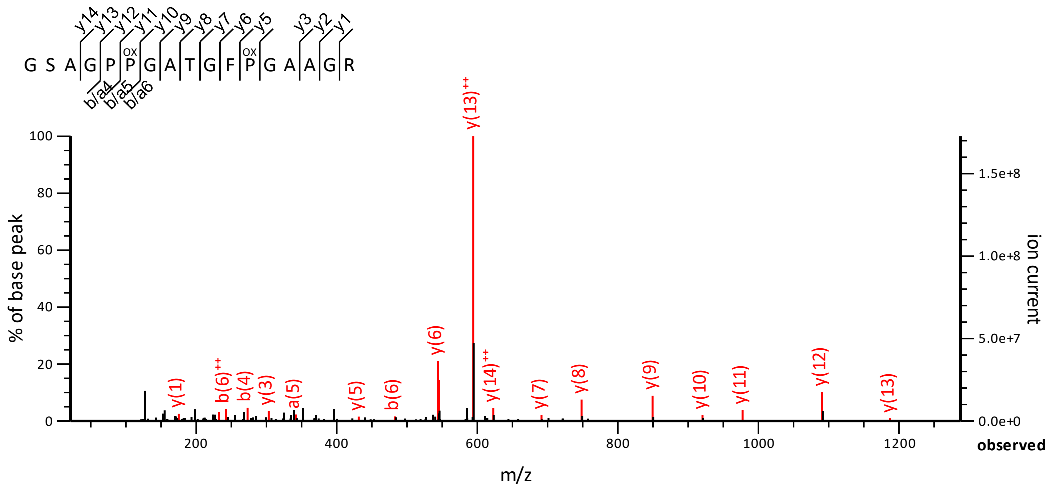

COL1A1 220 - 237

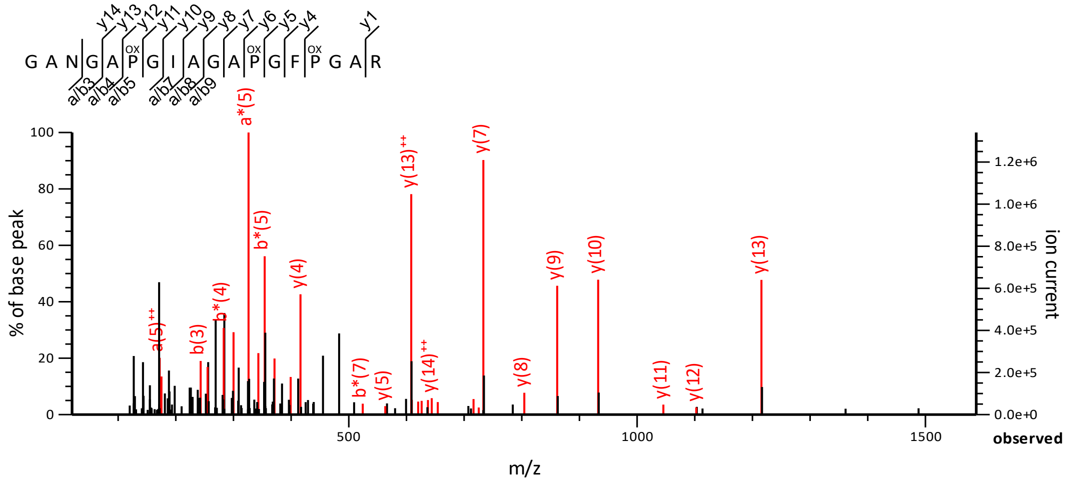

COL1A1 934 - 963

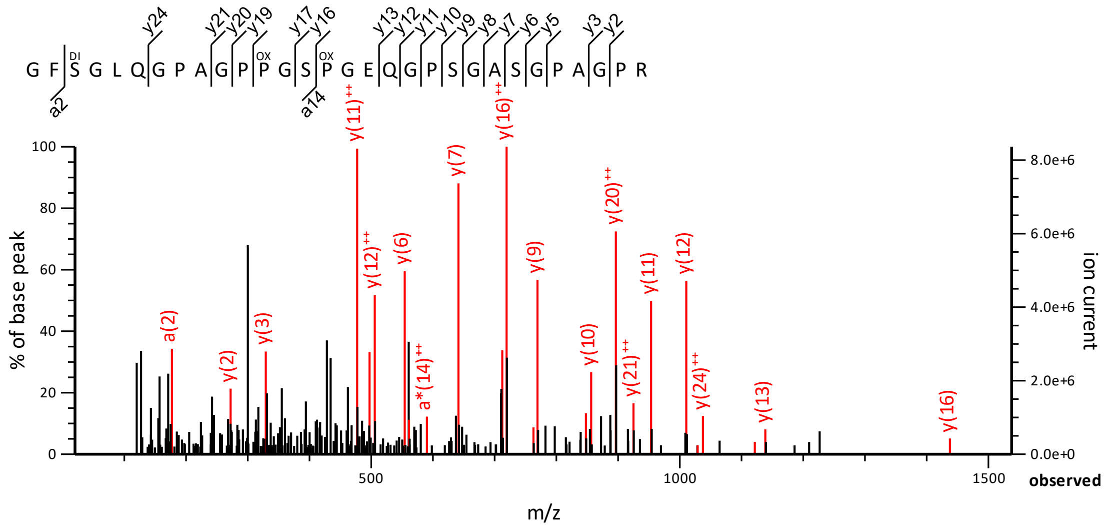

COL1A1 586 - 618

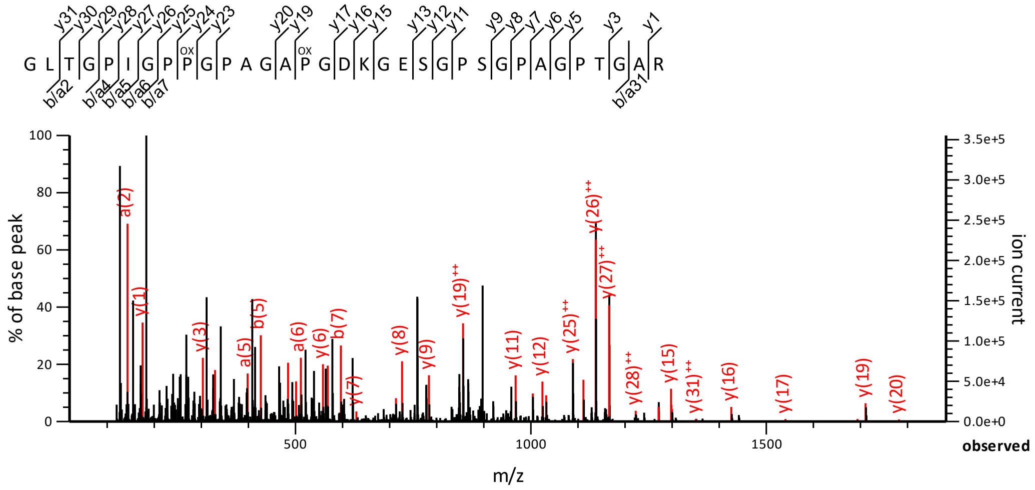

COL1A2 978 - 990

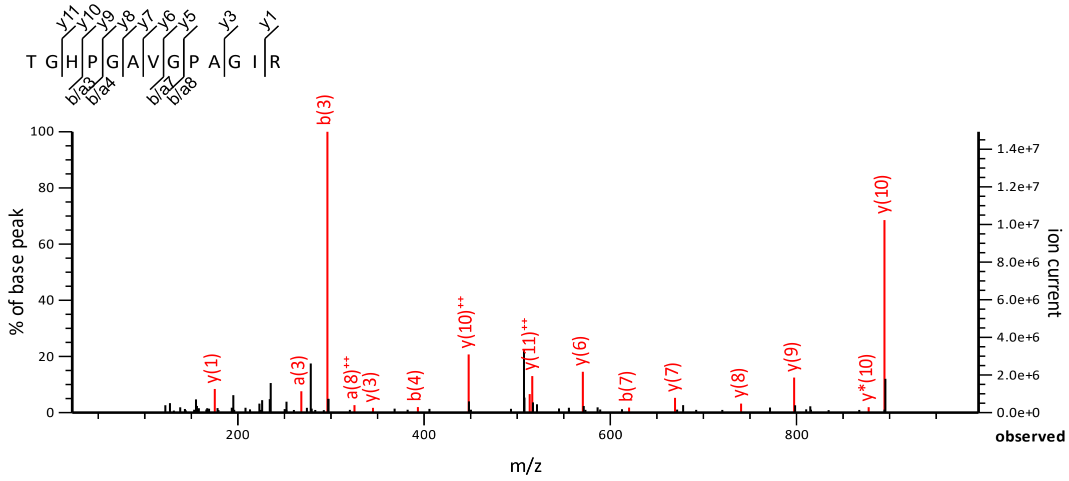

COL1A2 484 - 498

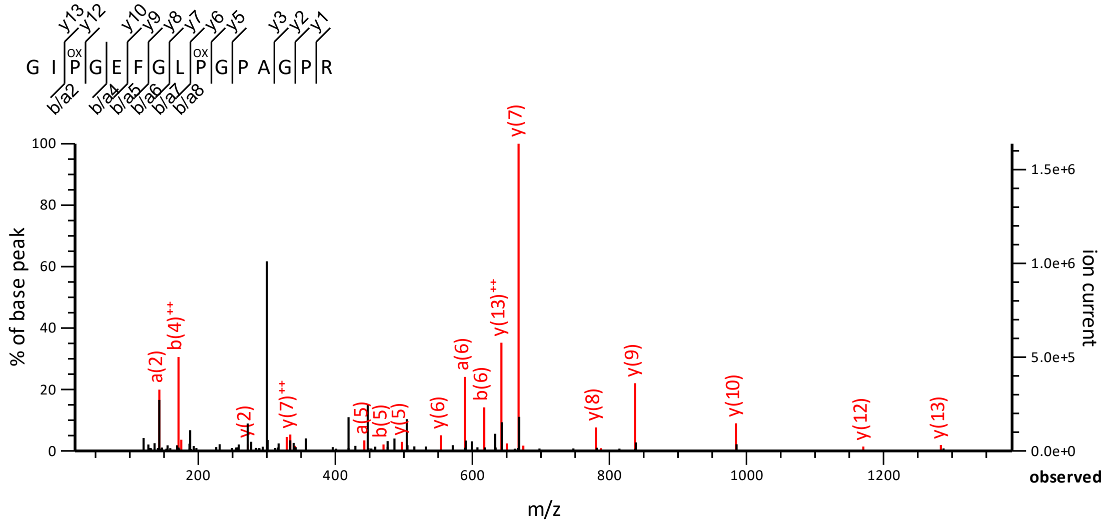

COL1A2 889 - 906

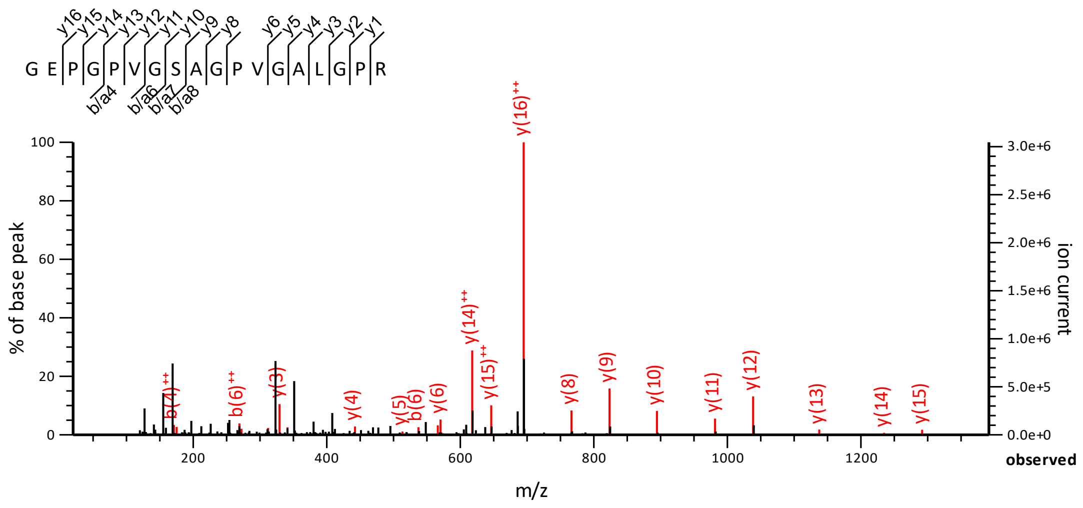

COL1A2 793 - 816

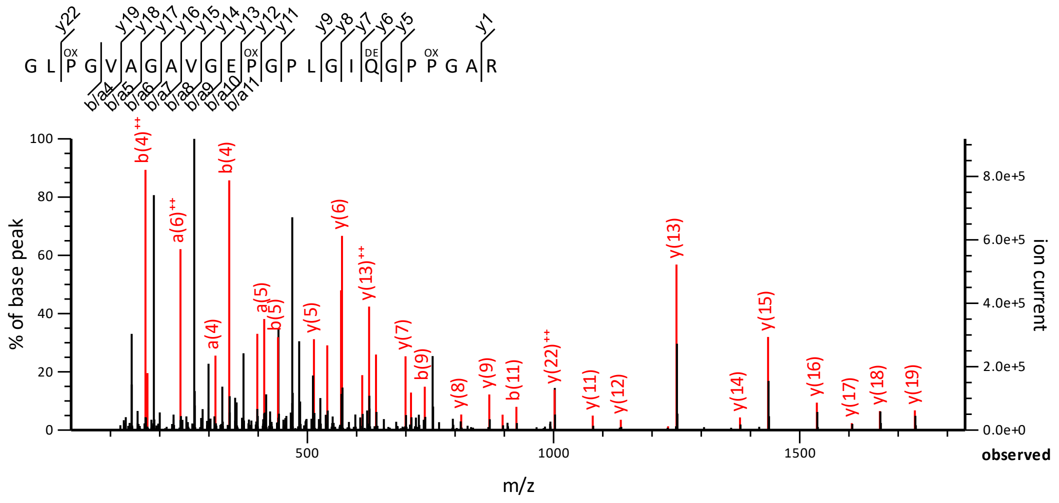

COL1A2 658 - 687

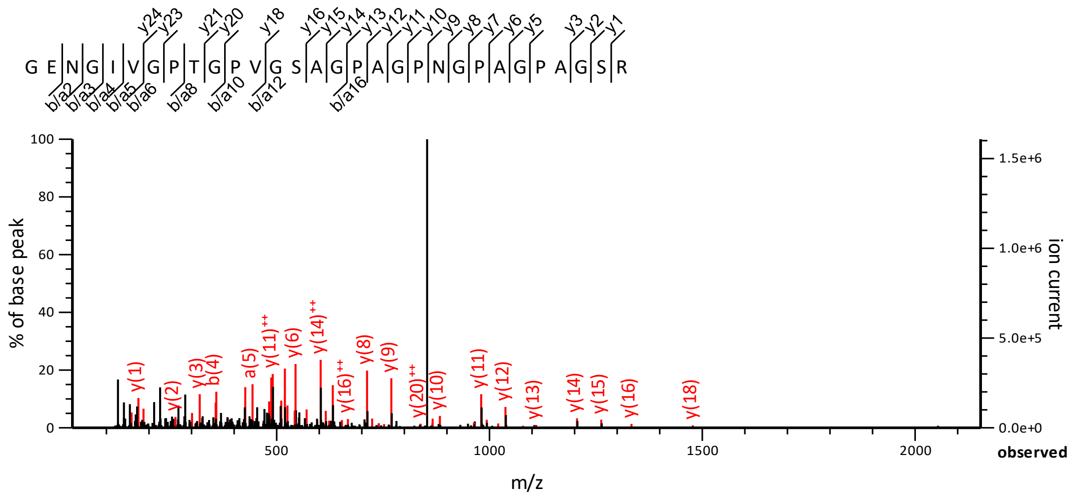

COL1A2 757 - 789

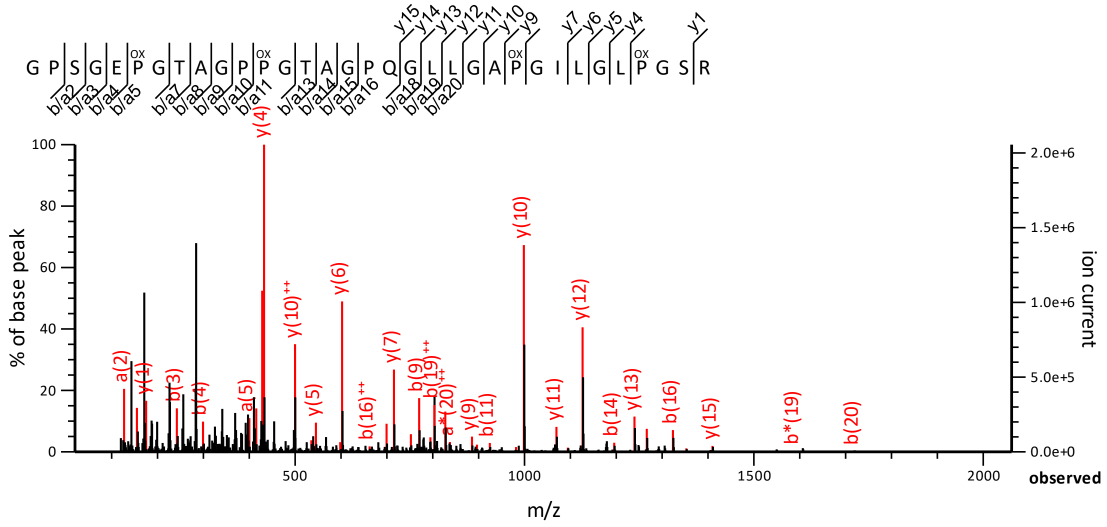

COL1A2 10-42

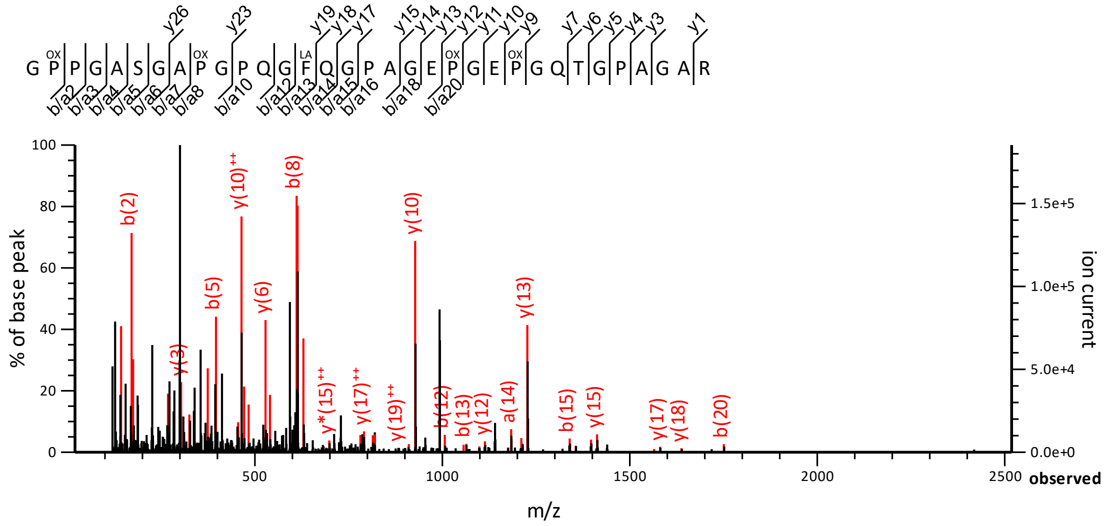

Dasypus novemcinctus

COL1A1 508 - 519

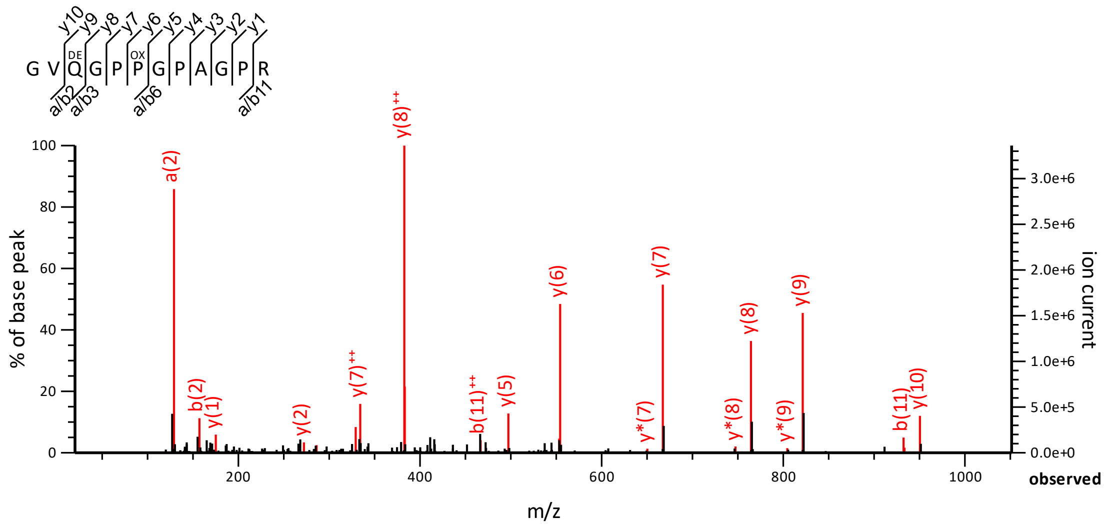

COL1A1 688 - 704

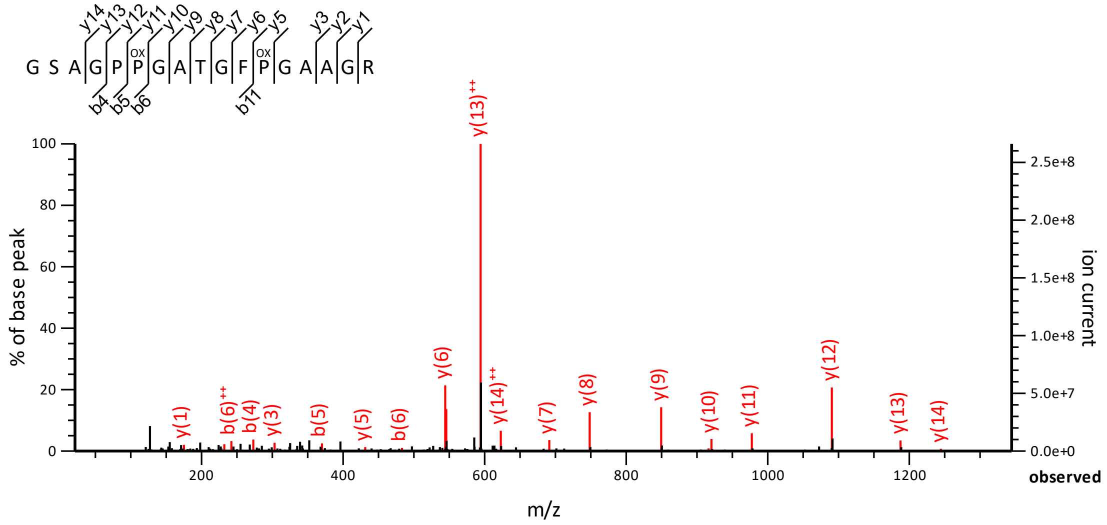

COL1A1 220 - 237

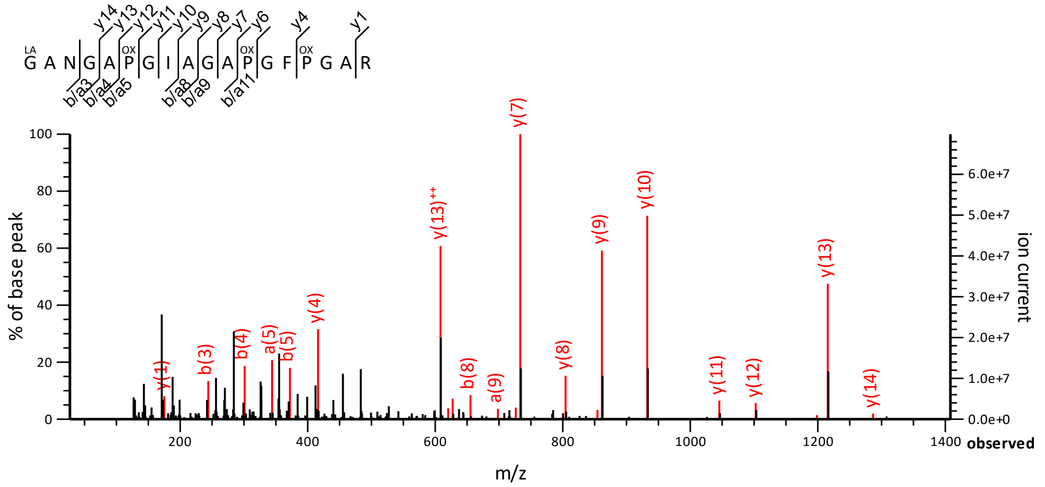

COL1A1 934 - 963

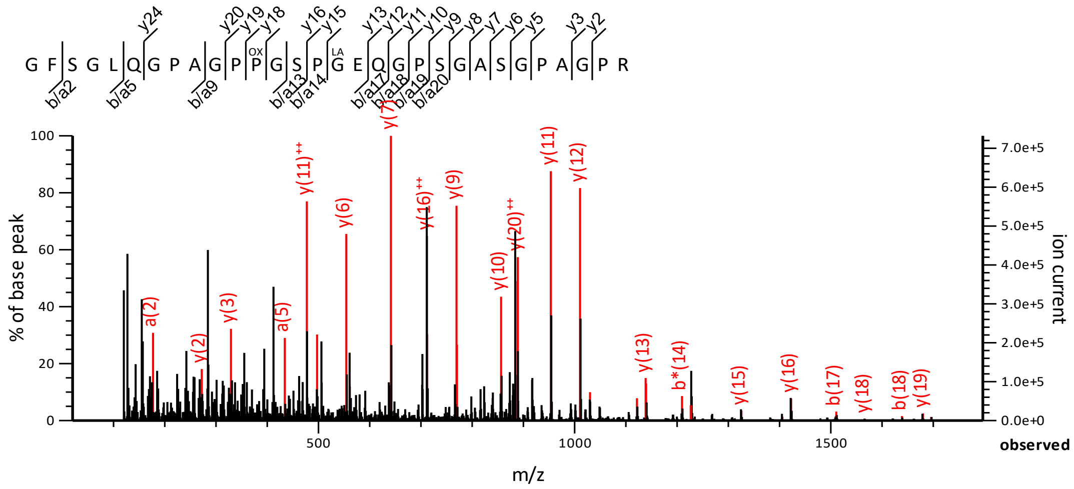

COL1A1 586 - 618

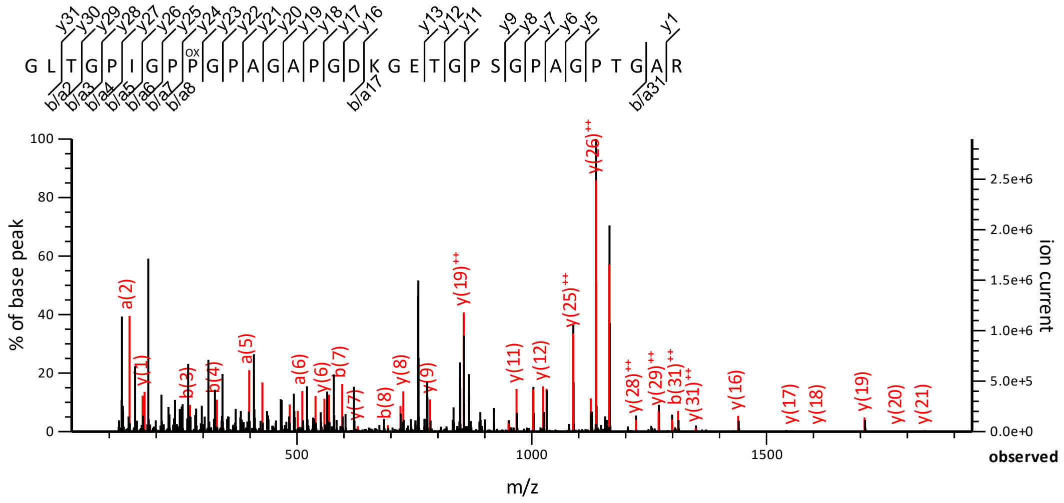

COL1A2 978 - 990

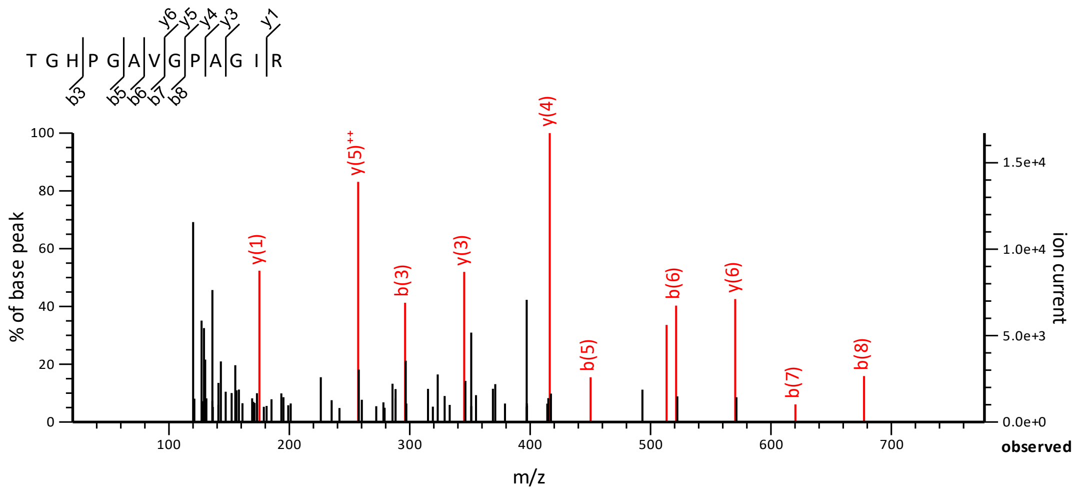

COL1A2 484 - 498

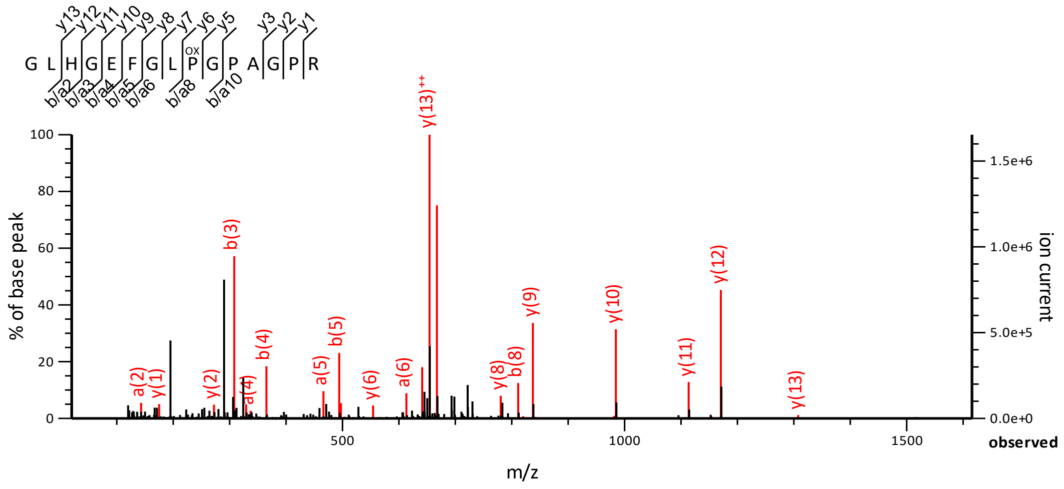

COL1A2 889 - 906

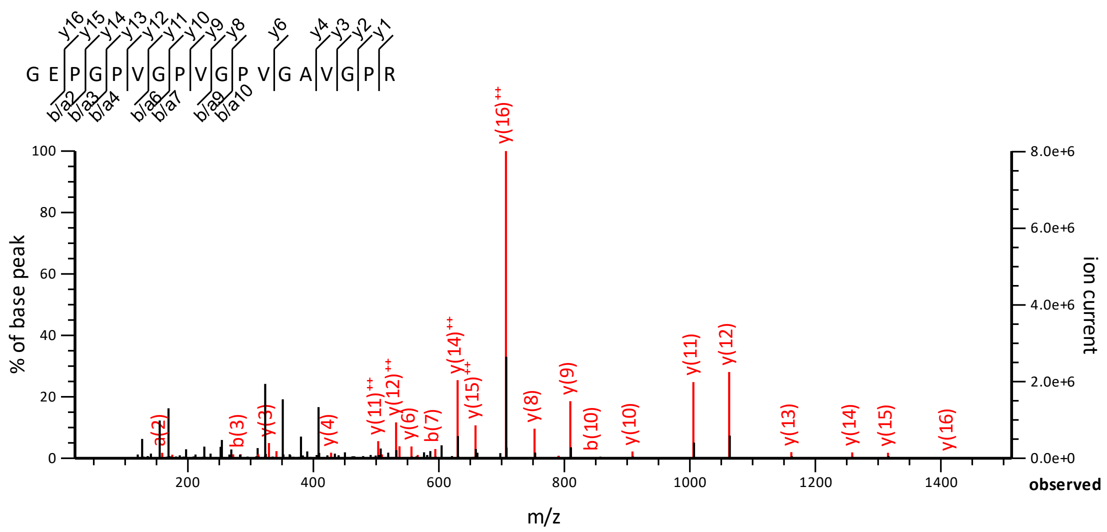

COL1A2 793 - 816

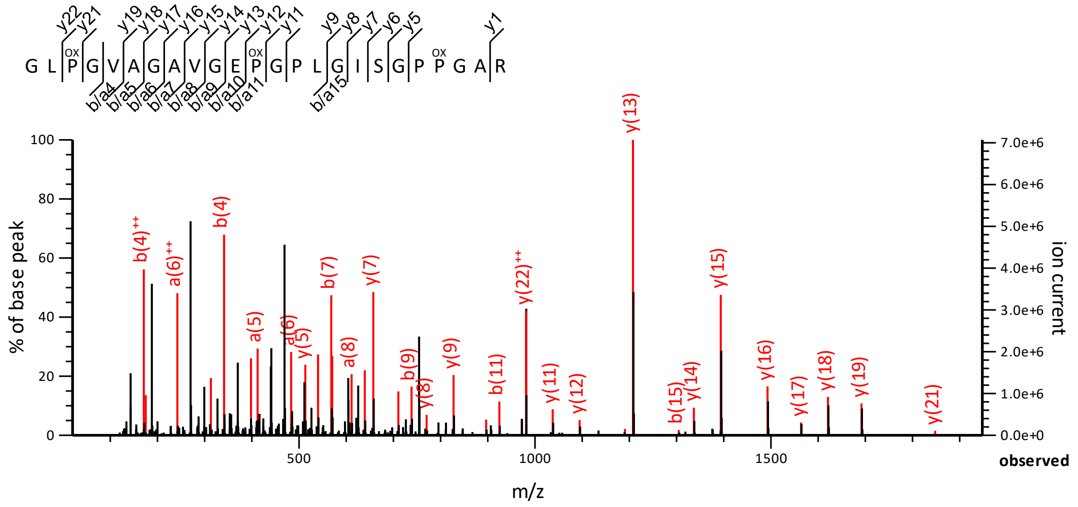

COL1A2 658 - 687

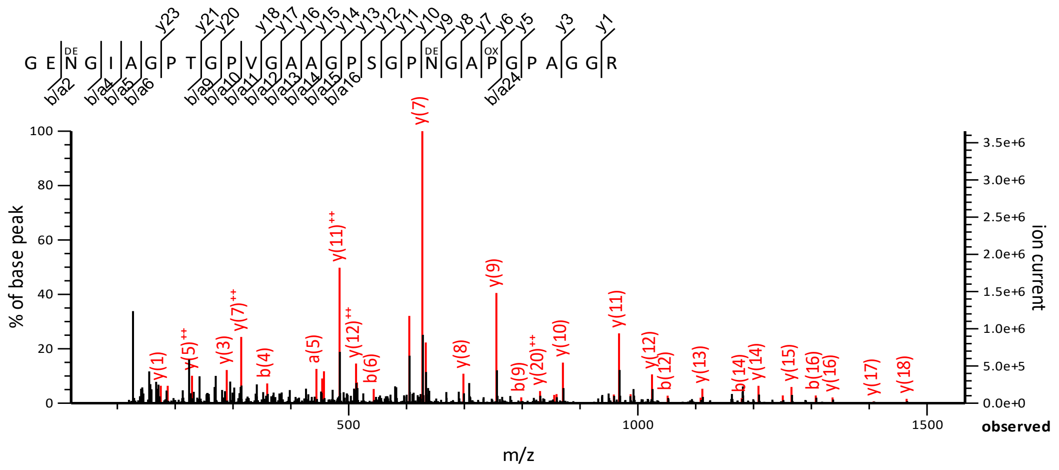

COL1A2 757 - 789

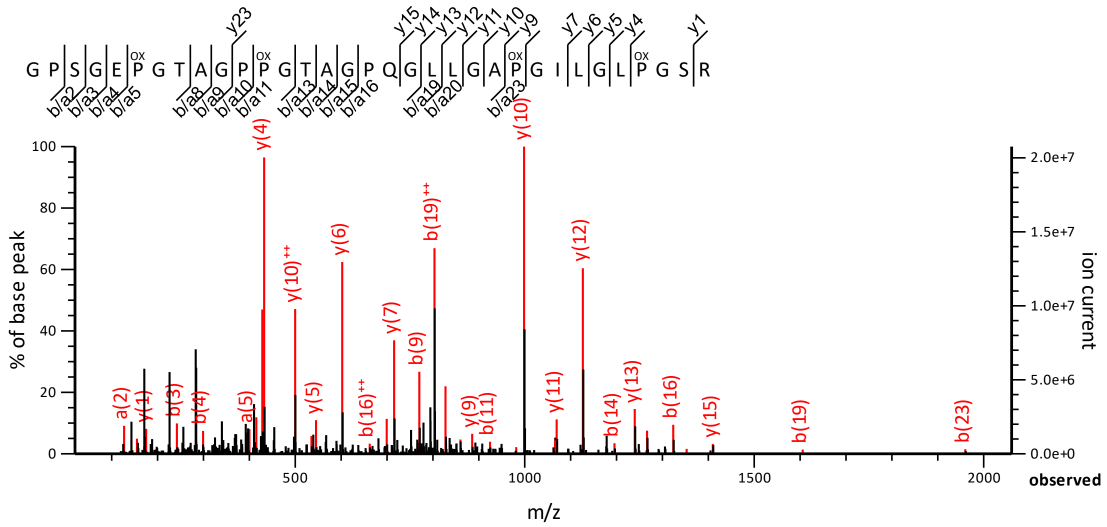

COL1A2 10-42

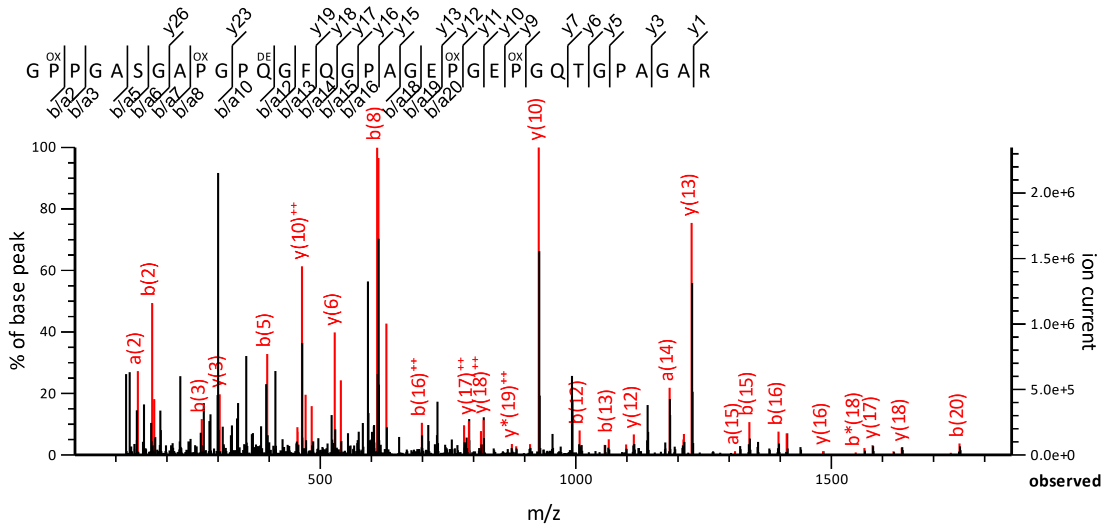

Glyptodon sp.

COL1A1 508 - 519

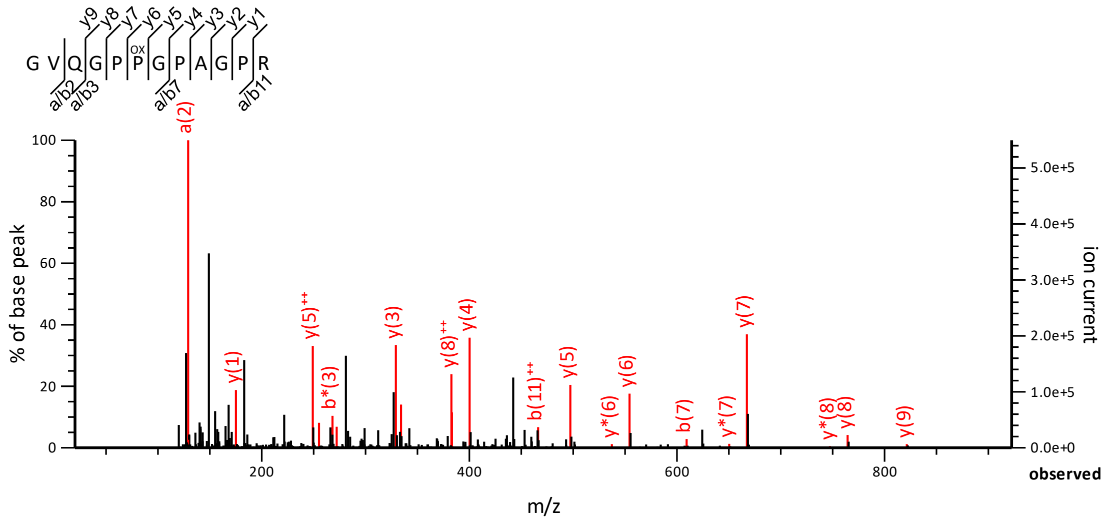

COL1A1 688 - 704

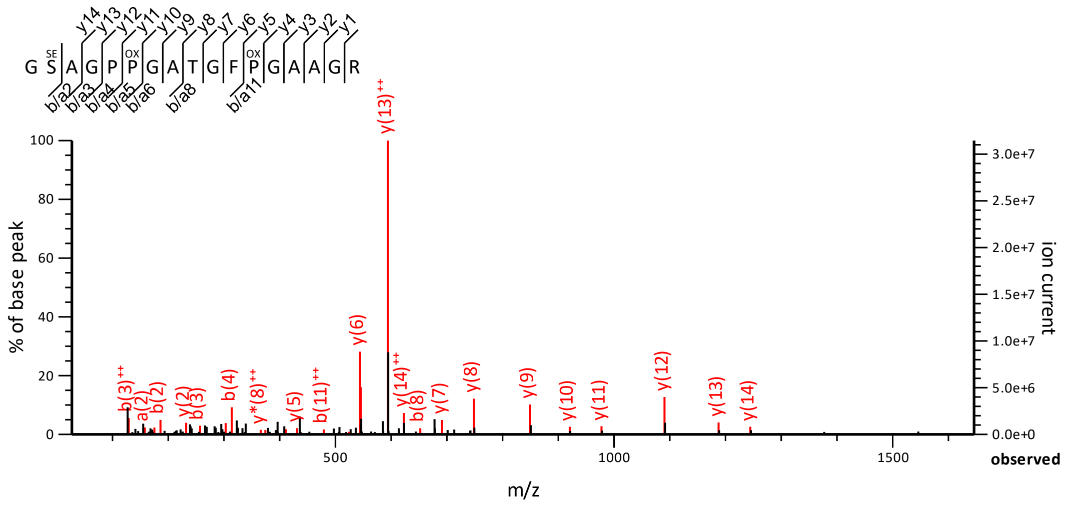

COL1A1 220 - 237

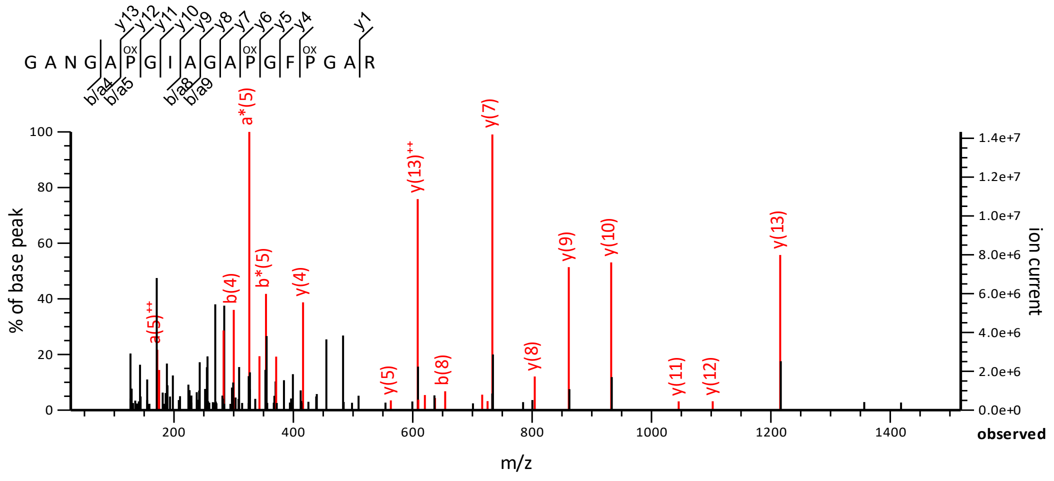

COL1A1 934 - 963

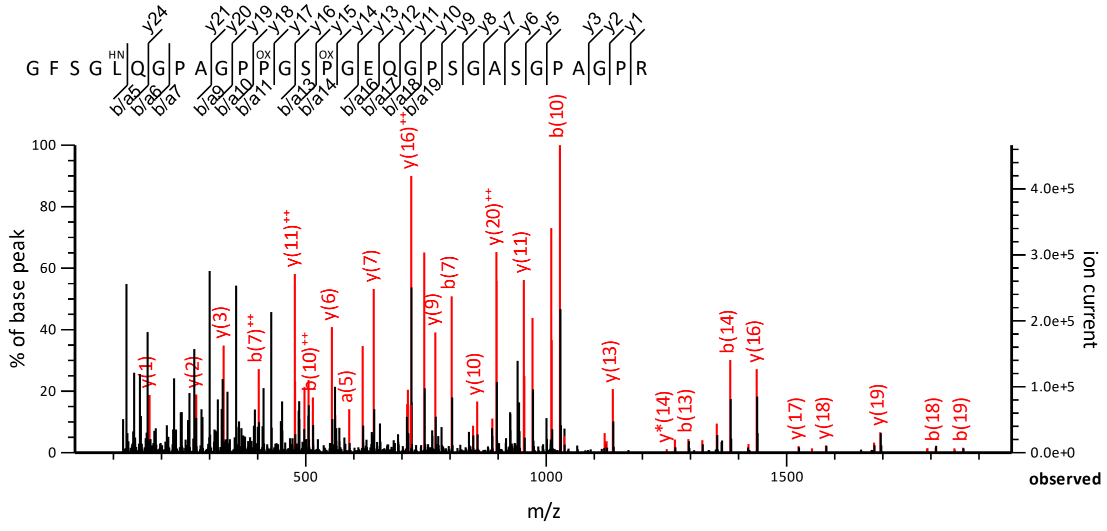

COL1A1 586 - 618

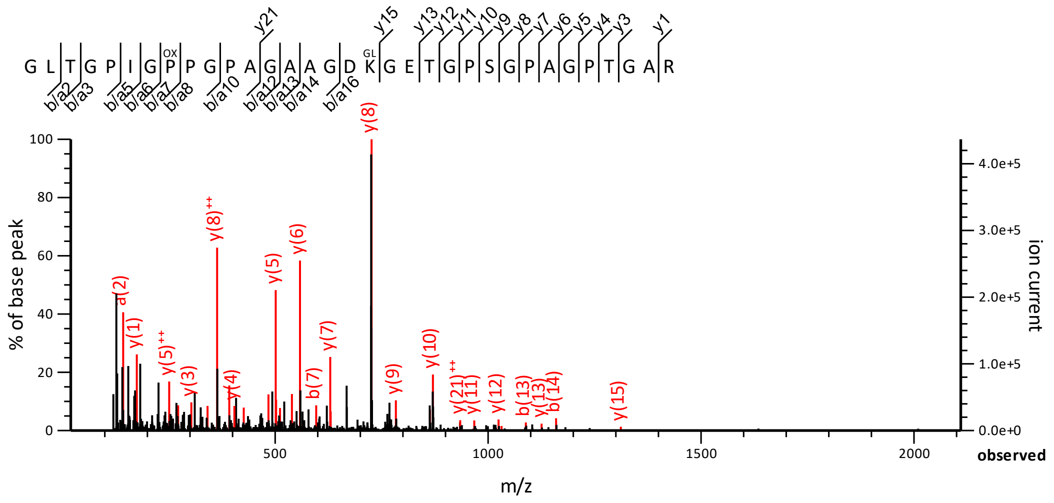

COL1A2 978 - 990

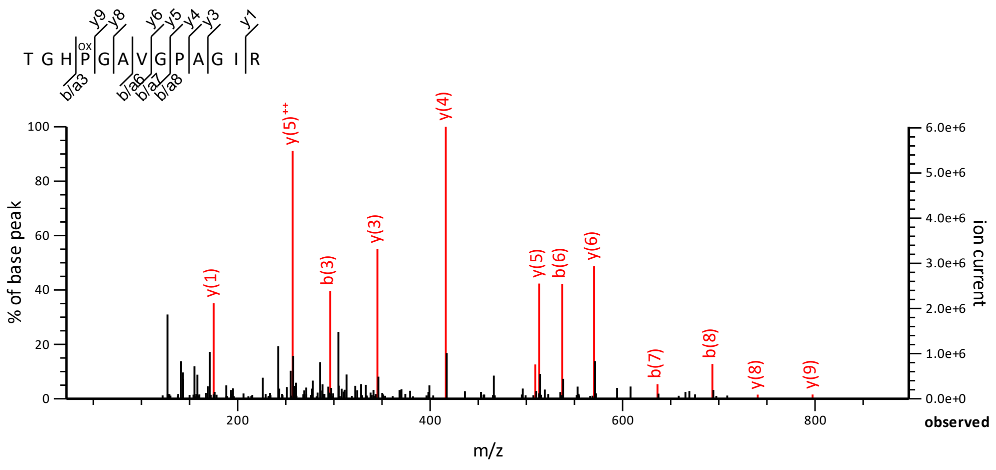

COL1A2 484 - 498

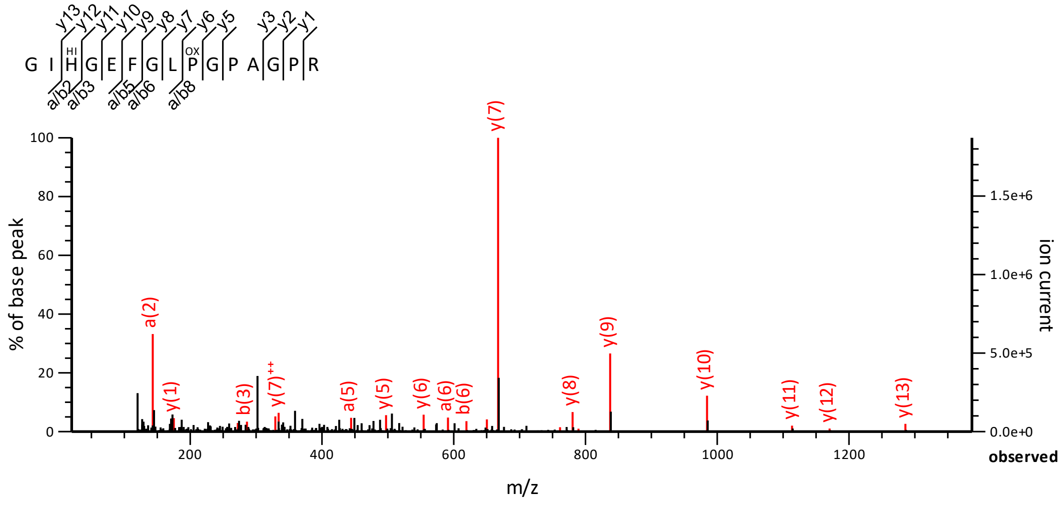

COL1A2 889 - 906

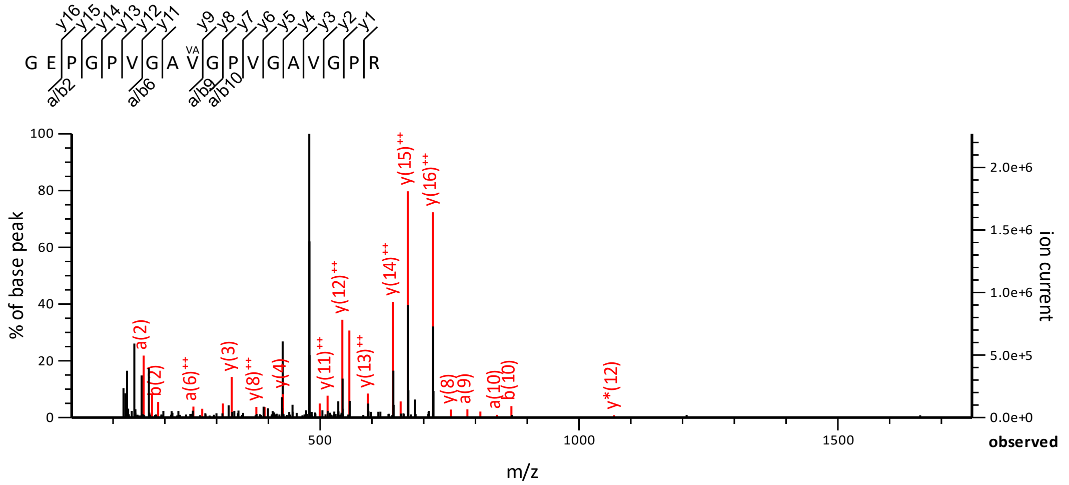

COL1A2 793 - 816

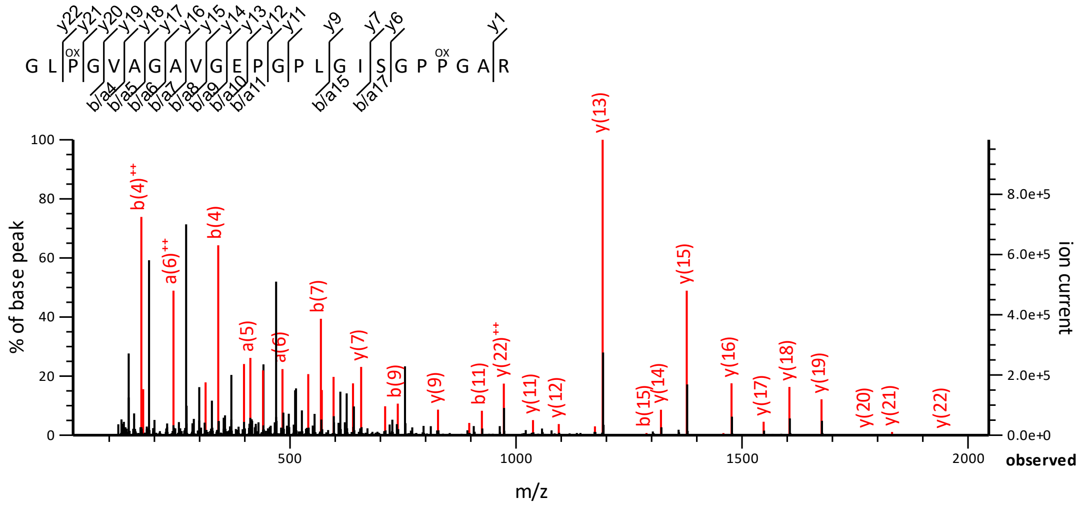

COL1A2 658 - 687

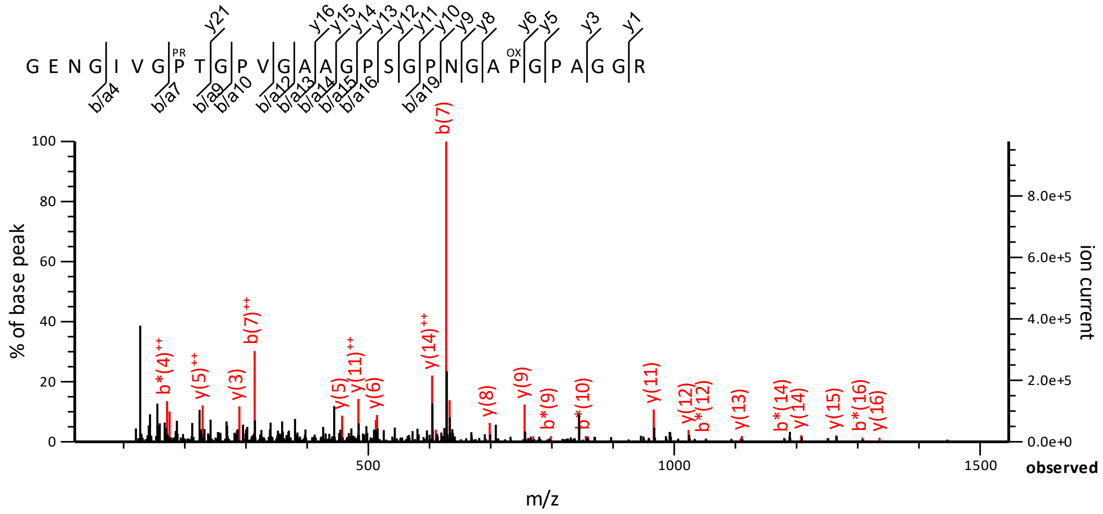

COL1A2 757 - 789

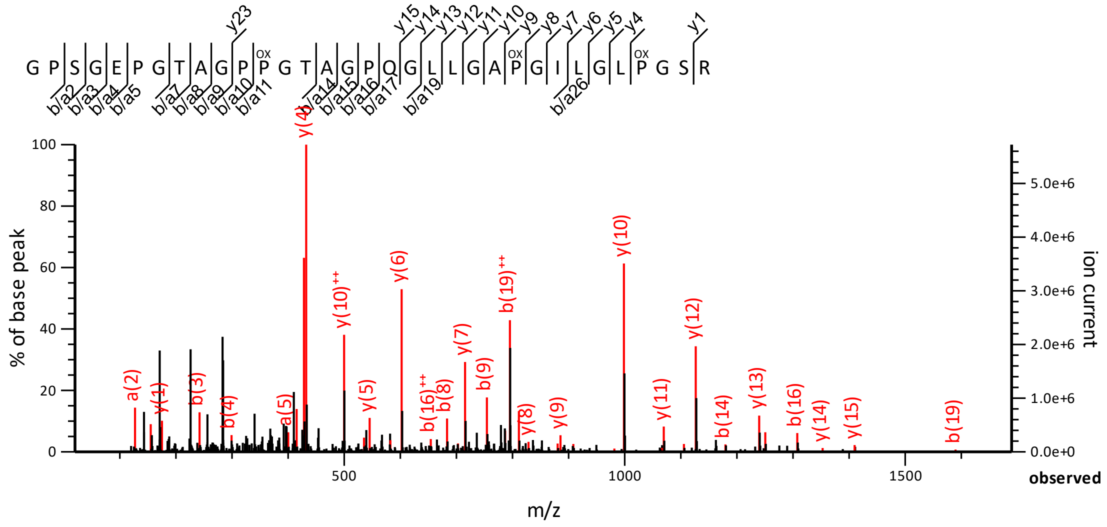

COL1A2 10-42

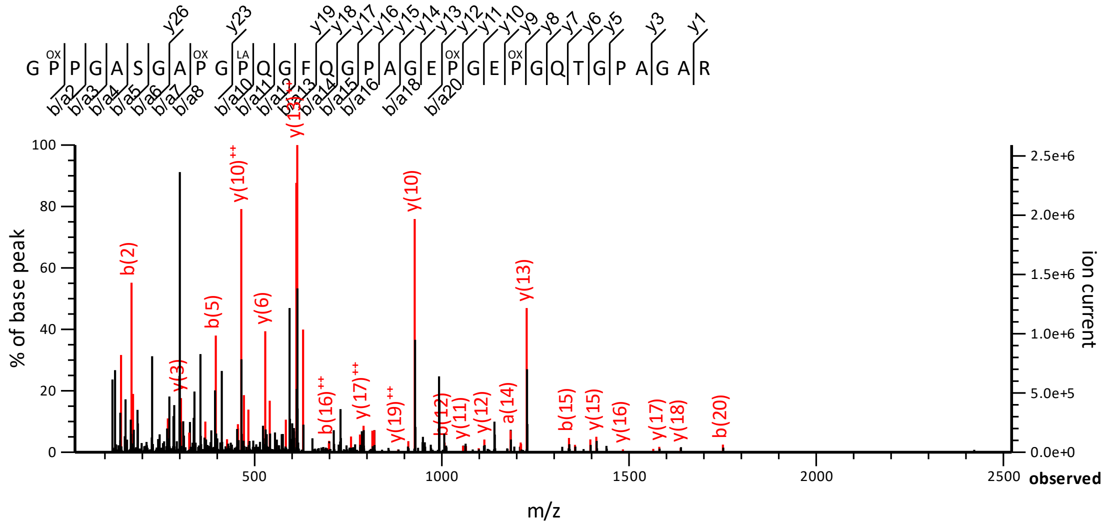

Mylodon sp.

COL1A1 508 - 519

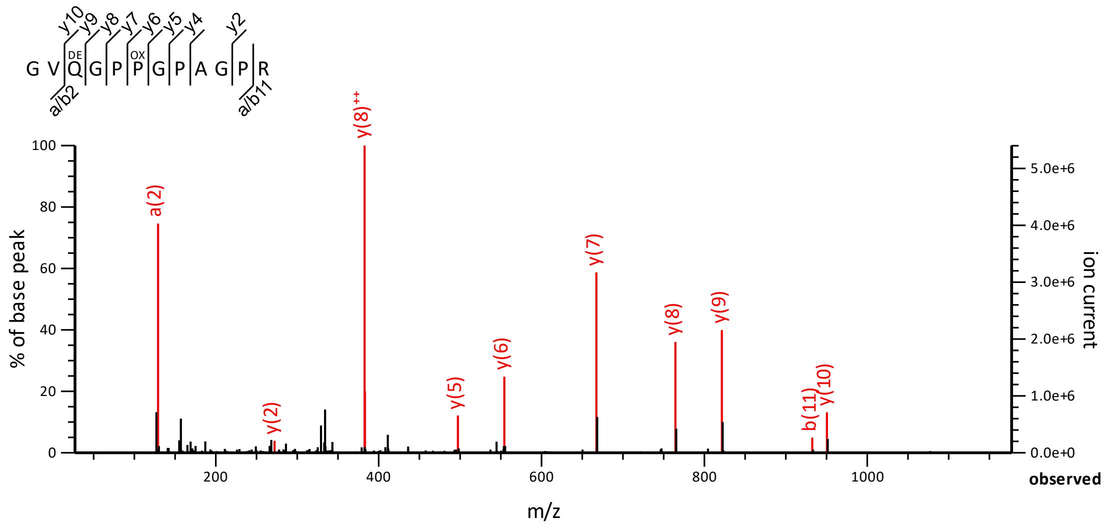

COL1A1 688 - 704

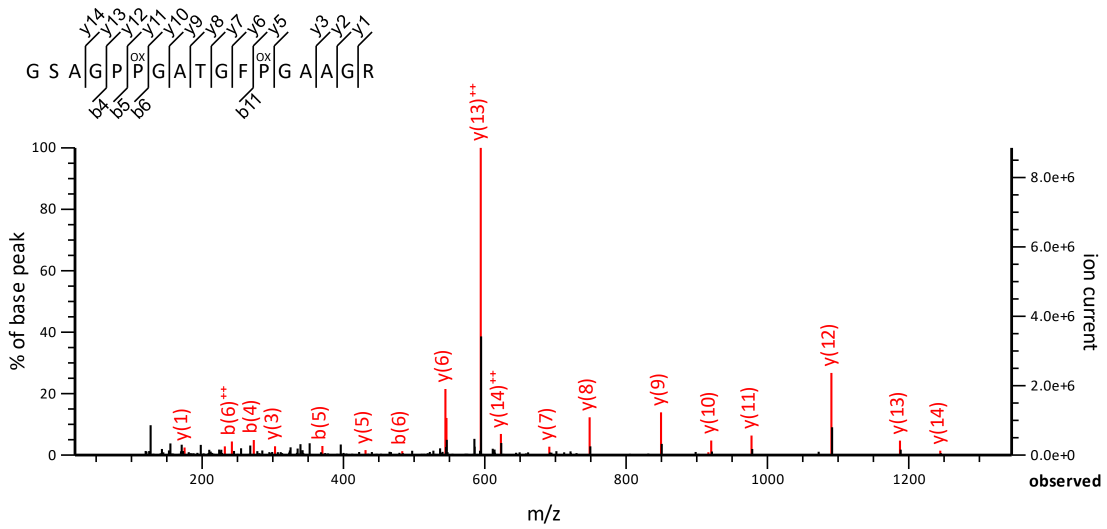

COL1A1 220 - 237

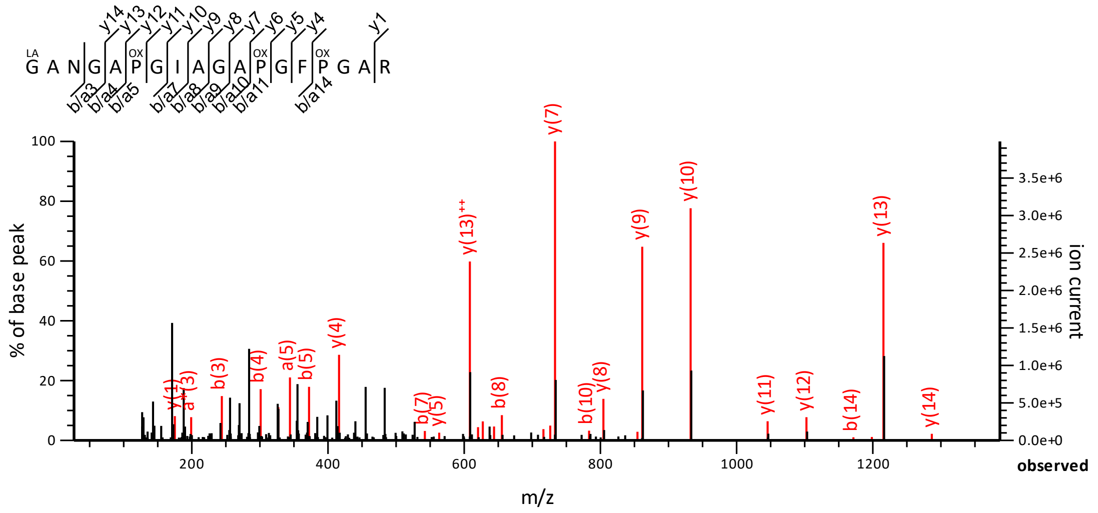

COL1A1 934 - 963

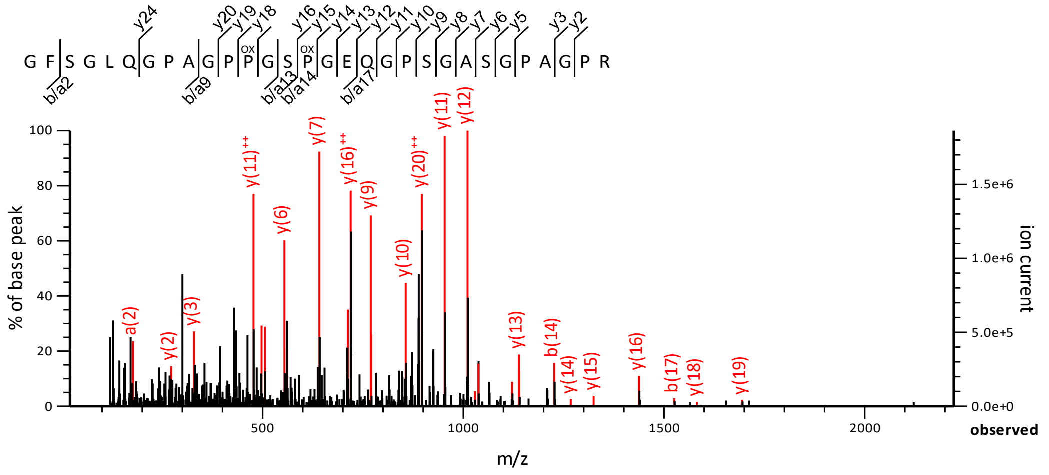

COL1A1 586 - 618

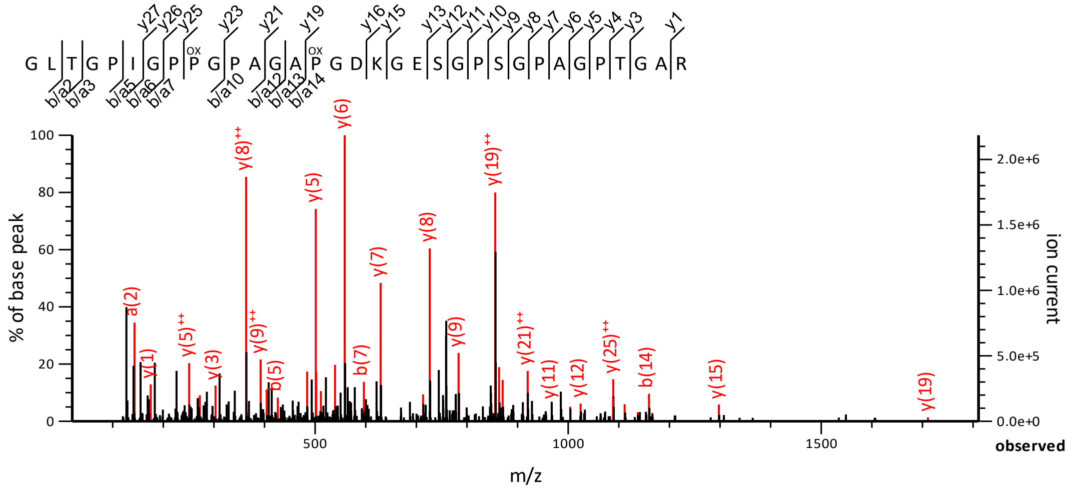

COL1A2 978 - 990

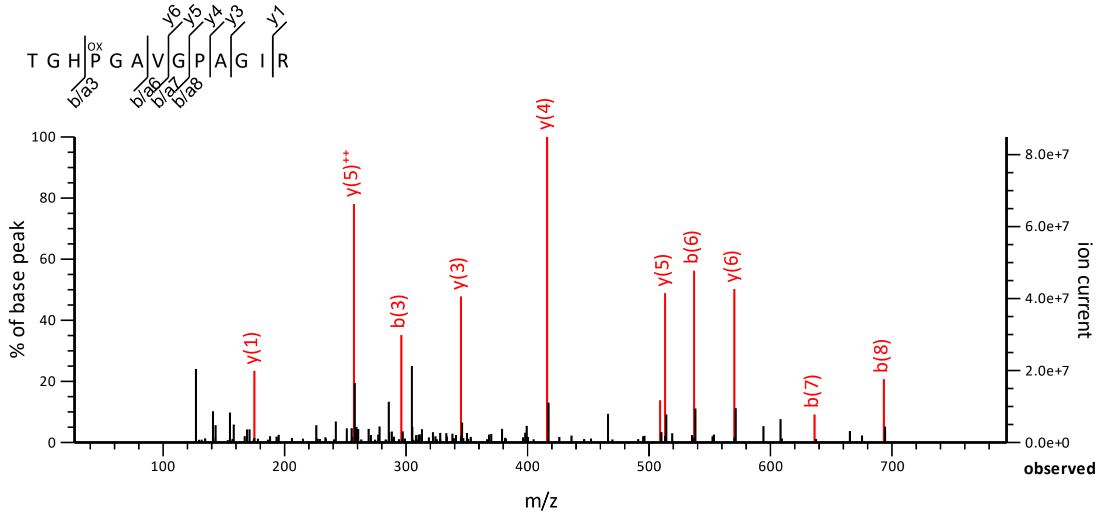

COL1A2 484 - 498

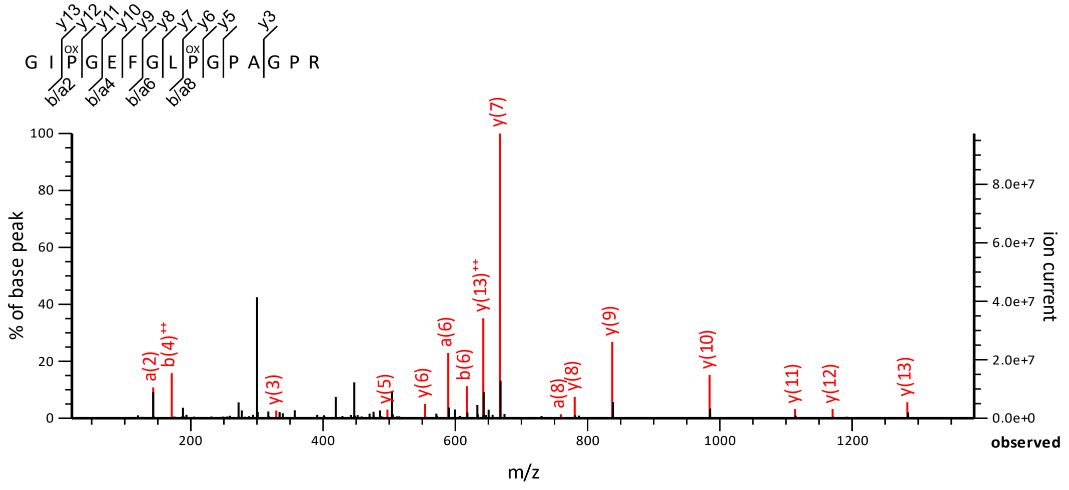

COL1A2 889 - 906

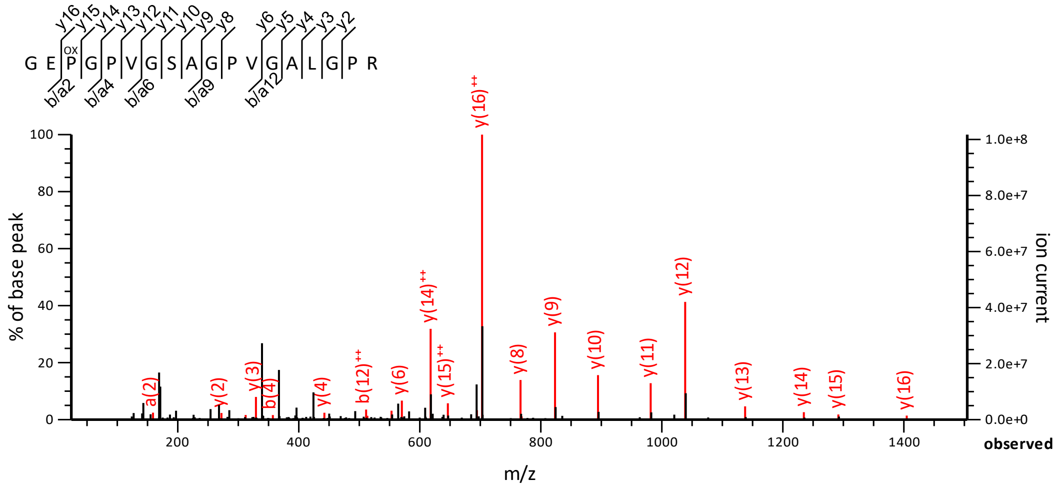

COL1A2 793 - 816

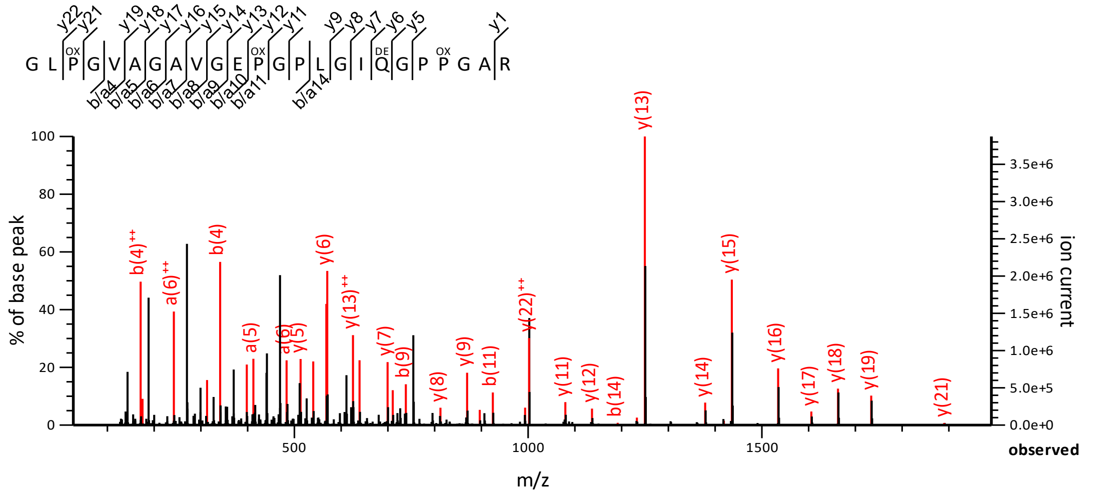

COL1A2 658 - 687

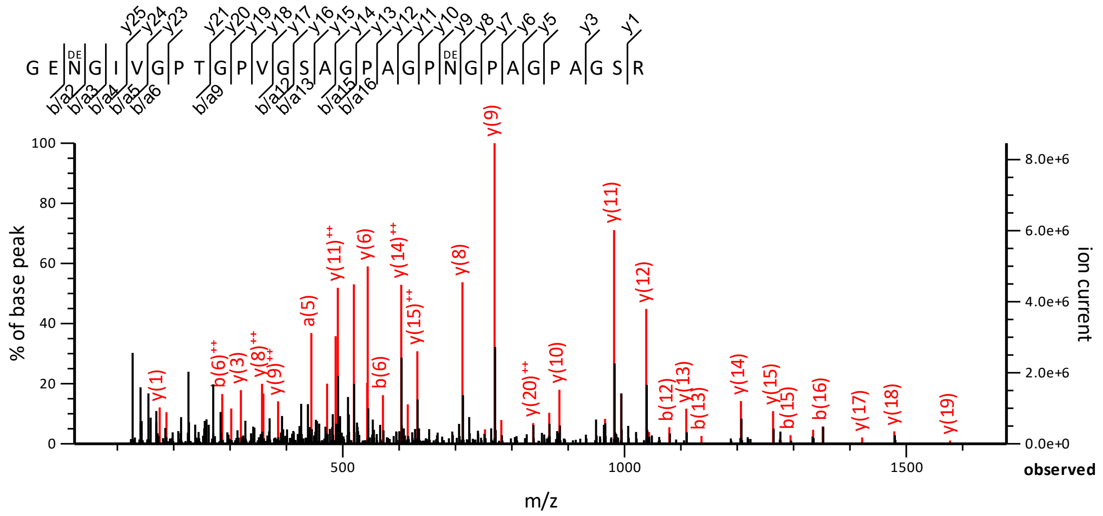

COL1A2 757 - 789

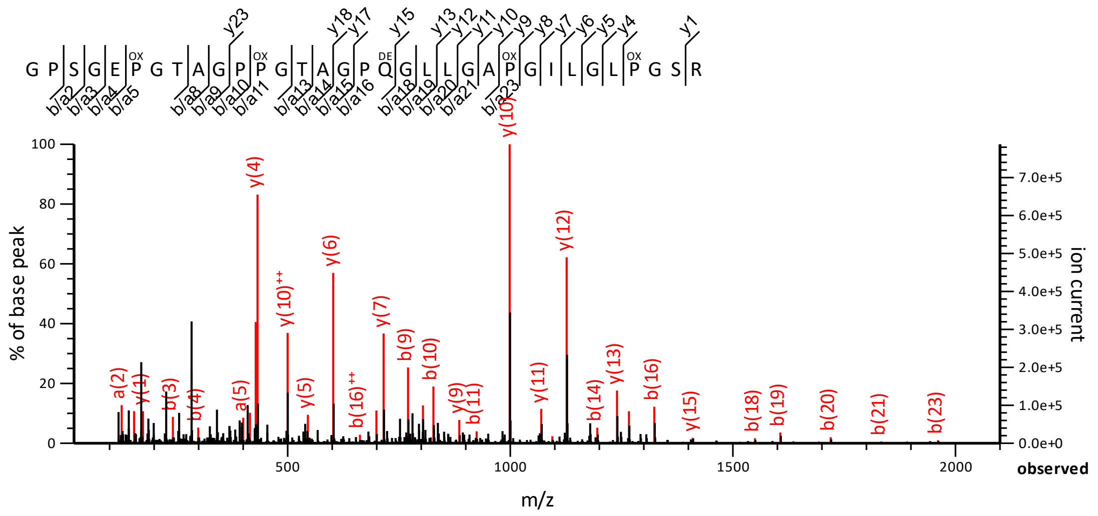

COL1A2 10-42

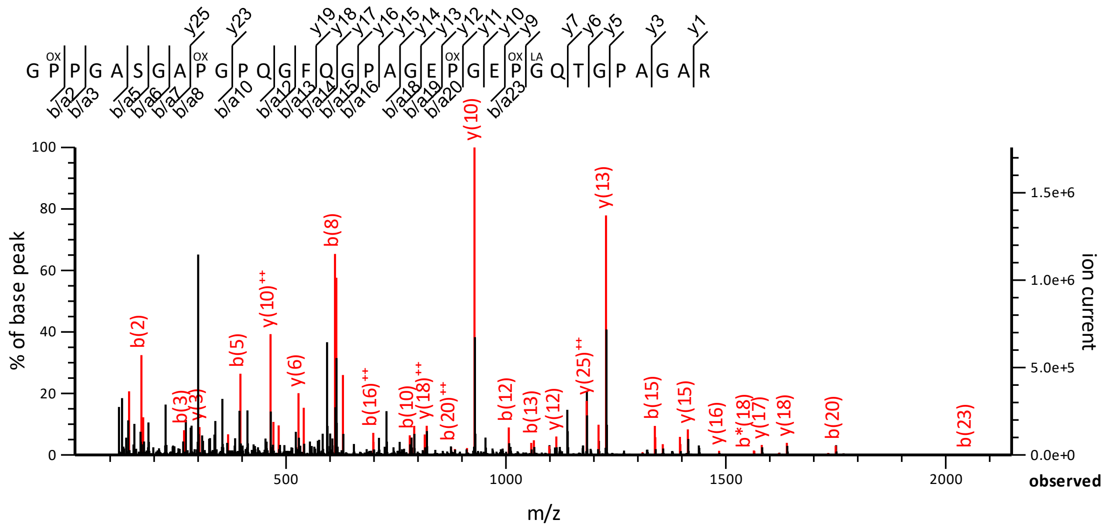

Myrmecophaga tridactyla

COL1A1 508 – 519

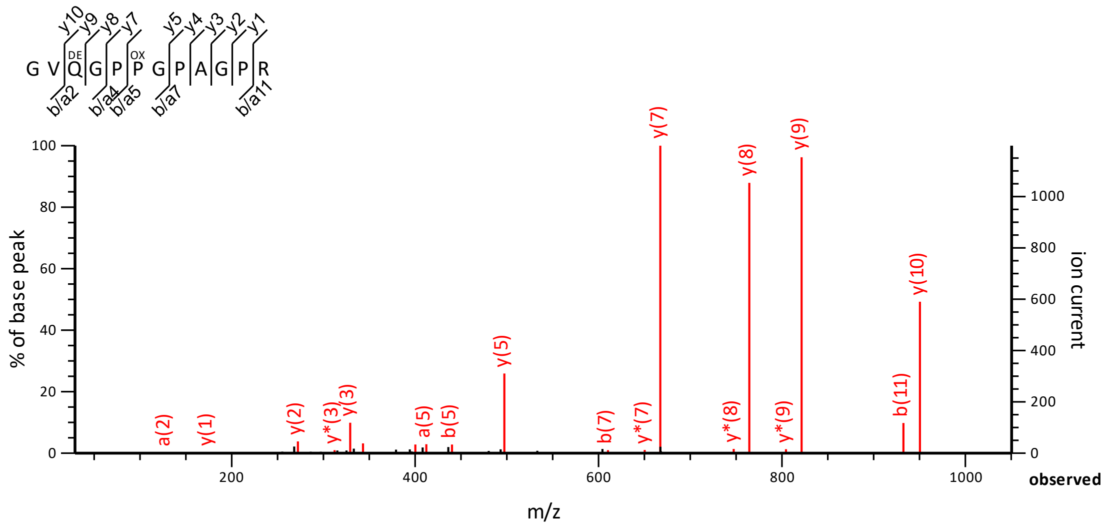

COL1A1 688 - 704

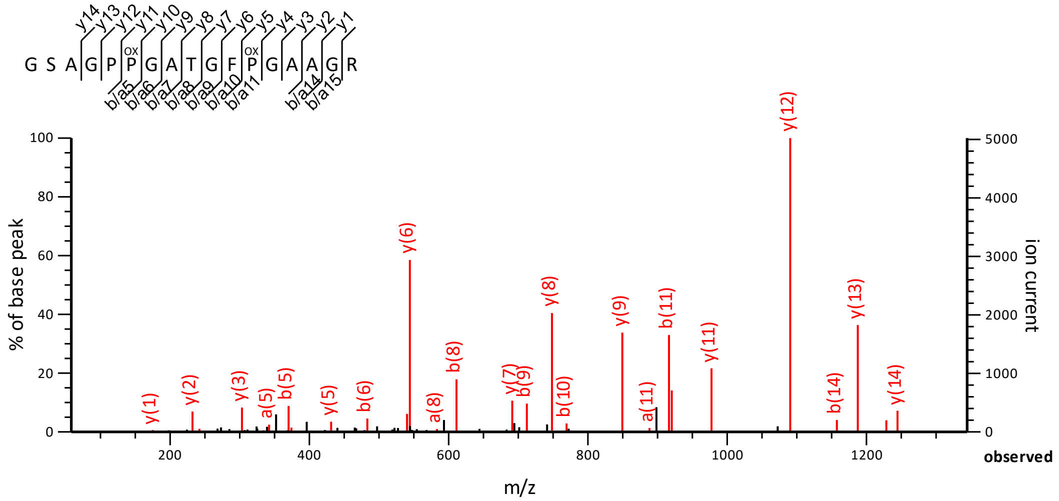

COL1A1 220 - 237

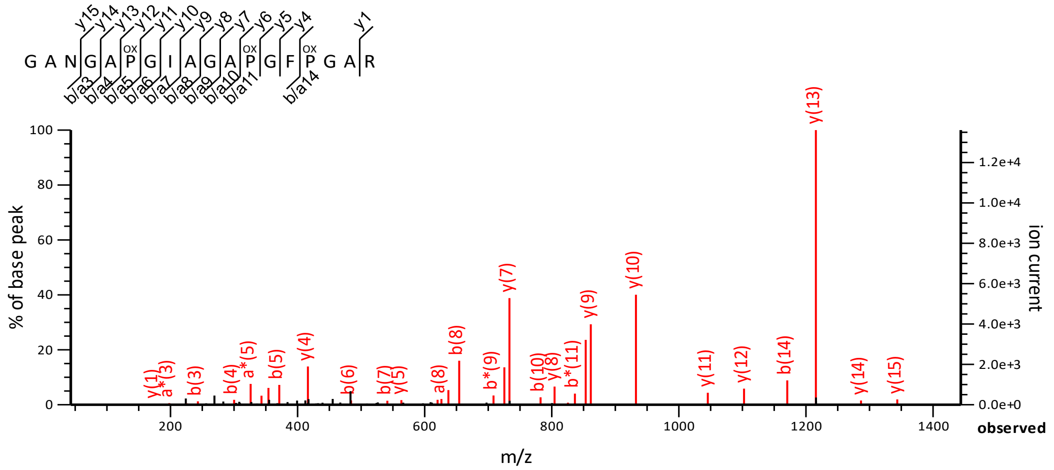

COL1A1 934 - 963

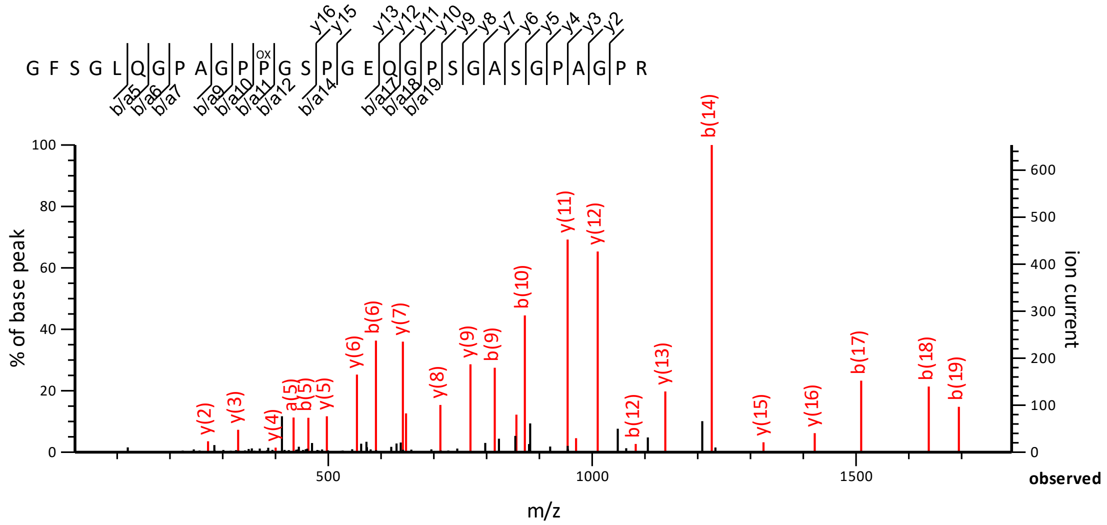

COL1A1 586 - 618

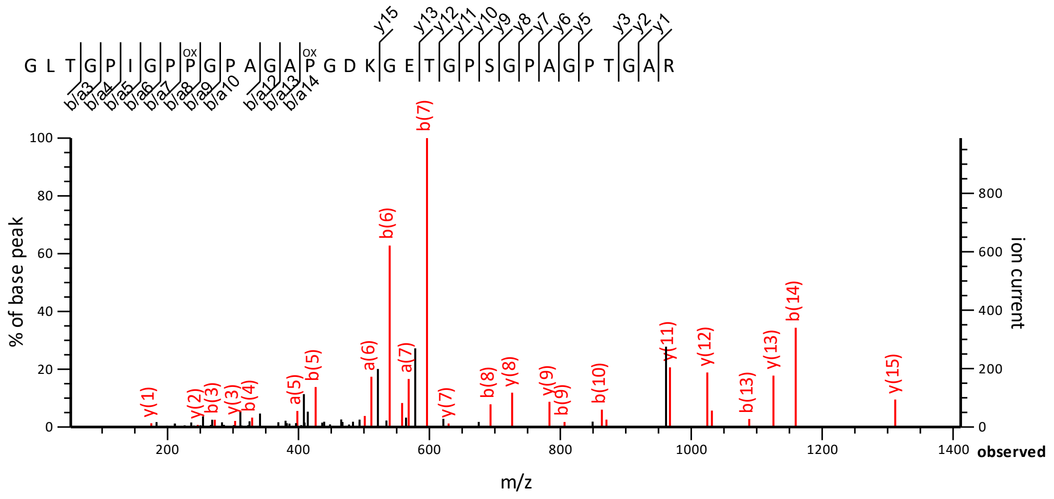

COL1A2 978 - 990

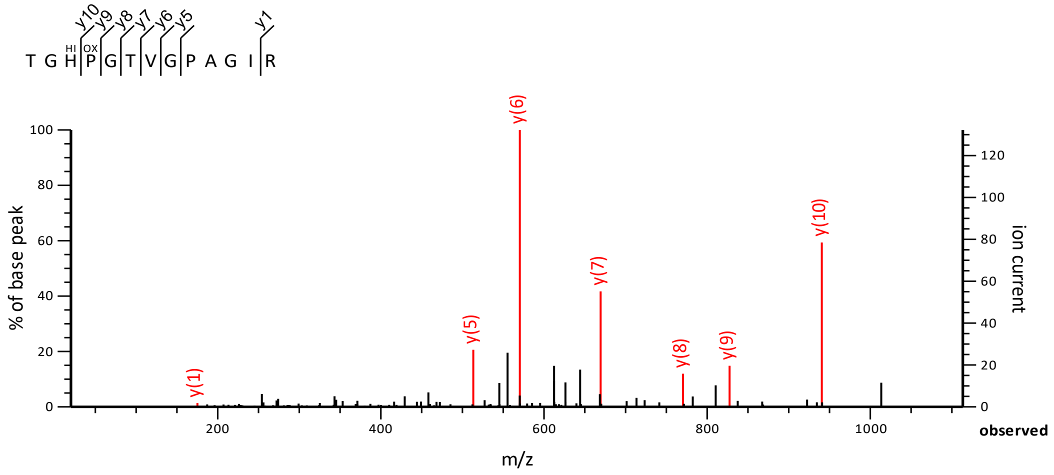

COL1A2 484 - 498

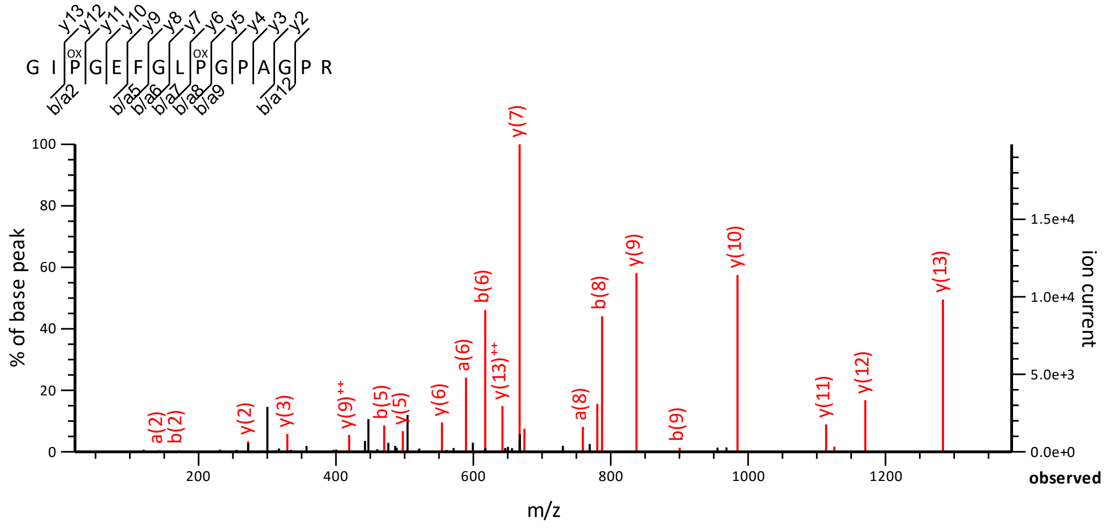

COL1A2 889 - 906

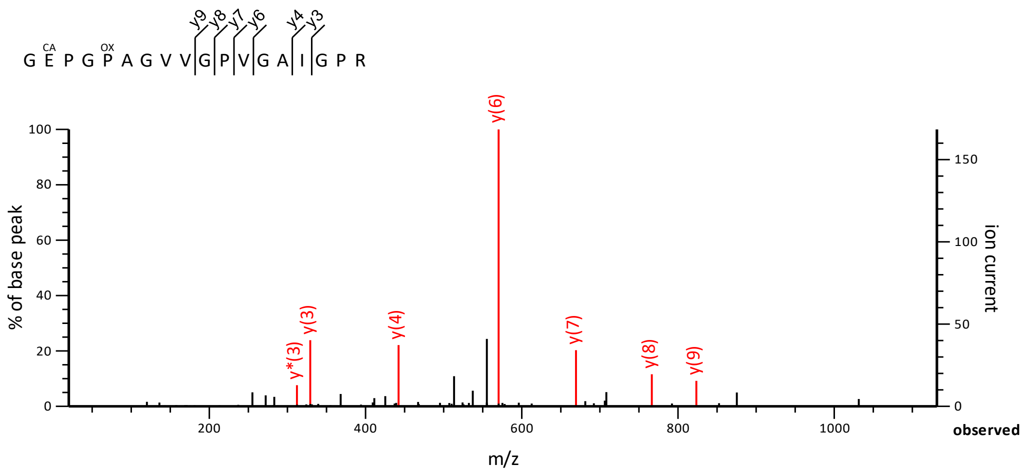

COL1A2 793 - 816

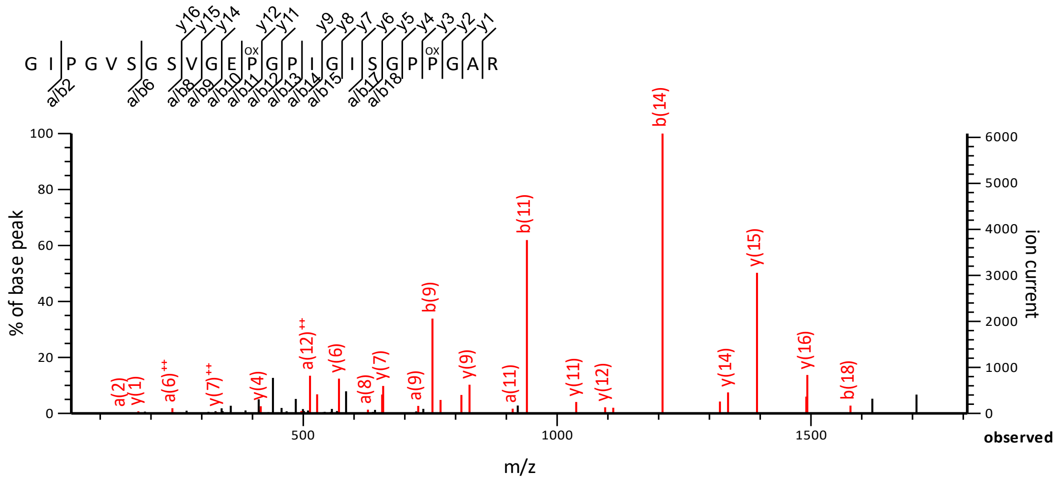

COL1A2 757 - 789

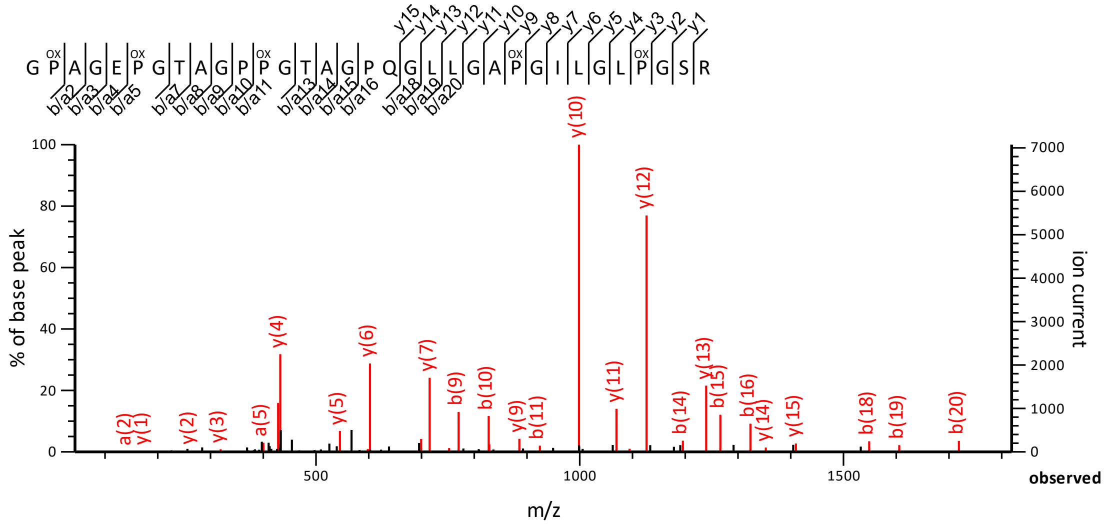

COL1A2 10-42

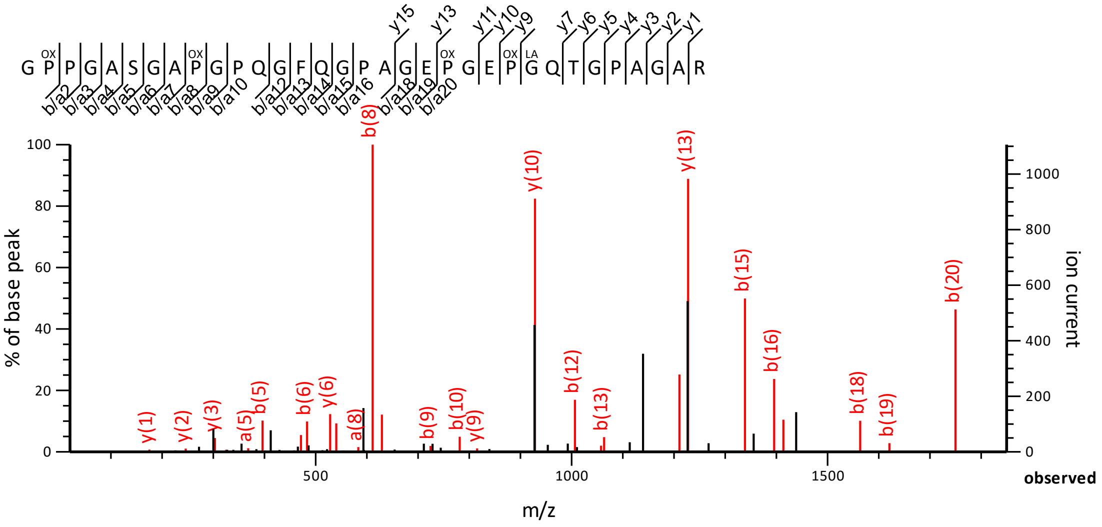

Priodontes maximus

COL1A1 508 - 519

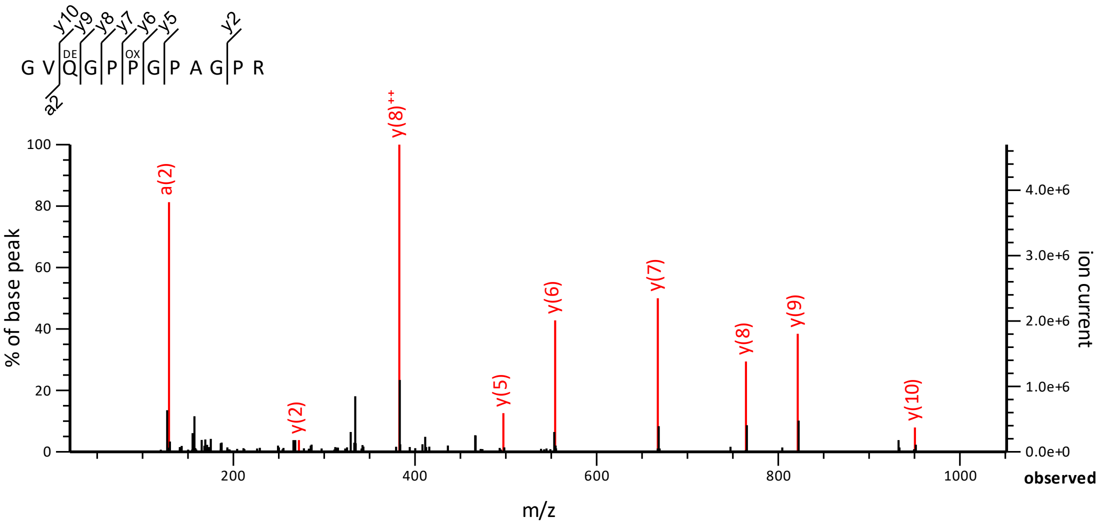

COL1A1 688 - 704

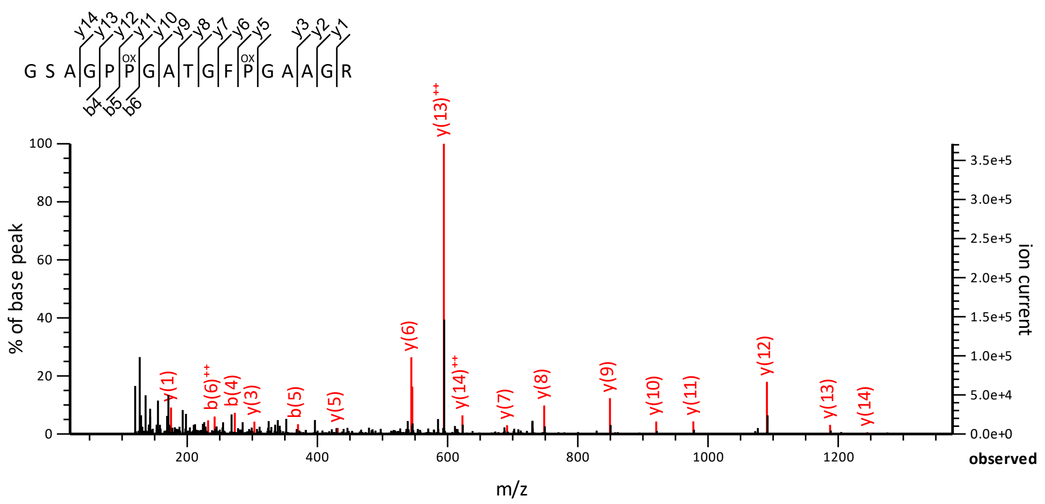

COL1A1 220 - 237

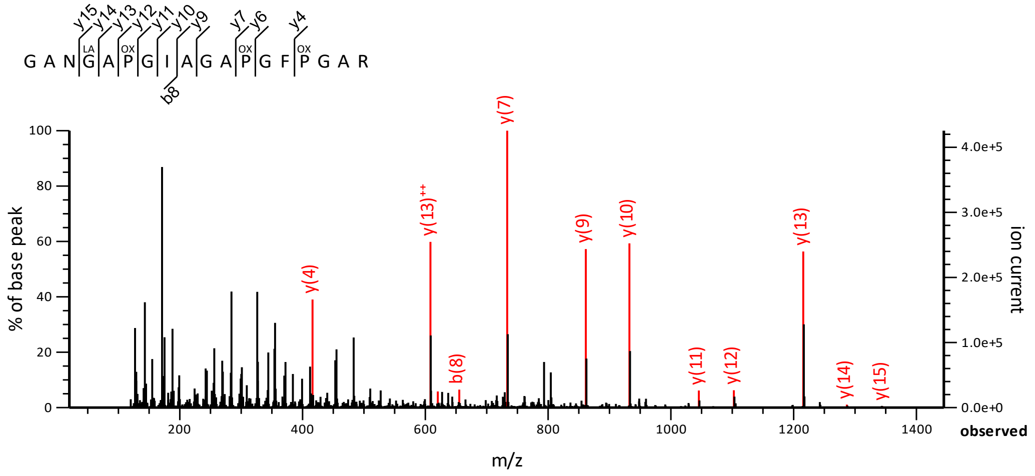

COL1A1 934 - 963

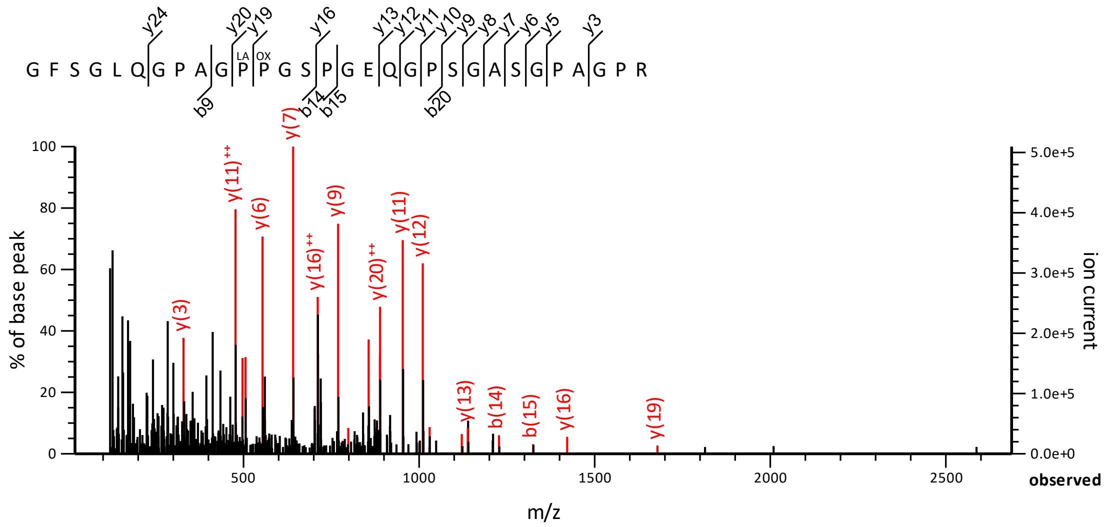

COL1A1 586 - 618

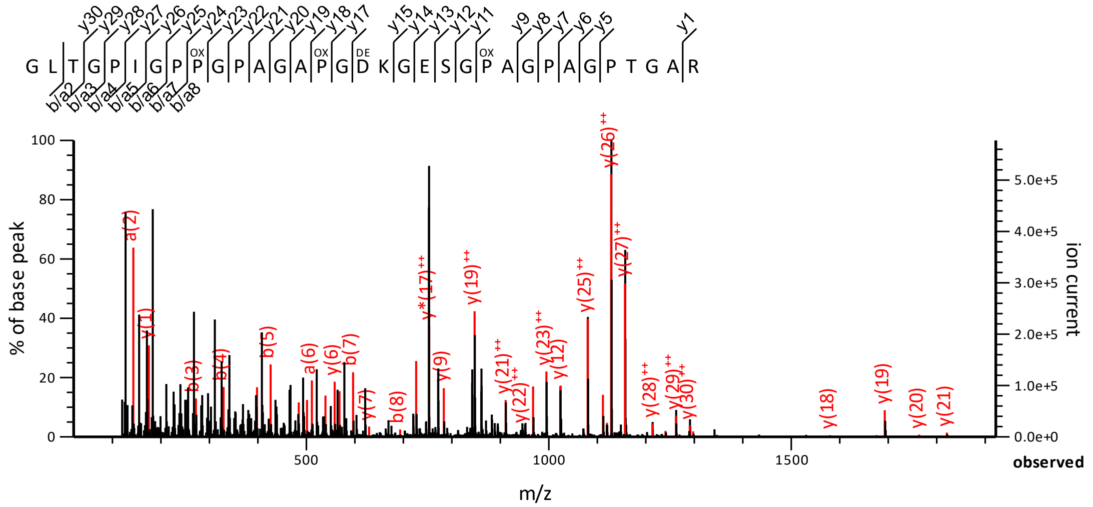

COL1A2 978 - 990

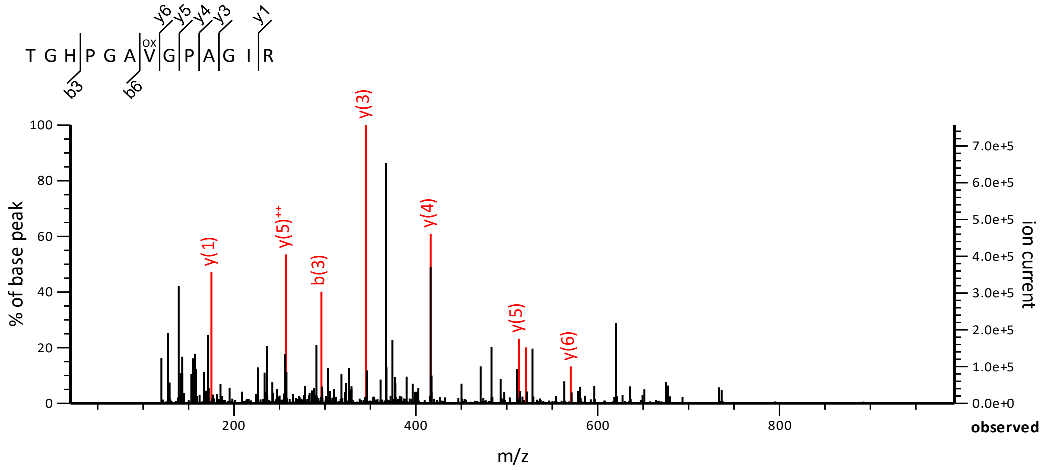

COL1A2 484 - 498

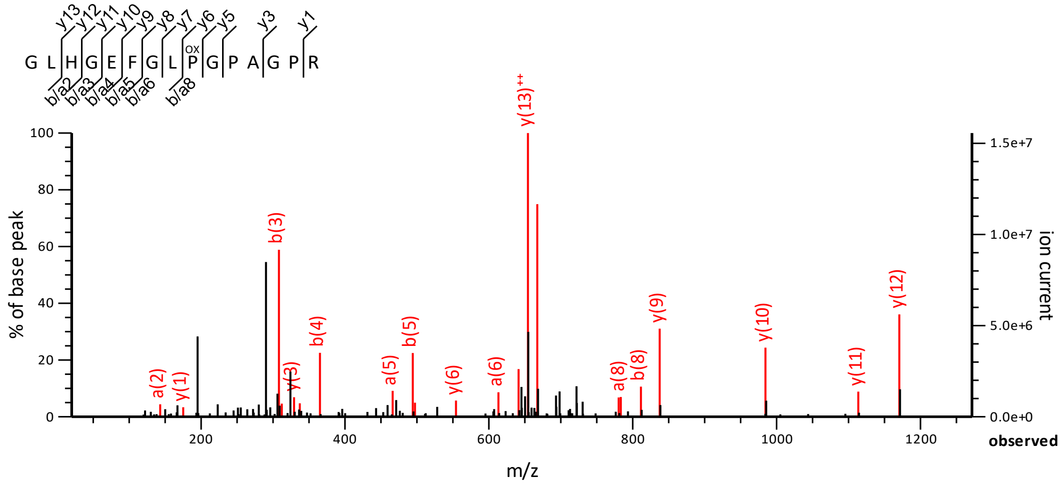

COL1A2 889 - 906

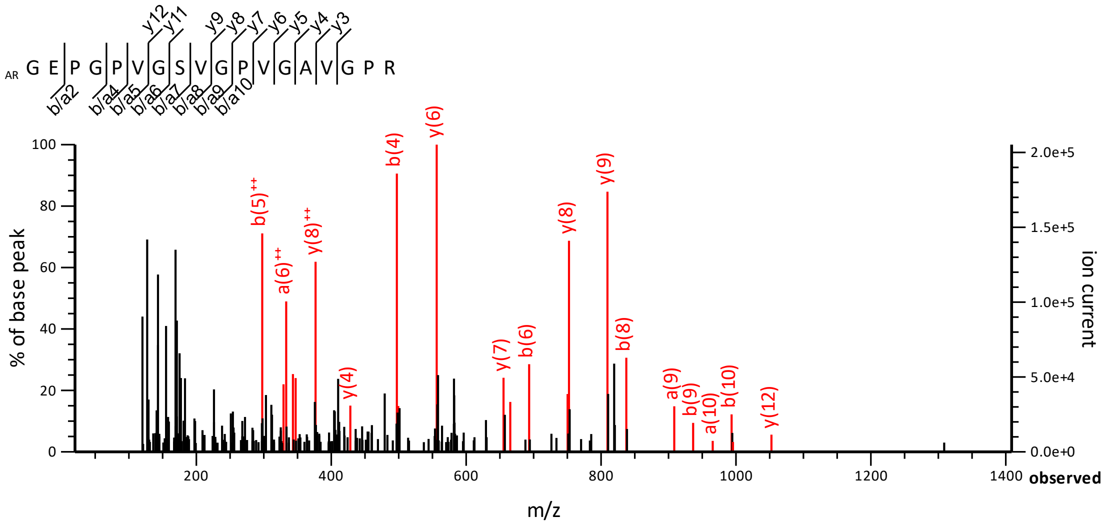

COL1A2 793 - 816

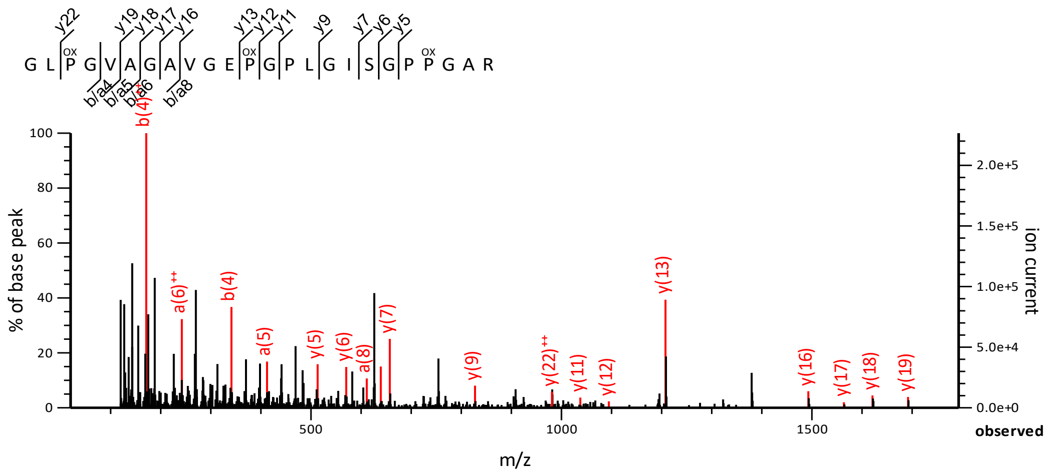

COL1A2 658 - 687

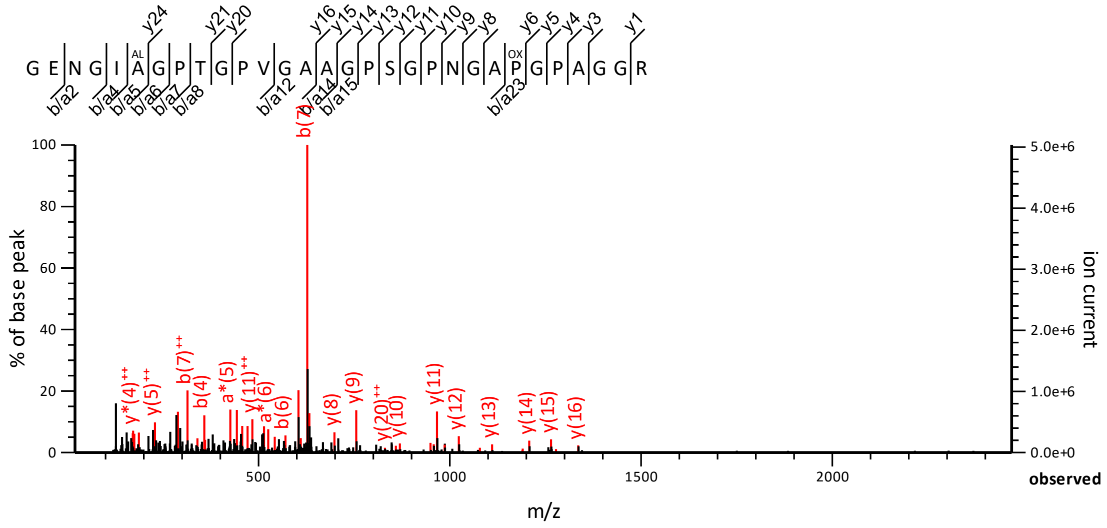

COL1A2 757 - 789

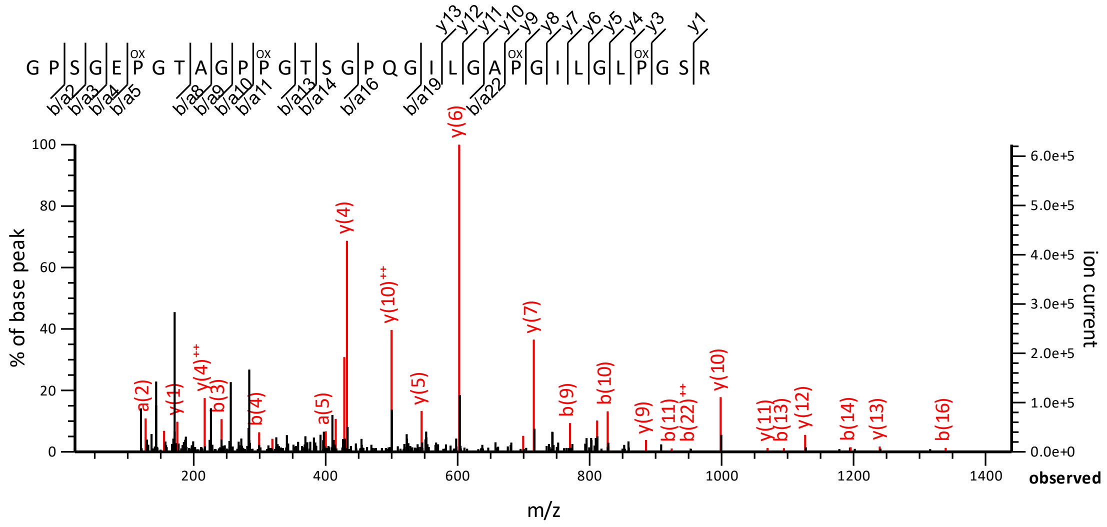

COL1A2 10-42

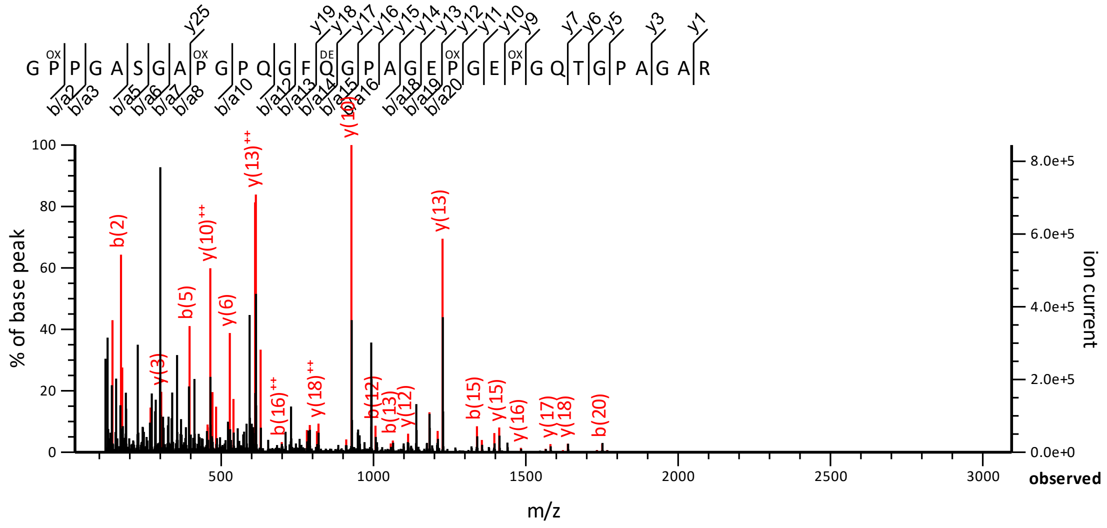

Tamandua tetradactyla

COL1A1 508 - 519

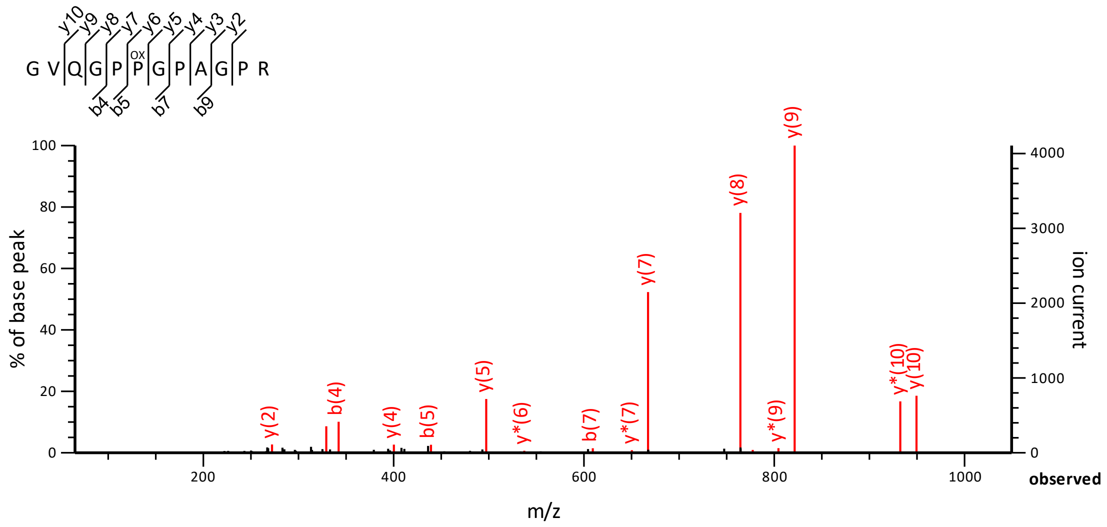

COL1A1 688 - 704

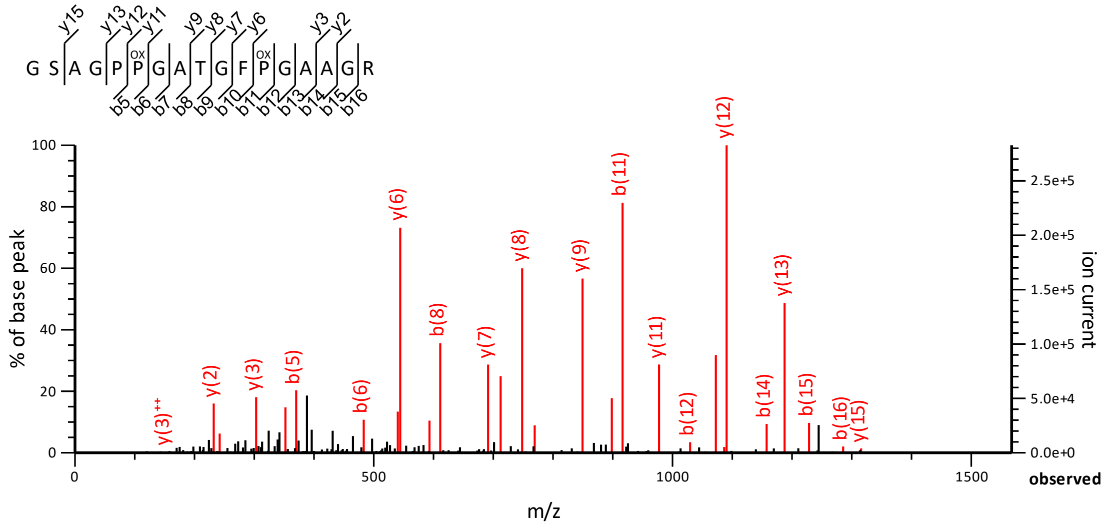

COL1A1 220 - 237

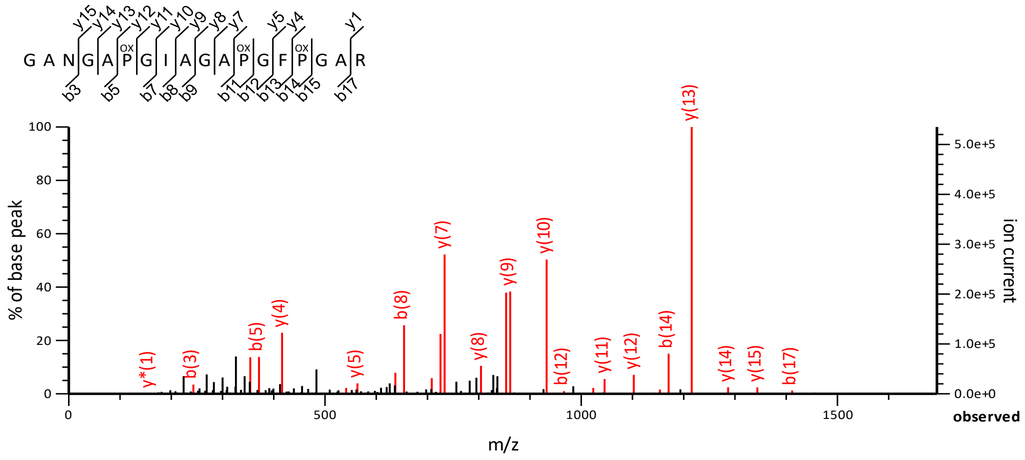

COL1A1 934 - 963

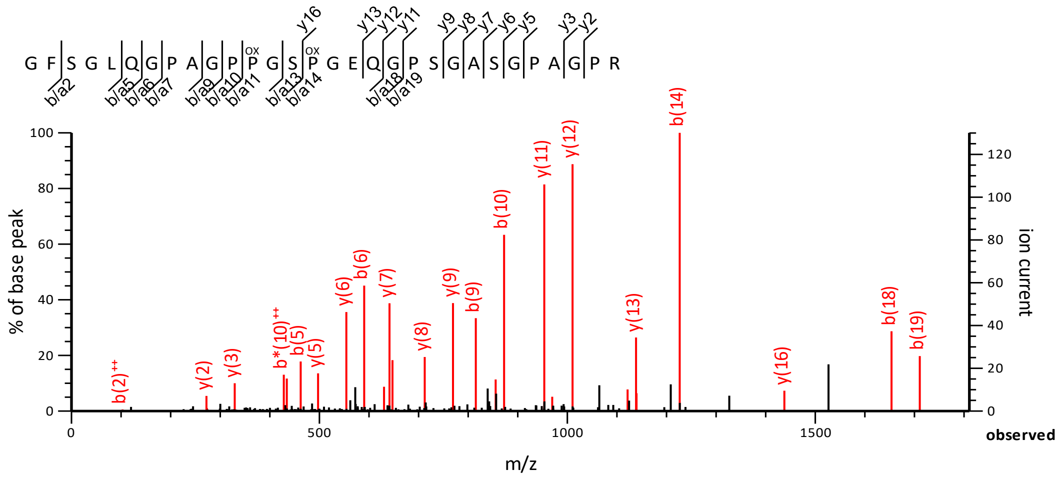

COL1A1 586 - 618

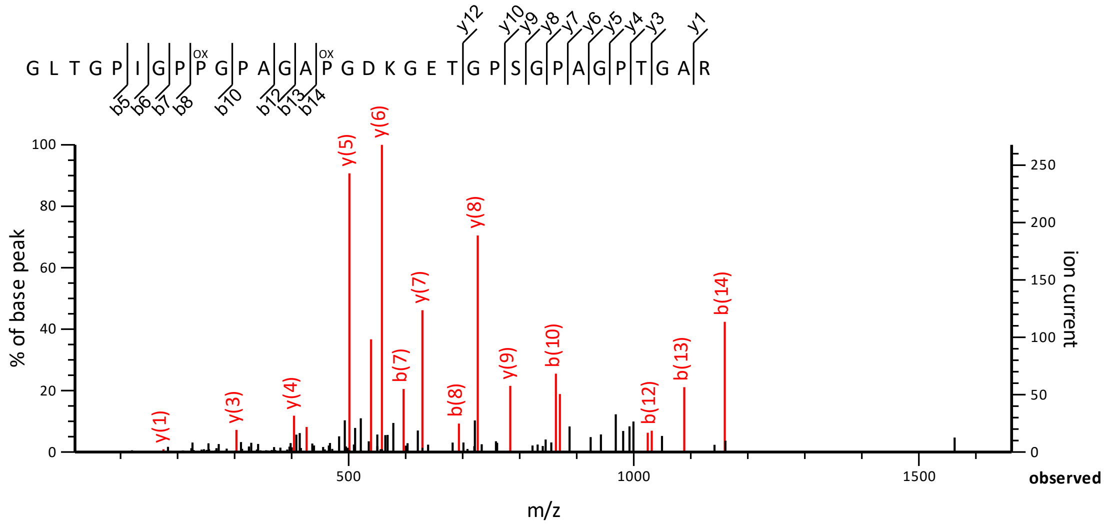

COL1A2 978 - 990

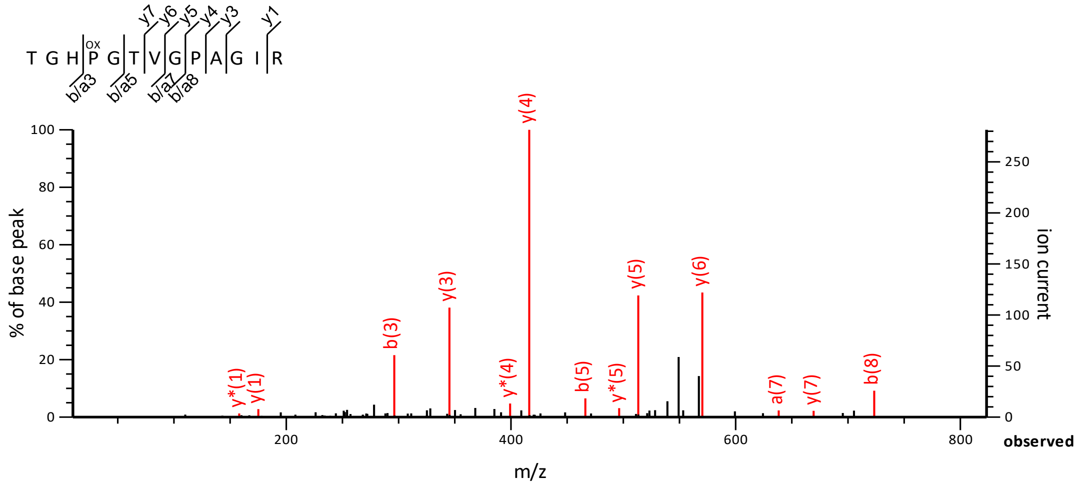

COL1A2 484 - 498

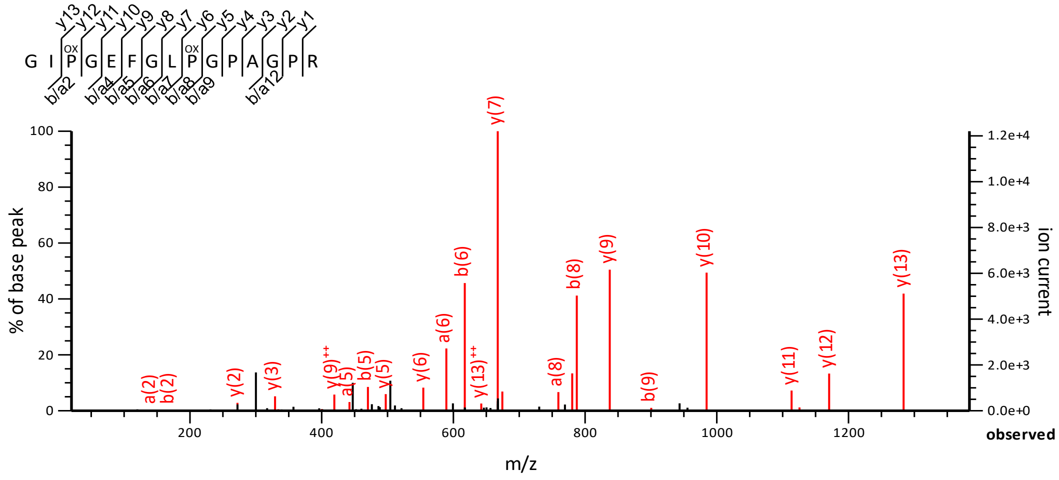

COL1A2 889 - 906

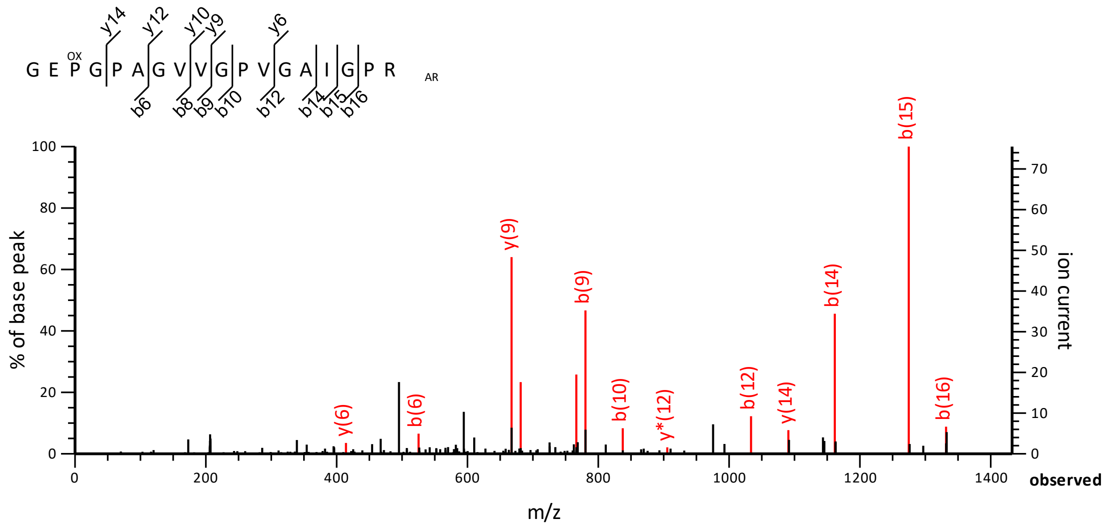

COL1A2 793 - 816

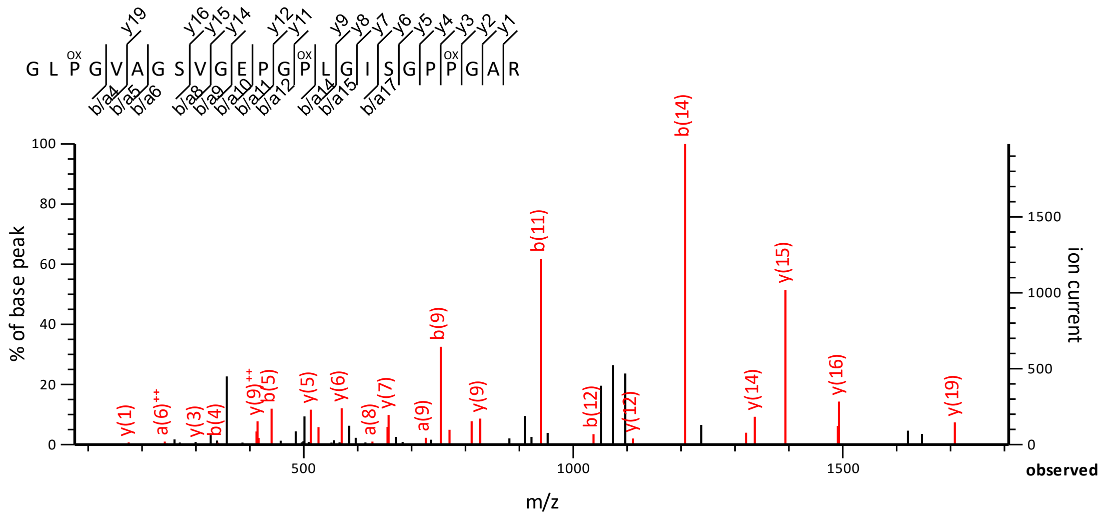

COL1A2 10-42

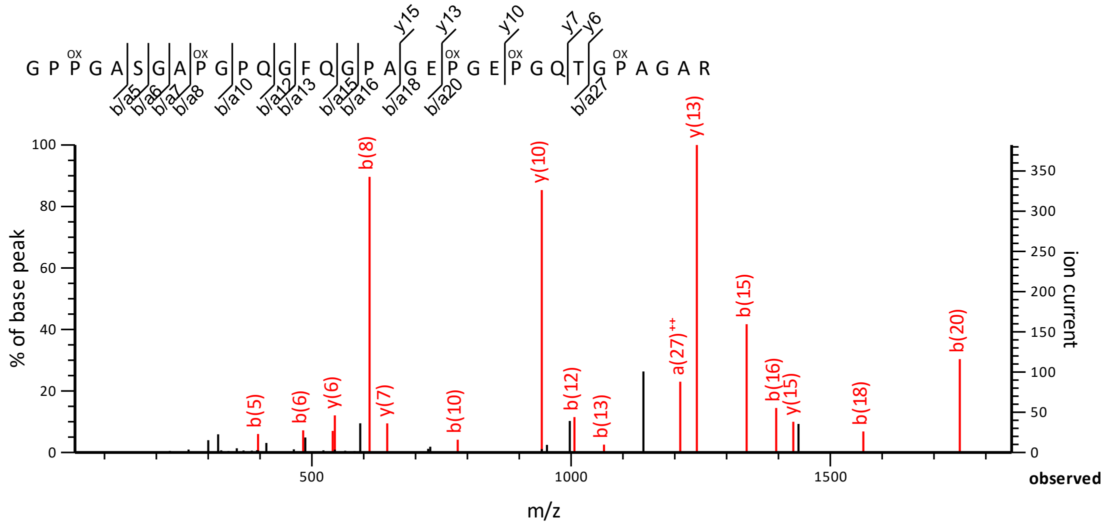

Tolypeutes matacus

COL1A1 508 - 519

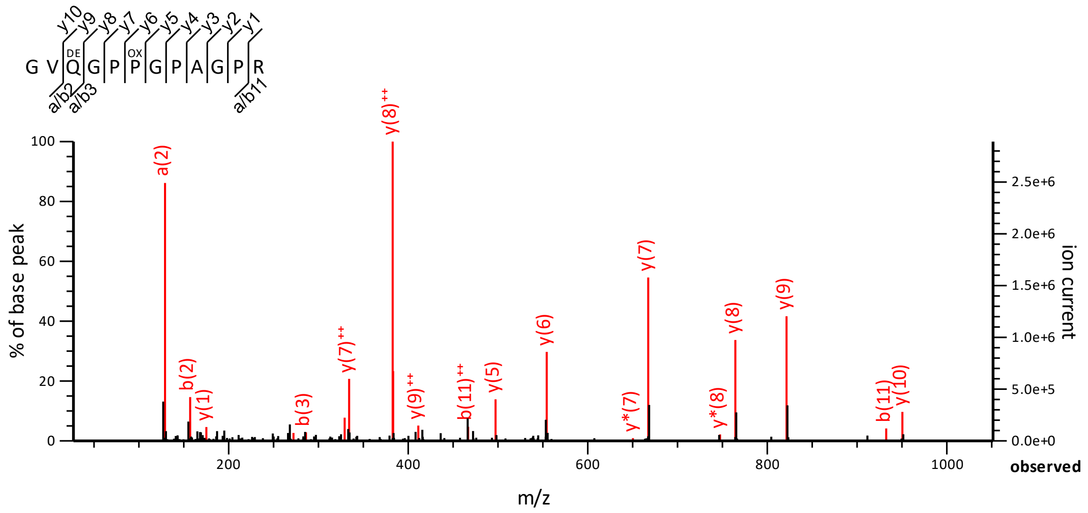

COL1A1 688 - 704

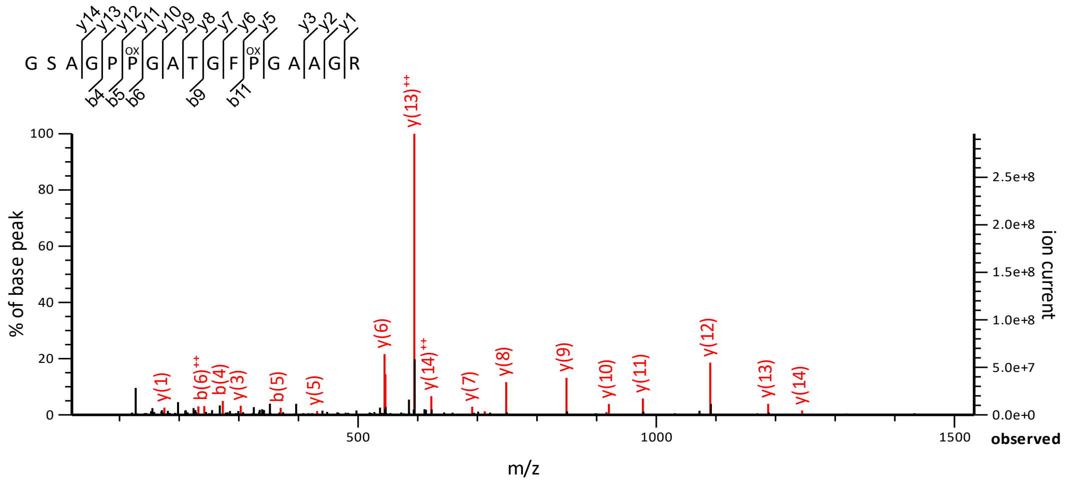

COL1A1 220 - 237

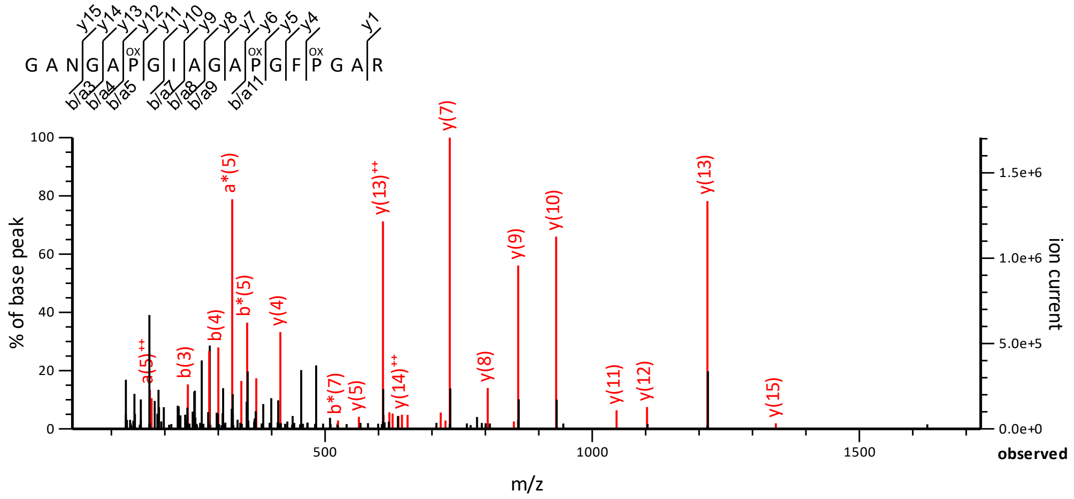

COL1A1 934 - 963

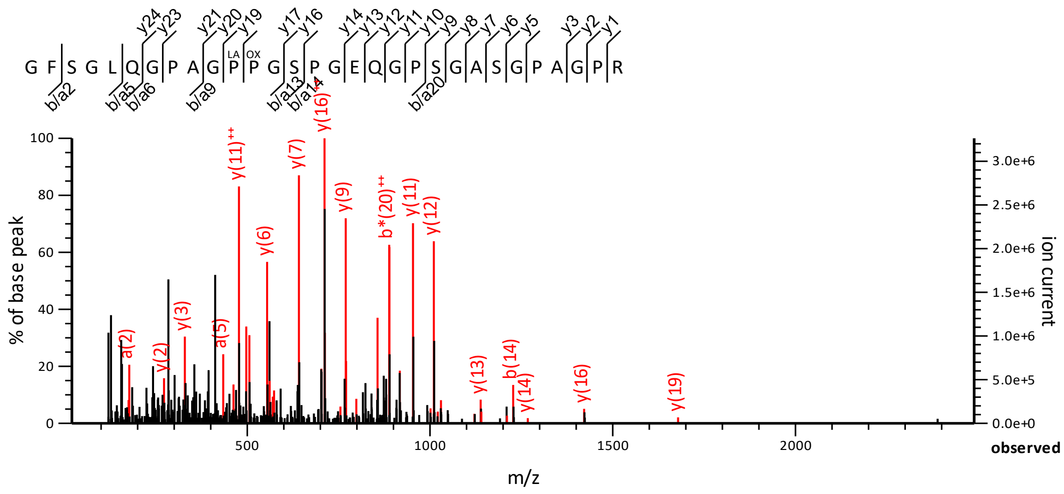

COL1A1 586 - 618

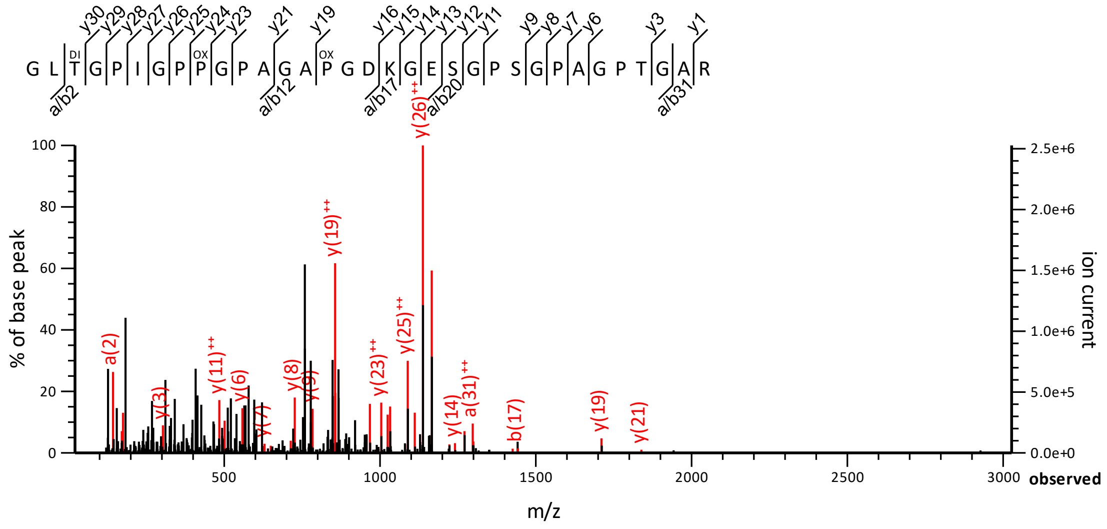

COL1A2 978 - 990

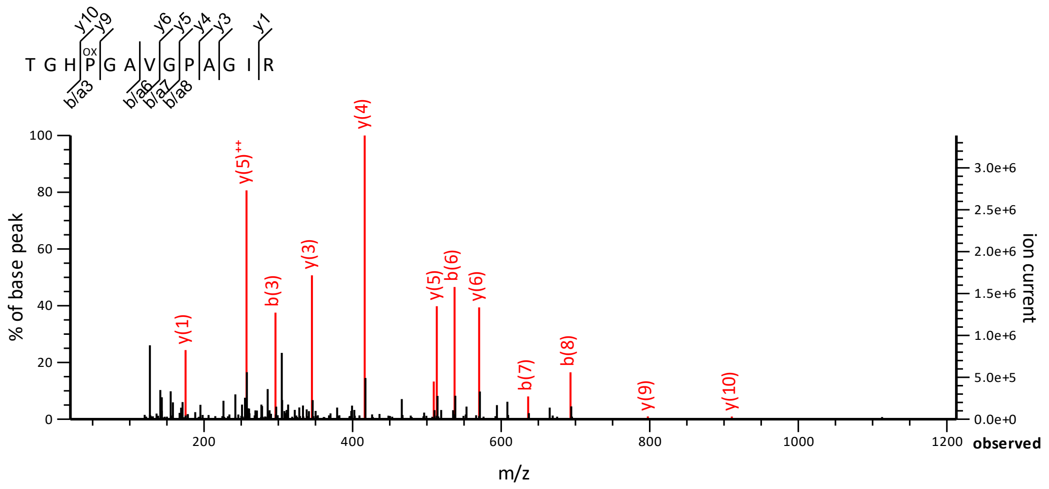

COL1A2 484 - 498

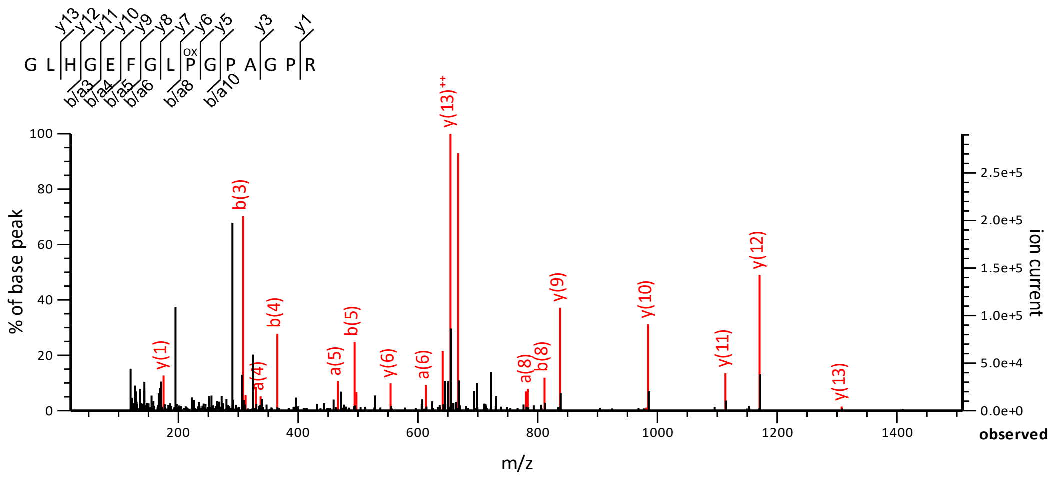

COL1A2 889 - 906

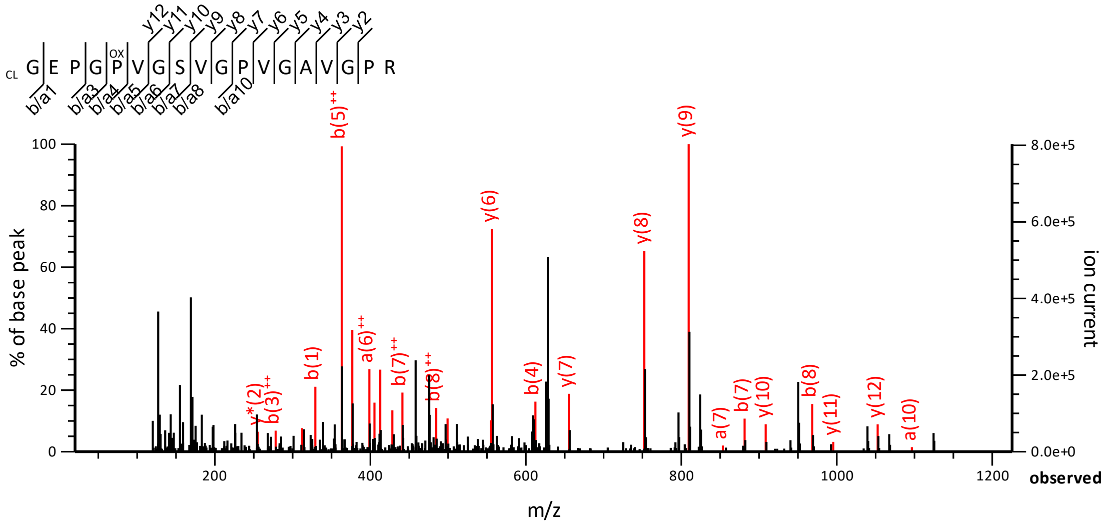

COL1A2 793 - 816

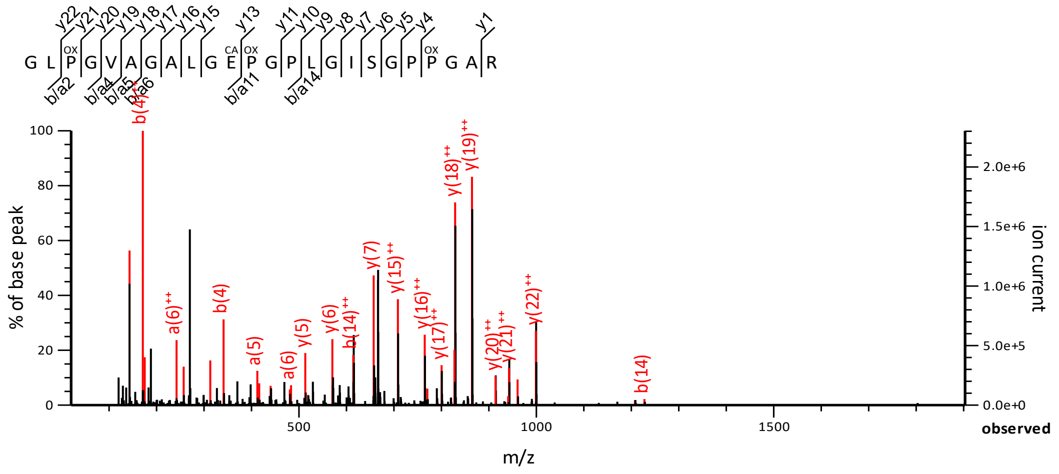

COL1A2 658 - 687

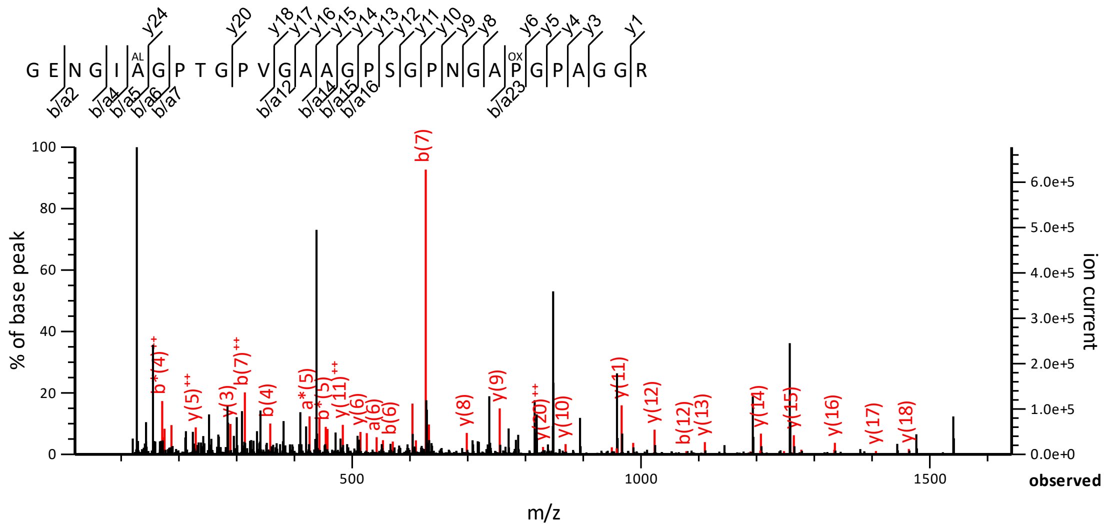

COL1A2 757 - 789

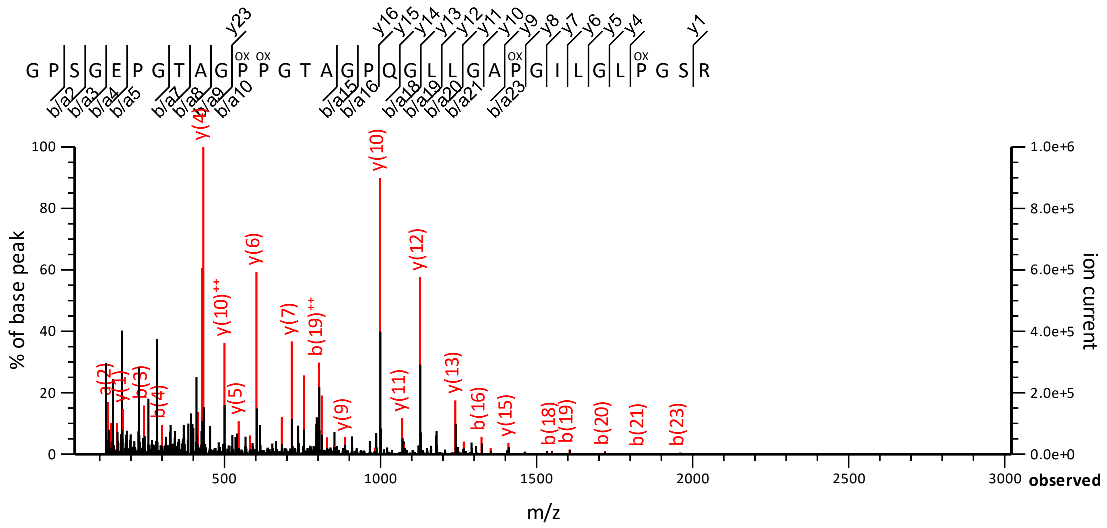

COL1A2 10-42

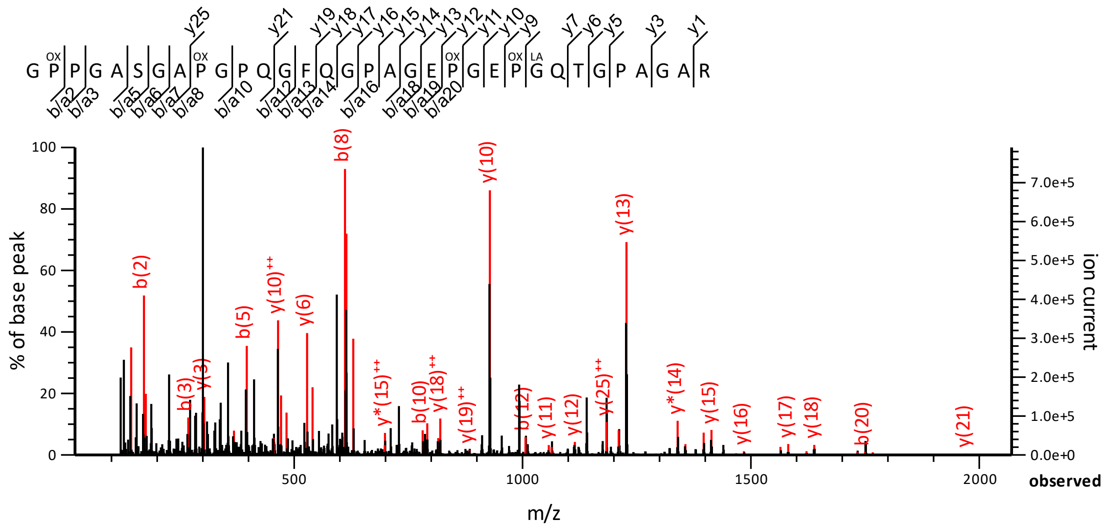

Zaedyus pichiy

COL1A1 508 - 519

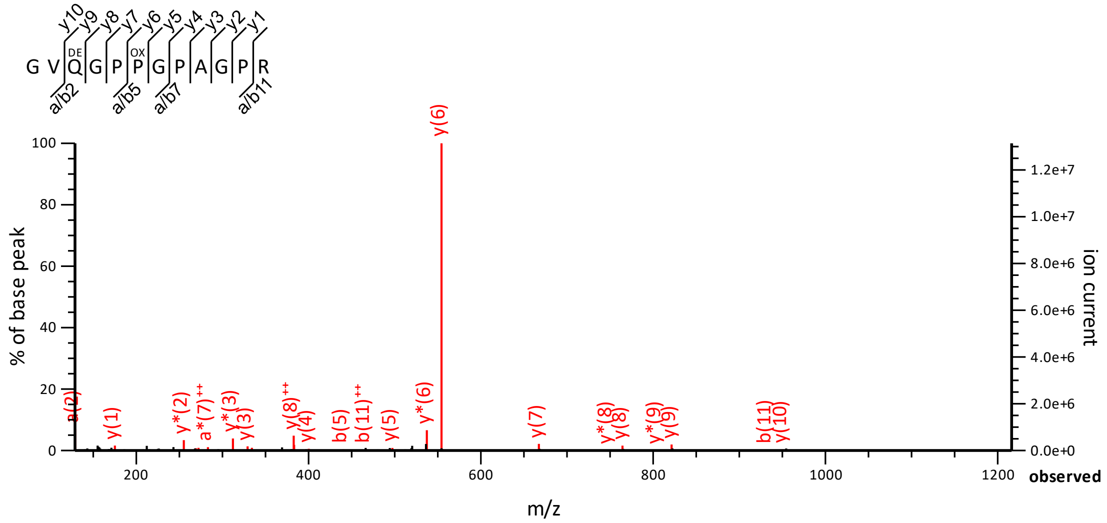

COL1A1 688 - 704

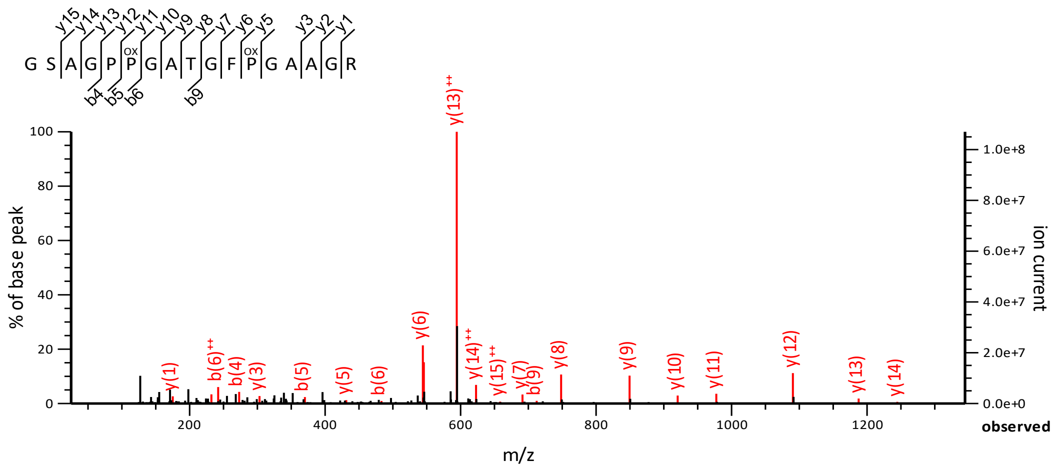

COL1A1 220 - 237

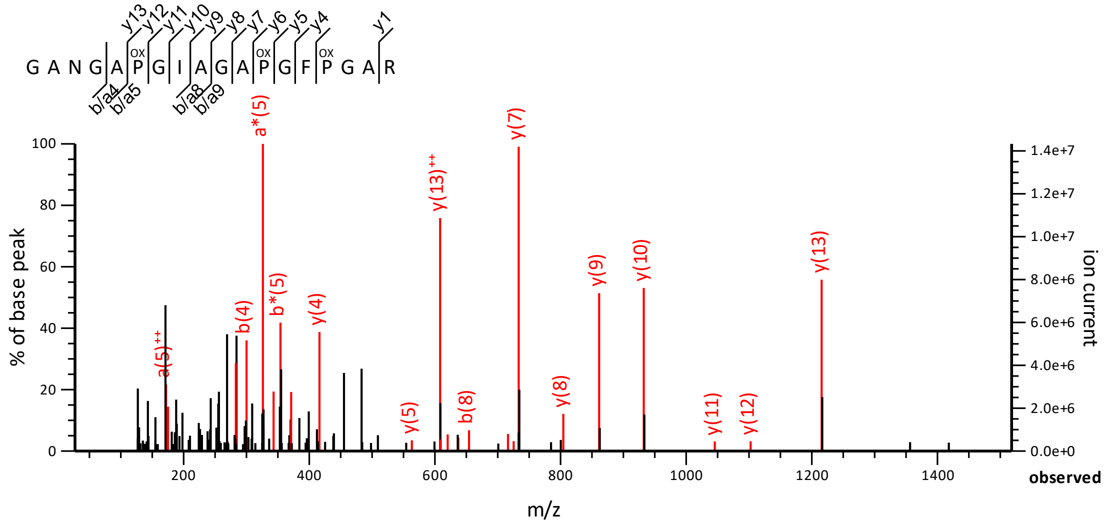

COL1A1 934 - 963

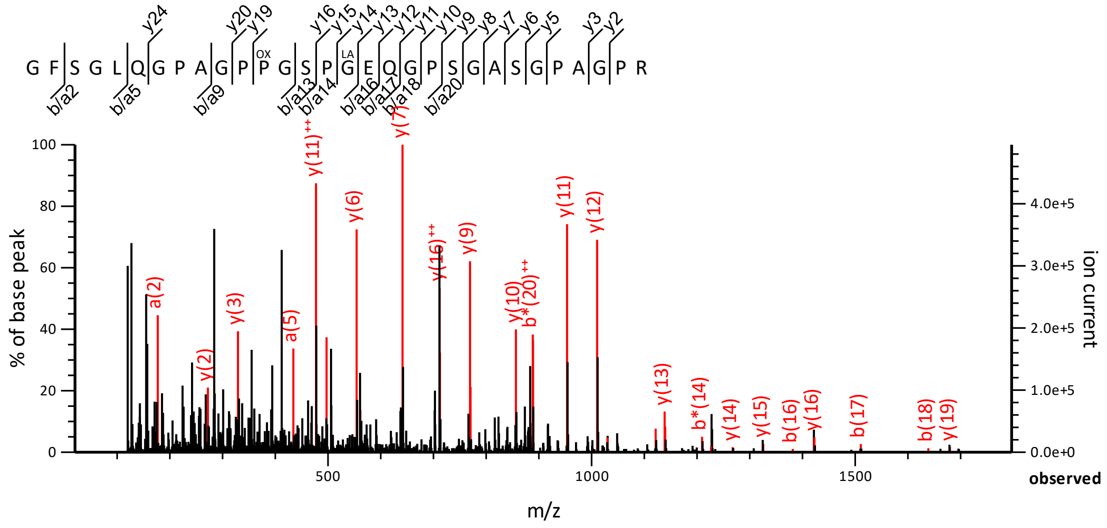

COL1A1 586 - 618

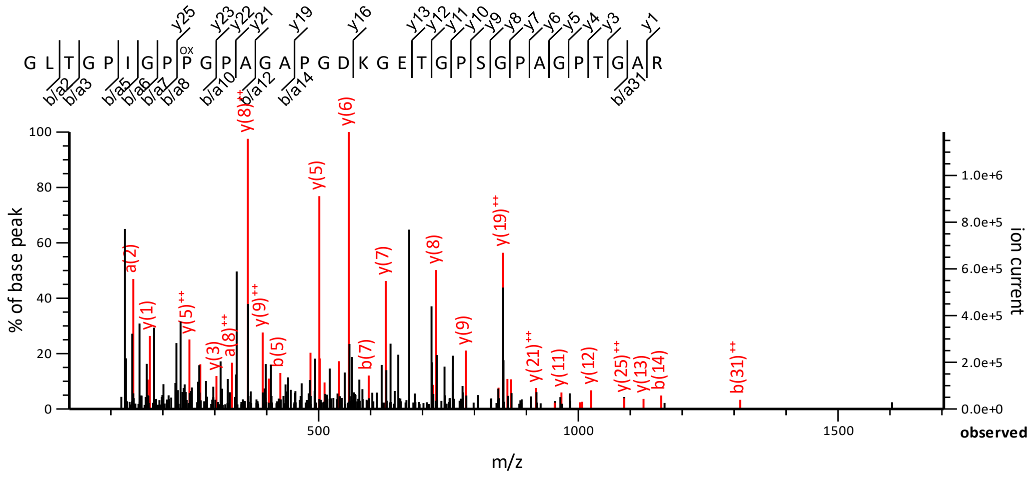

COL1A2 978 - 990

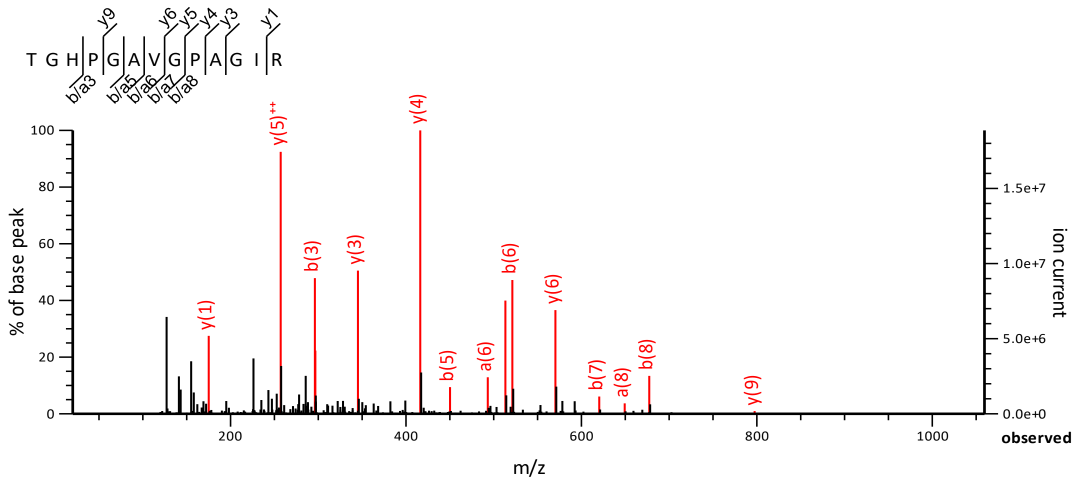

COL1A2 484 - 498

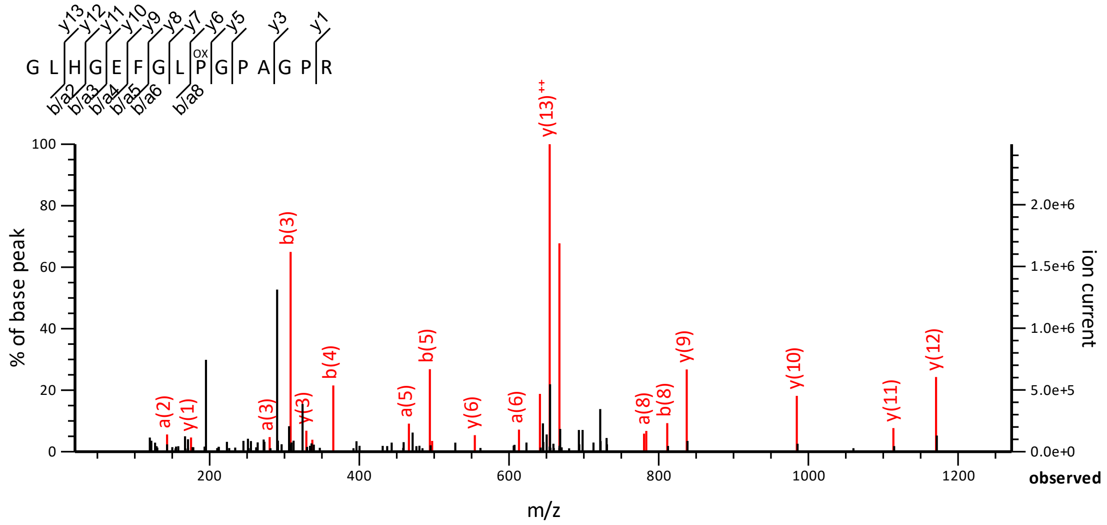

COL1A2 889 - 906

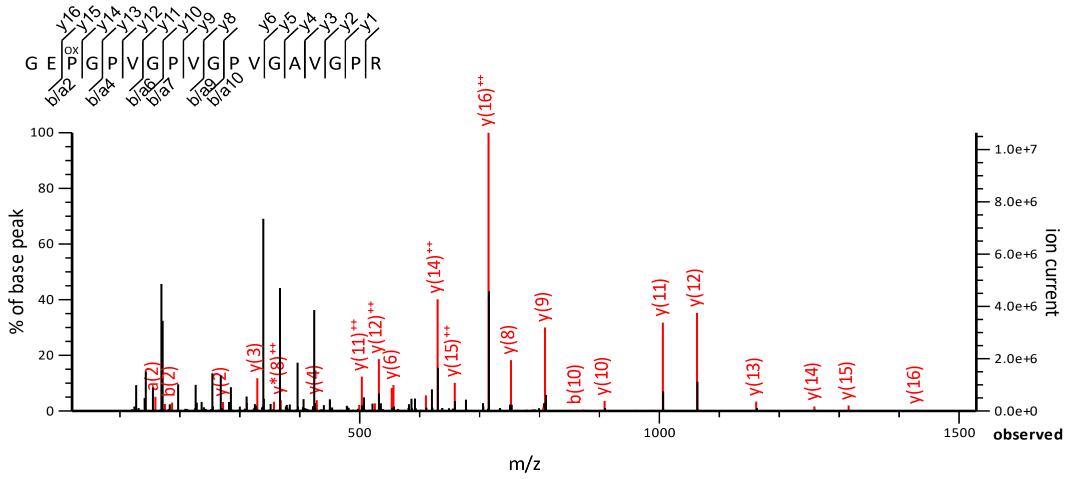

COL1A2 793 - 816

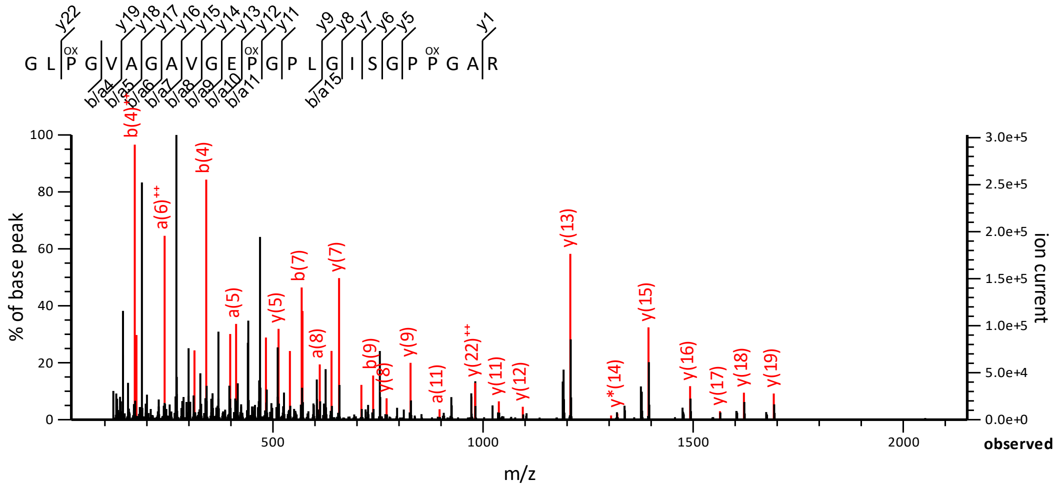

COL1A2 658 - 687

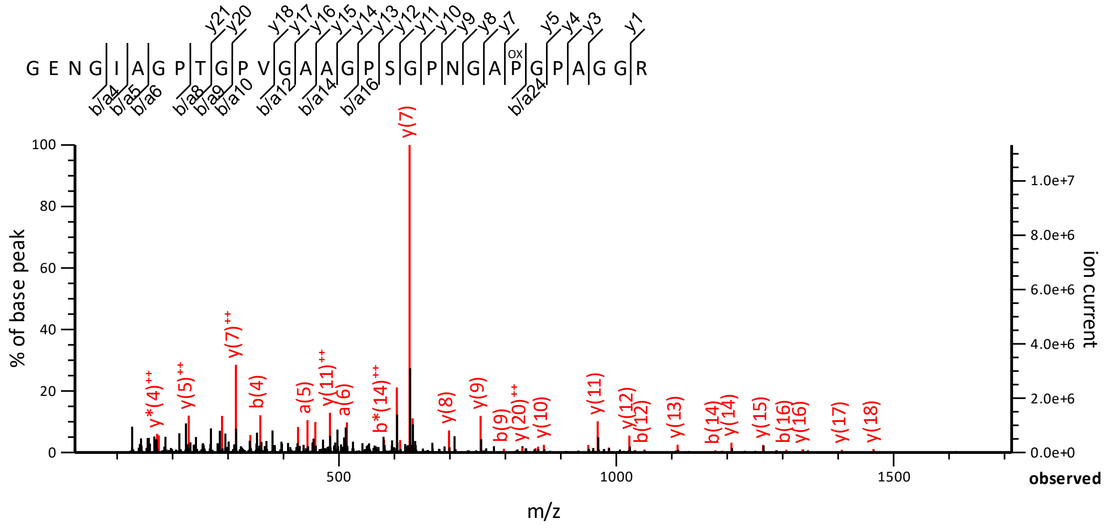

COL1A2 757 - 789

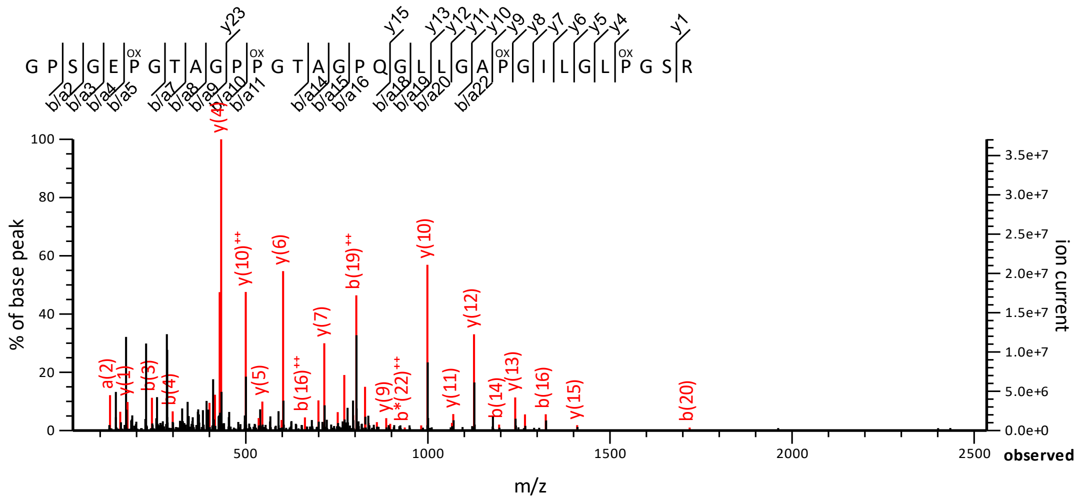

COL1A2 10-42

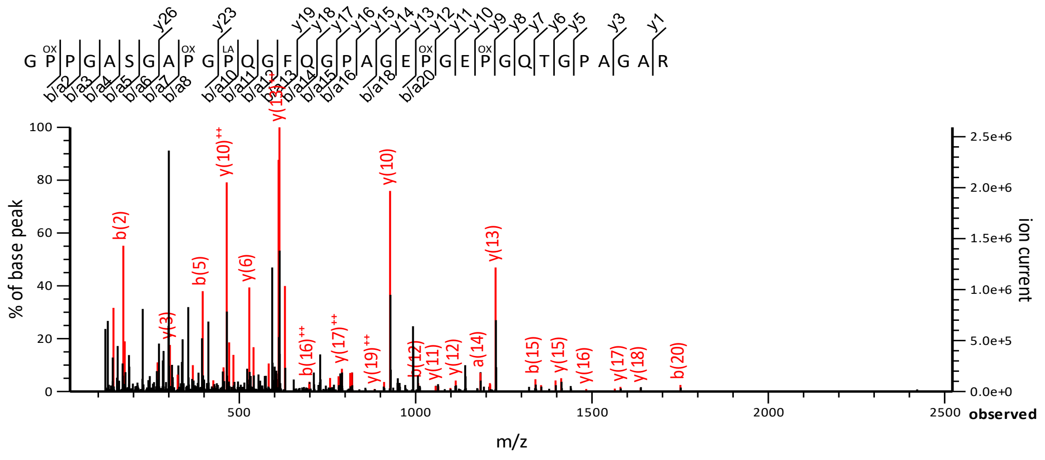

Supplement: Supplementary file 2 [file pr5c00636_si_003.pdf]
